# Simultaneous activation and release of a microtubule inhibitor from amphiphilic polymeric nanoparticles

**DOI:** 10.1039/d6na00763e

**Published:** 2026-10-07

**Authors:** Harshit Singh, Anand Kumar Thotakura, A. P. Prakasham, Esmee de Korver, Anja R. A. Palmans

**Affiliations:** a Department of Chemical Engineering & Chemistry and Institute for Complex Molecular Systems, Eindhoven University of Technology P.O. Box 513 5600 MB Eindhoven The Netherlands a.palmans@tue.nl; b The Russell H. Morgan Department of Radiology and Radiological Sciences, Johns Hopkins University School of Medicine Baltimore MD 21287 USA

## Abstract

Light addressable drug delivery systems offer opportunities to achieve spatiotemporal control over therapeutic activity while minimising off-target effects. Here we report the design, synthesis, and evaluation of a photocaged photostatin (proPST-1), in which the phenolic group of photostatin-1 (PST-1) was masked with an *o*-nitrobenzyl (ONB) photocage. Our strategy combines reversible photoswitching of the azobenzene unit with irreversible photolysis of the ONB group, affording active PST-1. Molecular docking studies suggested reduced predicted tubulin compatibility of proPST-1 due to the bulky ONB group relative to PST-1 thus silencing the dark state activity and enabling optical ON-switching. Encapsulation of proPST-1 within polymeric nanoparticles (PNs) formed from an amphiphilic polymer resulted in water soluble nanocarriers with high encapsulation efficiencies of proPST-1 (∼95%). Upon 365 nm irradiation, photolysis released the active *cis*-PST-1, weakening hydrophobic partitioning and promoting release from the nanoparticle core. *In vitro* tubulin polymerisation assays confirmed pronounced light-dependent inhibition, while cytotoxicity assays in cells validated concentration-dependent activity only after irradiation. This proof-of-concept study establishes a blueprint for integrating photocaging and photoswitching within nanocarriers to achieve dual activation and triggered release.

## Abstract 2

A photocaged derivative of the microtubule inhibitor PST-1 was designed to simultaneously suppress biological activity and enhance encapsulation within polymeric nanoparticles. Upon irradiation, drug release and tubulin inhibition is restored.

## Graphical abstract

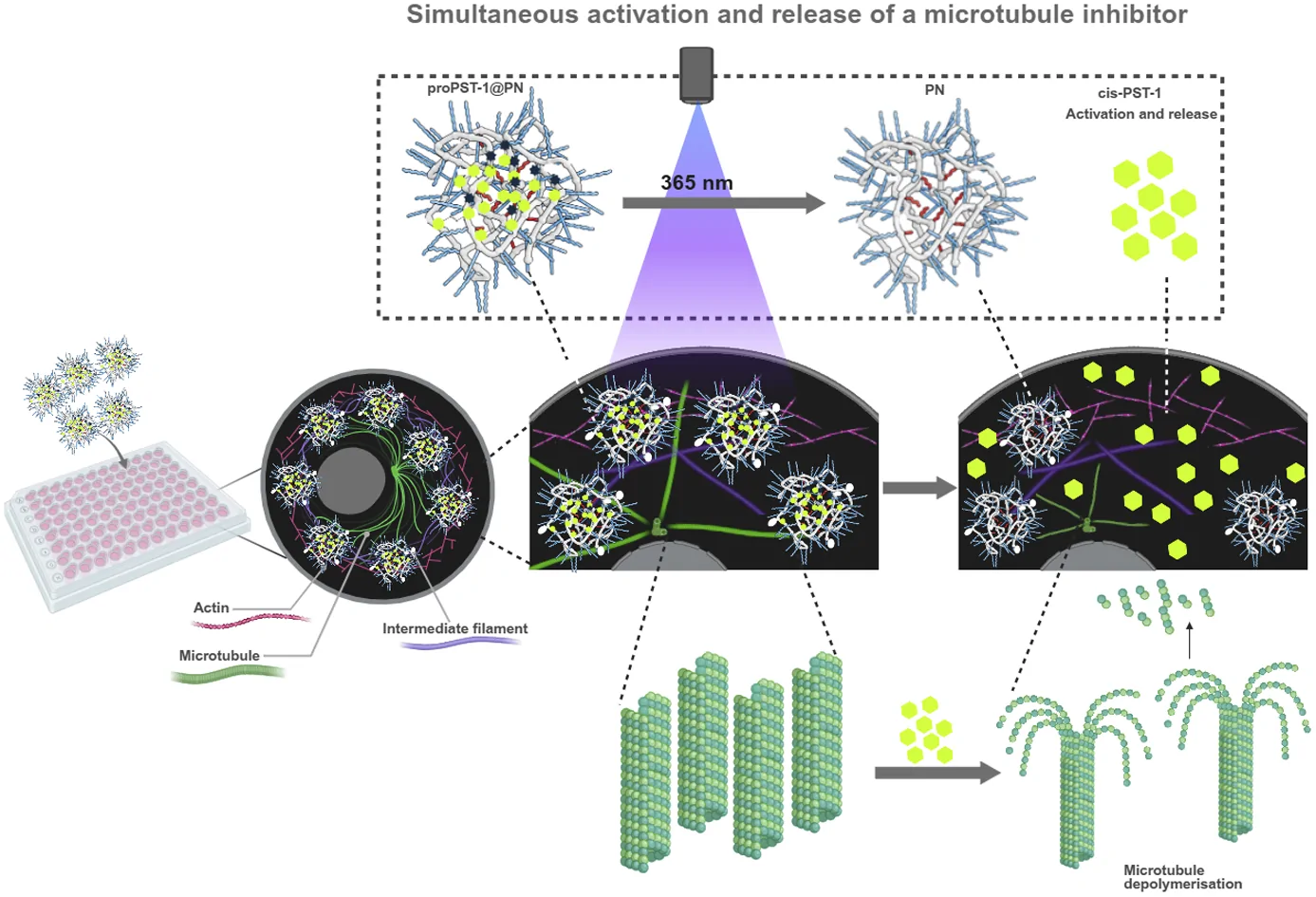

## Introduction

Polymer nanoparticles (PNs) are attractive nanocarriers for drugs because their surface chemistry, composition, size and shape can be tuned with high precision.^1–4^ Within the 5–20 nm size range of soft nanomaterials, single-chain polymeric nanoparticles (SCPNs) have emerged as a distinct class of nanocarriers for biomedical applications in drug delivery and bio-orthogonal catalysts.^5–10^ SCPNs are formed through intramolecular collapse of single random amphiphilic heterograft polymer chains in aqueous media, driven by hydrophobic interactions that promote the formation of compact nanoscale structures.^11,12^ Unlike micelles, vesicles, and polyelectrolyte complexes, SCPNs preserve particle identity upon dilution and therefore, in complex media, the kinetic fragility and uncontrolled disassembly of the nanostructure is circumvented.^13,14^ Previously, our group has utilised SCPNs as artificial enzymes by confining catalytic sites in a dynamic yet well-defined pocket, enhancing the activity and selectivity of metal catalysts in complex environments.^15–18^ In the context of nanomedicine, SCPNs offer advantages in terms of surface functionalisation and targeting.^19^ The unimolecular nature of SCPNs enables precise modulation of surface charge, hydrophilicity, and ligand density while maintaining the structural integrity of the nanostructure.^20,21^ This facilitates the introduction of targeting ligands, or responsive functionalities, allowing cellular uptake,^22^ targeting,^23^ cytosolic delivery,^24–26^ photodynamic therapy,^26,27^ biosensing,^28^ imaging,^29,30^ drug conjugation,^10^ and drug release.^31–34^ The dynamic interior of SCPNs enables adaptive reorganisation in response to guest molecules while maintaining spatial confinement. Non-covalent interactions such as hydrophobic effects, π–π stacking, and hydrogen bonding govern cargo binding and release, offering opportunities to modulate host guest interactions through molecular design.^35^ Despite these advantages, externally controllable cargo release from SCPNs remains underdeveloped relative to multichain nanocarriers. Establishment of a general design paradigm that couples a well-defined physicochemical trigger to a measurable change in carrier mechanics and therapeutic function would substantially expand the utility of SCPNs in precision therapeutics and biomedical settings.

Among external stimuli, light is uniquely attractive due to its high spatiotemporal precision, tunable wavelength, and orthogonality to endogenous biochemical pathways.^36^ Light responsive drug delivery enables non-invasive activation with fine spatial control, allowing therapeutic activity to be restricted to specific tissues or cellular regions.^37^ Two principal photochemical strategies dominate this field: photoswitching and photocaging.^38^ Photoswitchable drugs undergo reversible conformational changes upon irradiation, commonly through cis–trans isomerisation of photochromic units such as azobenzene derivatives, enabling dynamic modulation of biological activity.^39,40^ However, incomplete population of the inactive isomer frequently results in residual dark activity, limiting therapeutic contrast. Photocaging strategies provide a complementary approach, relying on irreversible photolysis of a masking group that suppresses biological activity until light exposure liberates the active compound.^41,42^ Phenolic photocages based on the *o*-nitrobenzyl (ONB) scaffold represent one of the most widely used and robust classes of photocaging groups. ONB derivatives undergo bond cleavage upon irradiation with UV light (∼365 nm), releasing benign by products and unmasking functional groups commonly present in drugs and biomolecules.^43,44^ In addition to their reliable photochemistry, ONB groups are bulky and hydrophobic, properties that can enhance encapsulation within hydrophobic nanocarrier interiors and improve guest retention prior to activation. Photoswitchable microtubule inhibitors exemplify the potential of optical control in chemical biology. Photostatins, azobenzene analogues of combretastatin A-4 (CA-4)^45^ (Scheme 1), undergo reversible cis–trans isomerisation that modulates binding to the colchicine site on tubulin. Photostatin-1 (PST-1) exhibits significantly higher potency in its cis configuration relative to the trans isomer, enabling reversible optical control over microtubule dynamics.^46–48^ Nevertheless, residual dark activity arising from incomplete photoswitching remains a limitation, particularly for applications requiring stringent off-state suppression.

**Scheme 1:**
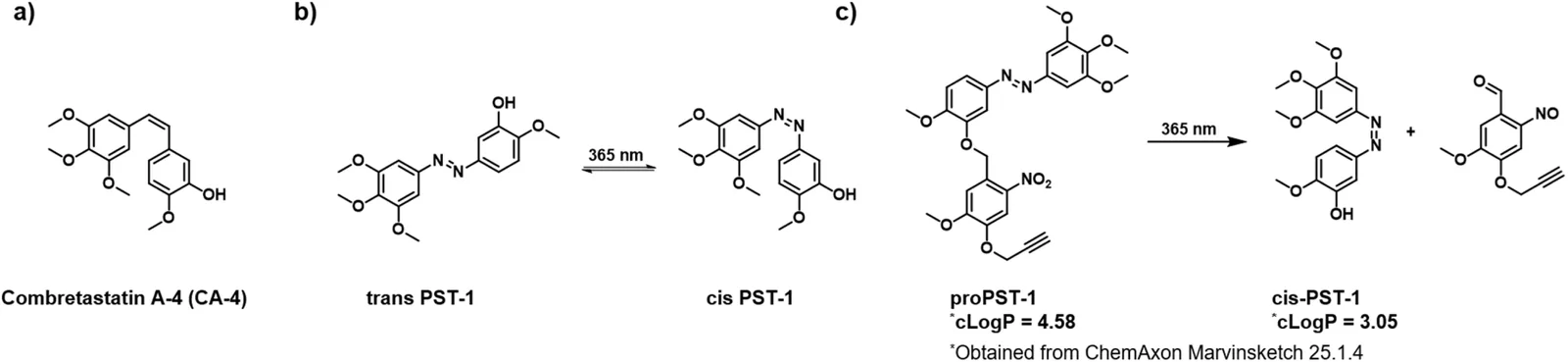
(a) Chemical structure of combretastatin A-4 (CA-4), (b) photoisomerisation of PST-1, (c) strategy for simultaneous activation and release of PST-1 from proPST-1 upon irradiation.

Herein, we combine photocaging chemistry with SCPN-based encapsulation to achieve light-controlled drug release from amphiphilic polymeric nanoparticles. An ONB group is installed on the phenolic handle of PST-1 to generate an inactive photocaged prodrug (proPST-1), which undergoes clean photolysis upon 365 nm irradiation to release active PST-1 (Scheme 1). The photocaged photostatin is subsequently mixed with SCPNs to yield a water soluble, light-addressable polymeric nanocarrier. This design is guided by the hypothesis that photolysis-induced changes in payload polarity^49^ and non-covalent interaction profiles weaken hydrophobic partitioning within the nanoparticle core, thereby triggering release without carrier disassembly. In the dark, the hydrophobic ONB-protected proPST-1 preferentially partitions into the nanoparticle interior, whereas photodeprotection generates a more polar active drug with reduced affinity for the hydrophobic domain. In this manner, light functions both as an activation trigger and as a molecular switch that directly couples photochemistry to SCPN carrier mechanics.

## Experimental section

### Materials

All commercial reagents were purchased from Merck, Thermo Fisher Scientific and BLDpharm, and were used without further purification. Dry solvents were obtained from an MBRAUN Solvent Purification System (MB-SPS). Bulk solvents were purchased from Biosolve and deuterated solvents (CDCl_3_ from Cambridge Isotope Laboratories and CD_3_CN from Deutero GmbH) were purchased and used as received. HeLa (ATCC-CCL-2) and SH-SY5Y (ATCC-CRL-2266) cells were obtained from ATCC. Dulbecco's Modified Eagle's Medium (DMEM) and fetal bovine serum (FBS) were purchased from Thermo Fisher Scientific. Cell counting kit-8 (CCK-8) assay kit was purchased from Sigma-Aldrich. Tubulin MAP-rich (porcine brain) (Cat. #ML116), Tubulin protein (fluorescent HiLyte 488): porcine brain (Cat. #TL488M), General tubulin buffer (Cat. #BST01), Tubulin glycerol buffer (Cat. #BST05) and Taxol (Cat. #TXD01) and GTP (Cat. #BTS06) were obtained from Cytoskeleton, Inc and used as per the instruction manual. Dialysis tubing (1 kDa MWCO) was obtained from fisher scientific.

### Measurements and characterisations

NMR spectra were recorded on Bruker Avance III 400 MHz spectrometer. ^1^H and ^13^C NMR signals are reported in ppm downfield from TMS. ^1^H signals are referenced to the residual proton of a deuterated solvent 7.26 ppm for CDCl_3_ and 1.94 ppm for CD_3_CN. ^13^C{^1^H} signals are referenced to the solvent signal at 77.16 ppm for CDCl_3_. ^1^H NMR peaks are labelled as singlet (s), doublet (d), and multiplet (m). Matrix-assisted laser desorption/ionisation time-of-flight followed by mass spectrometry (MALDI-ToF-MS) measurements were performed on a Bruker Autoflex Speed using *α*-cyano-4-hydroxycinnamic acid (CHCA) and *trans*-2-[3-(4-*tert*-butylphenyl)-2-methyl-2-propenylidene]malononitrile (DCTB) as matrices. LCMS (ESI) measurements were performed on LTQ XL linear ion trap Thermo Fisher Scientific mass spectrometer. Amicon® Ultra-15 Centrifugal Filter Units were used for filtering polymers from prodrugs. UV-Vis measurements were performed on Agilent Cary 3500 multicell UV-Vis spectrophotometer using 1 cm pathlength quartz cuvettes. Malvern Zetasizer with 830 nm laser at 180° was used for dynamic light scattering experiments and samples were filtered over a 0.2 µm PVDF filter prior to measurements to remove any dust particles from hindering the measurements. Total Internal Reflection Fluorescence (TIRF) microscopy was performed on Nikon N-STORM microscopy system *via* excitation of the sample in green channel (561 nm laser, 10% intensity). Cell viability was assessed by measuring the absorbance on Tecan SPARK microplate reader.

### *In Silico* molecular docking studies

The structures of compounds (*cis*-PST-1, *trans*-PST-1, *cis*-proPST-1, *trans*-proPST-1) were built with standard bond lengths and angles using ChemDraw and then energy minimised with Chem3D using the integrated MM2 energy minimisation script. The X-ray crystal structure of Tubulin-Combretastatin A4 complex (PDB ID: 5LYJ; resolution = 2.4 Å) was retrieved from PDB. The protein and ligand PDBQT files were prepared using AutoDock Tools 1.5.6 (ADT) following the standard protocol.^50^ Molecular docking simulations were performed using AutoDock Vina with the following docking parameters: grid box (17.2 × 20 × 16.8 Å^3^) at the coordinates (*x* = 13.375, *y* = 7.388, *z* = −22.645) with settings: exhaustiveness = 64, energy range = 3 kcal mol^−1^ and number of modes = 20. To assess the reproducibility of the predicted binding poses, ten replicate docking runs were performed for each ligand, with 20 poses generated per run. The lowest-energy pose from the first run was used as the reference and compared with the lowest-energy pose from each subsequent run by heavy-atom root-mean-square deviation (RMSD) analysis in PyMOL. An RMSD of ≤2.0 Å was used as a practical criterion to assess the reproducibility of the predicted binding mode. To further assess the robustness of the predicted binding orientations using an independent docking/scoring approach, the ligands were additionally docked using AutoDock 4.2, with docking parameters prepared in AutoDockTools 1.5.7. The same binding region was defined using the grid-box dimensions and centre coordinates described above. Docking was performed using the Lamarckian Genetic Algorithm (LGA) with a population size of 300, a maximum of 2 500 000 energy evaluations and 27 000 generations, a mutation rate of 0.02, and a crossover rate of 0.8. Local optimization was performed using the Solis–Wets algorithm with a maximum of 300 iterations and a local-search frequency of 0.06. A total of 50 independent LGA docking runs were performed for each ligand. The resulting conformations were clustered using an RMSD tolerance of 2 Å. The lowest-energy pose from each docking run was evaluated, and the global minimum-energy conformation was selected for detailed interaction analysis. Structural visualisation and figure generation were carried out using PyMOL (The PyMOL Molecular Graphics System, Version 3.0 Schrõdinger, LLC).

### Synthesis and characterisation

1-(Bromomethyl)-5-methoxy-2-nitro-4-(prop-2-yn-1-yloxy)benzene (ONB-Br)^51^ and (*E*)-2-methoxy-5-((3,4,5- trimethoxyphenyl)diazenyl)phenol (PST-1)^46^ were synthesised using reported protocols and analytical data were consistent with reported values. ^1^H and ^13^C-NMR of these molecules are attached in ESI. Amphiphilic polymer was used as synthesised and reported previously.^17^

### Synthesis of (*E*)-1-(4-methoxy-3-((5-methoxy-2-nitro-4-(prop-2-yn-1-yloxy)benzyl)oxy)phenyl)-2-(3,4,5-trimethoxyphenyl)diazene (proPST-1)

To a solution of ONB-Br (150 mg, 1 eq.), PST-1 (167 mg, 1.05 eq.) in acetonitrile (8 mL) in a round bottom flask in dark at room temperature in argon atmosphere, anhydrous K_2_CO_3_ (207 mg, 1.5 eq.) was added, and the reaction mixture was refluxed for 18 h. After complete consumption of starting material, as confirmed by TLC analysis, reaction was stopped, and reaction mixture was brought to room temperature. Acetonitrile was removed under reduced pressure, water (10 mL) was added to the reaction mixture and extracted with dichloromethane (DCM) (3× 10 mL). The organic fractions were combined, dried over anhydrous MgSO_4_ and concentrated under reduced pressure to afford a yellow crude solid. Purification by silica gel column chromatography yielded proPST-1 as a yellow solid (200 mg, 74%). ^1^H-NMR (400 MHz, CDCl_3_) *δ*(ppm): 7.95 (s, 1H), 7.67–7.65 (m, 1H), 7.59–7.58 (m, 2H), 7.23 (s, 2H), 7.05 (m, 1H), 5.65 (s, 2H), 4.85–4.84 (d, *J* = 4 Hz, 2H), 4.00–3.97 (m, 12H), 3.93 (s, 3H), 2.59–2.57 (t, *J* = 4 Hz, 1H); ^13^C-NMR (100 MHz, CDCl_3_) *δ*(ppm): 154.8, 153.7, 152.4, 148.6, 148.2, 147.0, 145.6, 140.4, 138.9, 130.7, 120.8, 111.3, 110.9, 110.2, 105.8, 100.3, 77.4, 77.3, 68.2, 61.2, 57.2, 56.5, 56.4; MALDI-ToF-MS: *m*/*z* calcd. for [C_27_H_27_N_3_O_9_ + H]^+^: 538.18, found: 538.17; calcd. for [C_27_H_27_N_3_O_9_ + Na]^+^: 560.16, found: 560.14; LCMS ESI: *m*/*z* calcd. for [C_27_H_27_N_3_O_9_ + H]^+^: 538.18, found: 538.67.

### PST-1/proPST-1 release from proPST-1@PN in PBS

The release of proPST-1 from the polymeric nanoparticles was evaluated using a dialysis method.^34^ 5 mL of proPST-1@PN solution in PBS (pH 7.4; 1 mg mL^−1^ polymer concentration; polymer : proPST-1 molar ratio = 1 : 10) was transferred into a dialysis bag with a molecular weight cutoff (MWCO) of 1 kDa. The dialysis bag was then immersed in 250 mL of PBS (pH 7.4) and maintained at 37 °C with continuous stirring under either dark or irradiated conditions. For the irradiated samples, the system was exposed to 365 nm light at an intensity of 5.5 mW cm^−2^ for 5 min per irradiation cycle for a total of 24 times in 48 h, whereas the corresponding control was protected from light throughout the experiment. At predetermined time points, aliquots (2 mL) of the external PBS medium were collected and replaced with an equal volume of fresh PBS to maintain sink conditions. The amount of released PST-1/proPST-1 was quantified at each time point using UV-vis spectroscopy, and cumulative release was expressed as a percentage of the total amount of proPST-1 initially present in the proPST-1@PN formulation.

## Results and discussion

We started our research by performing molecular docking studies to comparatively assess the predicted binding modes of CA-4, PST-1 and proPST-1 within the colchicine binding pocket of tubulin (Fig. 1 and S1–S7). *Cis*-PST-1 showed a favourable docking score (−7.9 kcal mol^−1^), comparable to that of *cis*-CA-4 (−8.0 kcal mol^−1^), whereas the corresponding trans isomers showed less favourable scores of −6.0 and −5.9 kcal mol^−1^, respectively (Table S1a). The predicted binding pose of *cis*-PST-1 also retained key interactions within the colchicine-binding pocket, consistent with its higher experimentally observed biological potency. In contrast, ONB-caged PST-1 showed less favourable docking scores for both the cis (−5.2 kcal mol^−1^) and trans (−5.5 kcal mol^−1^) configurations (Table S1a and b), with predicted poses showing altered orientations and interactions within the colchicine-binding pocket compared with native PST-1 (Fig. 1 and S4). Independent docking runs showed good overall reproducibility of the predicted binding poses. Using an RMSD of ≤2.0 Å as a practical criterion for reproduction of a similar binding orientation, all replicate poses for *cis*-PST-1, *trans*-PST-1, and *trans*-proPST-1 remained within this criterion, whereas one alternative pose for *cis*-proPST-1 exceeded 2.0 Å (RMSD = 2.826 Å; Table S2).

**Fig. 1:**
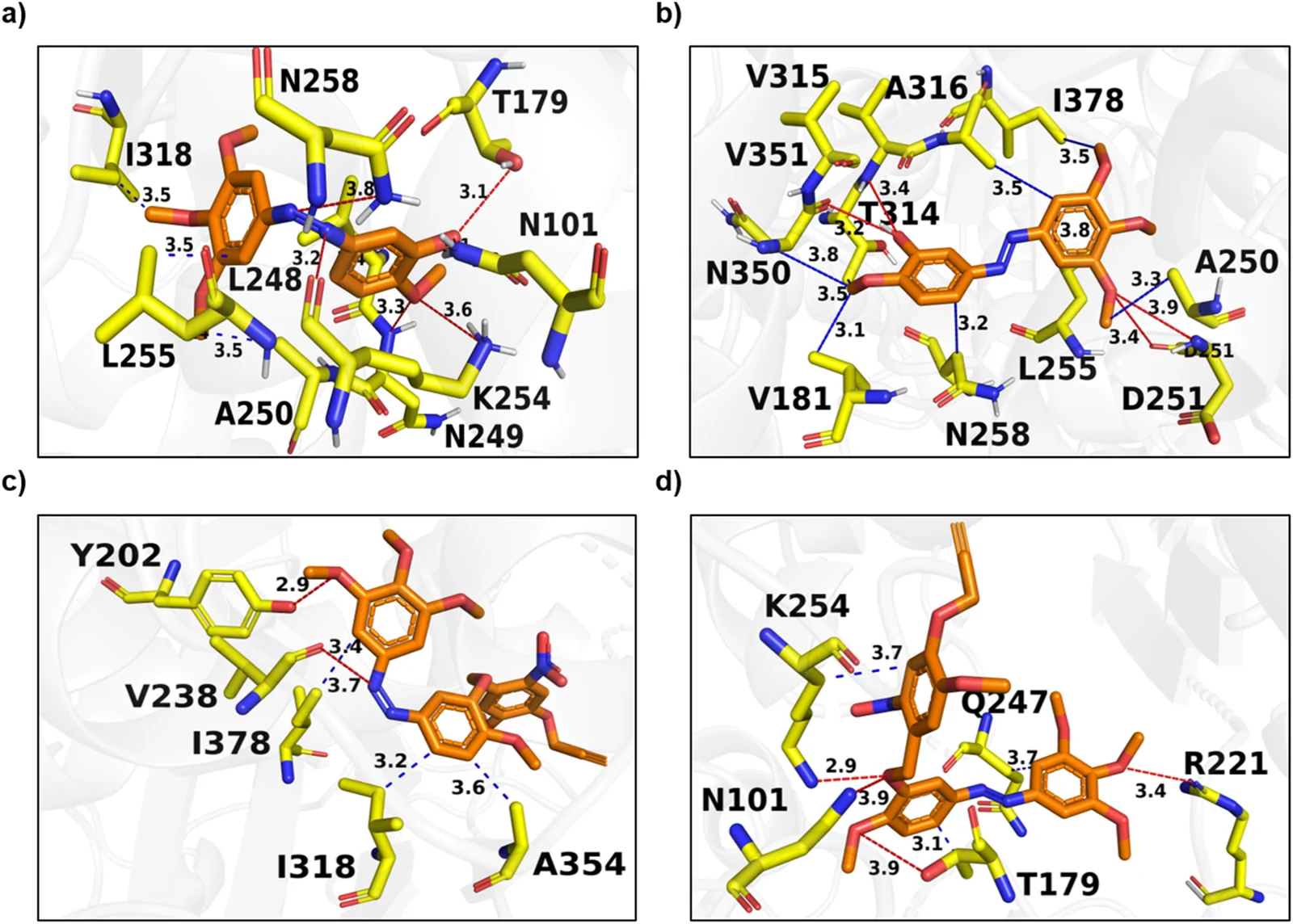
Representative lowest energy docking poses of (a) *cis*-PST-1, (b) *trans*-PST-1, (c) *cis*-proPST-1, and (d) *trans*-proPST-1 within the colchicine binding pocket of tubulin (PDB ID: 5LYJ). Ligands are represented as orange sticks, while interacting amino acid residues are shown as yellow sticks and labelled using single-letter amino acid notation. Hydrogen-bonding and hydrophobic interactions are indicated by red and blue dashed lines, respectively, with corresponding interaction distances (Å) shown. Structures and interaction maps were generated using PyMOL v3.0.

Additional docking using AutoDock 4.2 showed a similar result, supporting a qualitative interpretation of the docking results (Fig. S7 and Table S1a). Collectively, the docking results suggest that ONB caging may alter the predicted accommodation and interactions of PST-1 within the colchicine-binding pocket, providing a qualitative structural rationale for the experimentally observed reduction in biological activity of proPST-1 in the dark. Upon irradiation, photocleavage of the ONB group would restore the native PST-1 structure, enabling light-triggered activation of microtubule inhibition with enhanced temporal precision.

Having obtained theoretical results, we first synthesised PST-1 following a reported protocol.^46^ Photophysical characterisation confirmed that PST-1 exhibits fully reversible photoswitching behaviour at 37 °C. Upon irradiation at 365 nm, rapid trans-to-cis isomerisation was observed, manifested by a pronounced decrease in absorbance at 380 nm (Fig. S8 and S9). Reversion to the trans isomer occurred either thermally in the dark or photochemically under 565 nm illumination, demonstrating bidirectional optical control. Thermal relaxation studies further revealed temperature-dependent kinetics: at physiological temperature, cis-to-trans recovery was faster than at room temperature within the first 3 h, while near-complete restoration of the trans state was achieved after 12 h under both conditions (Fig. S9). Importantly, repeated photoswitching cycles conducted at 37 °C showed minimal photofatigue, with no significant loss in absorbance intensity or switching efficiency over multiple irradiation cycles, confirming excellent photostability and structural integrity of PST-1 (Fig. S10).

Following validation of the parent photoswitch, proPST-1 was synthesised *via* benzylic S_N_2 etherification of PST-1 with *o*-nitrobenzyl bromide (ONB-Br) (Scheme S1). Installation of the ONB group masks the phenolic hydroxyl group that is critical for binding to the colchicine site of tubulin, thereby suppressing biological activity in the dark. In addition, the propargyl substituent introduces additional hydrophobic character to the molecule and provides a versatile, click ready handle for potential post-functionalisation or conjugation strategies. This synthetic modification establishes proPST-1 as a photocaged, optically activatable derivative of PST-1 designed for precise light-controlled microtubule inhibition (Scheme S1).

The stability of proPST-1 was first evaluated in different media by monitoring the absorbance of the solution overnight, during which no detectable change in absorbance values was observed (Fig. S11). Subsequently, the photocleavage behaviour of proPST-1 was examined by LC-MS. Analysis of PST-1 under dark conditions showed a major chromatographic peak at approximately 4.0 min, corresponding to the trans isomer, together with a minor peak at around 3.4 min assigned to the cis isomer. Irradiation at 365 nm resulted in a clear redistribution of the isomeric population, with the cis isomer becoming the dominant species, consistent with photoisomerisation of PST-1 (Fig. S12a). The photocleavage behaviour of proPST-1 was then evaluated under identical conditions. In the absence of irradiation, proPST-1 displayed a single dominant peak at approximately 5.0 min, confirming its stability in the dark. Upon exposure to 365 nm light, this peak decreased significantly, accompanied by the appearance of a new peak corresponding to cis-PST-1. These results indicate successful photocleavage of the *o*-nitrobenzyl protecting group and release of the active photoswitchable drug (Fig. S12b).

Photolysis was further corroborated by ^1^H-NMR spectroscopy. Prior to irradiation, proPST-1 exhibited a characteristic benzylic proton resonance at approximately 5.3 ppm. Following light exposure, this signal disappeared completely, while a new resonance at around 12 ppm emerged, consistent with the formation of a benzaldehyde-derived photolysis by-product (Fig. S13). Collectively, these LC-MS and NMR studies unequivocally confirm that proPST-1 undergoes photolysis upon irradiation, resulting in the release of *cis*-PST-1 as the predominant active species. Short irradiation experiments further demonstrated that photoisomerisation and photocleavage occur simultaneously, generating mixtures of proPST-1 and PST-1 isomers during early stages of irradiation (Fig. S14).

In addition, the long-term stability of proPST-1 in ACN/H_2_O (1 : 1, v/v) was investigated over a period of one month using LC-MS analysis. No new peaks were observed, revealing that proPST-1 remained highly stable in the dark over an extended period of time (Fig. S15). Based on the above experiments we propose a mechanism of simultaneous photoisomerisation and photocleavage of proPST-1 to yield cis isomer of PST-1 (Fig. S16).

Next, we investigated photocleavage and activation of PST-1 using a cell-free tubulin polymerisation assay, a well-established method to quantify microtubule assembly.^46^ In this assay, the polymerisation of tubulin into microtubules is monitored by following the increase in optical density at 340 nm, which reflects the formation of ordered microtubule structures over time. This system enables direct evaluation of microtubule inhibition without cellular uptake or metabolic confounders, making it ideally suited to assess photoactivation and release behaviour. Free proPST-1 was first evaluated to confirm that ONB caging effectively suppresses tubulin inhibition in the absence of light. Under dark conditions, proPST-1 showed no detectable inhibition of tubulin polymerisation, exhibiting polymerisation kinetics indistinguishable from the untreated control. Direct comparison of free proPST-1 and free PST-1 under both dark and irradiated conditions further confirmed that, following light exposure, the inhibitory profiles of the uncaged proPST-1 closely matched those of native PST-1 (Fig. S17a). Further, upon irradiation with 365 nm light, proPST-1 induced pronounced, concentration-dependent inhibition of tubulin polymerisation (Fig. S17b), demonstrating photochemical uncaging and generation of the active inhibitor.

To further corroborate these findings, we performed fluorescence-based microtubule imaging assay. In agreement with the turbidity measurements, control samples and treatments with free proPST-1 under dark conditions displayed normal microtubule growth and network formation. In stark contrast, irradiation of proPST-1 sample resulted in a marked, concentration-dependent disruption of microtubule structures, consistent with potent tubulin inhibition (Fig. S18). These results indicate that photocleavage fully restores the bioactivity of PST-1.

Poor aqueous solubility remains a major limitation for the biomedical application of many small molecules, including PST-1 and proPST-1. Due to their limited solubility in water, all initial photophysical and chemical characterisation studies were conducted in organic solvents. To improve the aqueous solubility of proPST-1 and facilitate its biological evaluation, amphiphilic heterograft polymers functionalised with hydrophobic dodecyl and hydrophilic Jeffamine M-1000 grafts were employed as nanocarriers for the encapsulation of proPST-1 in aqueous media (Fig. 2a). These copolymers are well known to form compartmentalised polymeric nanoparticles (PNs) with worm-like morphologies in water.^52^ The resulting nanoscale structures have a hydrophobic interior and hydrophilic exterior, making them particularly suitable for the encapsulation of poorly water soluble small molecules (Fig. 2b).^17,18,33–35^ Moreover, previous studies have demonstrated that such polymeric nanoparticles are well tolerated by cells, supporting their use as biocompatible drug carriers.^13^ Encapsulation of proPST-1 was investigated at different polymer to prodrug molar ratios in order to optimise formulation stability. At a 1 : 10 molar ratio, clear and homogeneous dispersions were obtained, and this formulation exhibited the highest prodrug feed while retaining a high encapsulation (94.9 ± 5.1%) among the conditions tested. In contrast, increasing the prodrug content to polymer to prodrug ratios of 1 : 20 and 1 : 50 resulted in visibly non-homogeneous, turbid solutions, indicating overloading of the hydrophobic core and compromised nanoparticle integrity (Fig. 2c, S19 and Table S3).

**Fig. 2:**
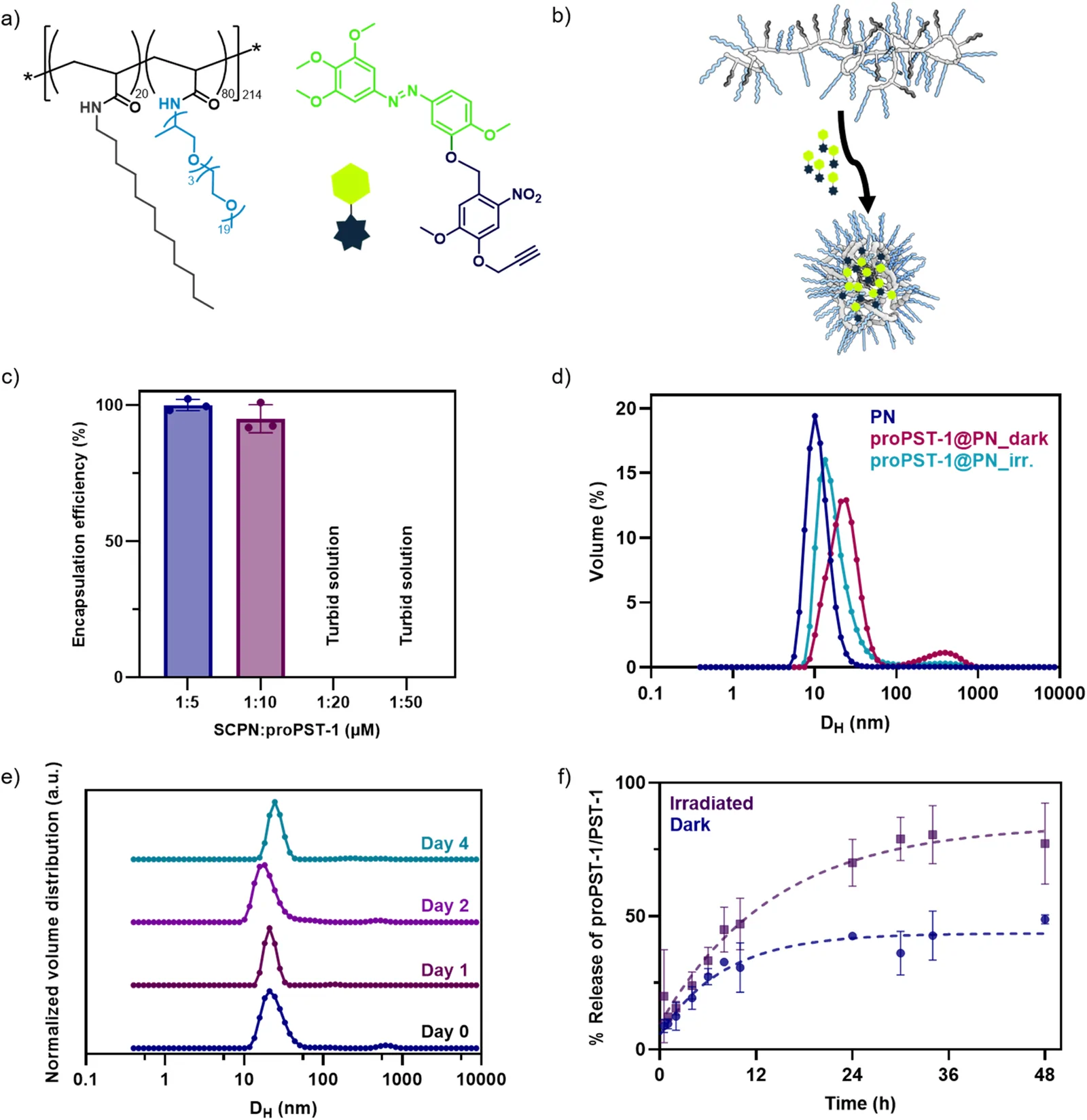
(a) Chemical structure of proPST-1 and the amphiphilic heterograft polymer used for nanoparticle formation. (b) Schematic illustration of the encapsulation of proPST-1 with a heterograft amphiphilic polymer and subsequent formation of polymer nanoparticles (PNs) through hydrophobic collapse in aqueous media. (c) Encapsulation efficiency of proPST-1 in PNs at different polymer to prodrug molar ratios. Homogeneous dispersions were obtained at lower loading ratios, while higher prodrug feed resulted in turbid solutions due to overloading of the hydrophobic core. (d) Dynamic light scattering (DLS) volume plot of unencapsulated PN, proPST-1@PN in the dark, and proPST-1@PN after irradiation (0.5 mg mL^−1^) with molar ratio of ([PN] : [proPST-1] = 1 : 10). Encapsulation of proPST-1 increased the particle size, consistent with cargo-induced swelling of the nanocarrier. Upon irradiation, the distribution shifted toward smaller diameters, suggesting photo-triggered de-loading and structural reorganisation of the nanoparticles (volume plot, *D*_H_ for PN = 10.1 nm, proPST-1@PN_dark = 24.4 nm, and proPST-1@PN_irr = 13.5 nm). (e) Dynamic light scattering (DLS) volume plot of proPST-1@PN in PBS over a period of 4 days (0.5 mg mL^−1^) with molar ratio of ([PN] : [proPST-1] = 1 : 10). The size distribution remained largely unchanged in dark, suggesting that proPST-1@PN remained stable under dark storage conditions at room temperature. (f) Release profile of proPST-1/PST-1 under dark (proPST-1) and irradiated (PST-1) conditions. Release of proPST-1/PST-1 was monitored over 48 h in PBS (pH 7.4) under dark and irradiated conditions. Irradiation resulted in a higher cumulative (∼80%) release compared with the dark (∼45%) condition. Data are presented as mean ± SD where applicable.

Dynamic light scattering (DLS) measurements were performed to assess the effect of proPST-1 encapsulation on nanoparticle size (Fig. S20 and S21). proPST-1 encapsulation in PNs led to an increase in the hydrodynamic diameter (*D*_H_), from 10.1 nm for the unencapsulated nanoparticles to 24.4 nm after loading, indicating that the polymers retain their nanoscale dimensions upon incorporation of the prodrug (Fig. 2d and S22). However, the increase in size also suggests that more than one polymer chain is present in the nanoparticles, which is why we refer to these binary systems as “amphiphilic polymeric nanoparticles (PNs)” rather than “single chain polymeric nanoparticles (SCPNs)”. Upon irradiation, the hydrodynamic diameter (*D*_H_) decreased toward values comparable to the unencapsulated PN (13.5 nm), suggesting photocleavage induced release of PST-1 accompanied by partial restructuring of the nanoparticles (Fig. 2d and S22).

To determine whether UV irradiation itself could induce disintegration of the PNs, the stability of empty PNs under irradiation conditions was investigated. No substantial change in the size was observed, indicating the PNs remained stable under UV irradiation (Fig. S23). Since the samples were filtered prior to DLS measurement, we evaluated whether hydrophobic filters affected the particle size and caused the loss of proPST-1 due to filtration. Although the empty PNs did not show any significant change due to filtration (Fig. S24a and b), a slight decrease in size of proPST-1@PN was observed post filtration (Fig. S24c and d) consistent with the loss of proPST-1 from proPST-1@PN after the filtration, as determined by absorbance measurements (Fig. S24e). Finally, we looked at the stability of proPST-1@PN in PBS over a period of four days. The size of the formulation remained largely unchanged suggesting good stability of proPST-1@PN under biologically relevant conditions (Fig. 2e and S25). Based on these results, a polymer to prodrug molar ratio of 1 : 10 was selected for all subsequent experiments.

Next, the release profile of proPST-1/PST-1 from the proPST-1@PN was evaluated in PBS (pH 7.4) under dark and irradiated conditions over a period of 48 h. In dark, a gradual release of proPST-1 was observed. However, irradiation enhanced the release, which can be attributed to the photocleavage of proPST-1 and formation of a more polar PST-1 (*cis*-) thereby facilitating the diffusion into the aqueous medium (Fig. 2f).

We next examined whether photoactivation is preserved when the prodrug is encapsulated within PNs using cell free tubulin polymerisation assay (Fig. 3a). Importantly, proPST-1@PN formulations showed no inhibition of tubulin polymerisation in the dark. Upon irradiation, however, a strong suppression of microtubule assembly was observed, comparable to that induced by free PST-1 and irradiated free proPST-1 (Fig. 3b and S26). This finding provides compelling evidence for light-induced uncaging followed by release of active PST-1 from the nanoparticle core into solution, where it can engage with tubulin.

**Fig. 3:**
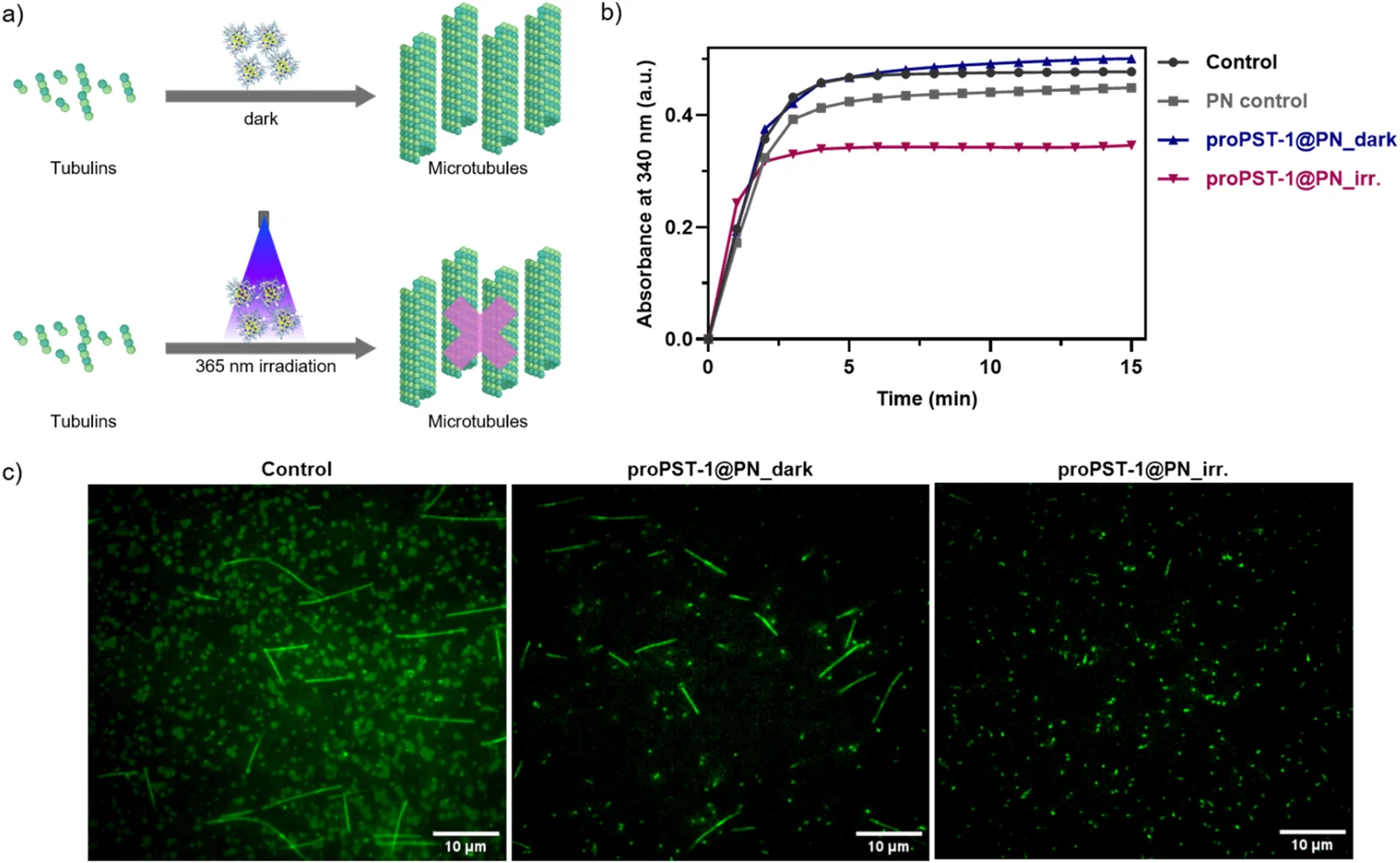
(a) Schematic representation of light-controlled inhibition of microtubule polymerisation by proPST-1-loaded polymer nanoparticles (proPST-1@PN). Under dark conditions, the encapsulated photocaged prodrug remains inactive, allowing normal microtubule formation. Upon 365 nm irradiation, photolytic uncaging and release of active *cis*-PST-1 suppress microtubule assembly. Created in BioRender. Singh, H. (2026) https://BioRender.com/fac5r0eT. (b) Tubulin polymerisation kinetics monitored by absorbance at 340 nm for control samples, empty polymer nanoparticles (PN control), and proPST-1@PN under dark and irradiated conditions. Irradiation of proPST-1@PN results in pronounced inhibition of tubulin polymerisation, whereas no inhibition is observed in the dark. (c) Fluorescence microscopy images of microtubule structures formed in the presence of control samples, proPST-1@PN under dark conditions, and irradiated proPST-1@PN. Normal microtubule growth and network formation are observed in control and dark conditions, while irradiation results in substantial disruption of microtubule assembly. Scale bars: 10 µm.

Further, we performed a fluorescence-based microtubule imaging assay. In agreement with the turbidity measurements, control samples and treatments with proPST-1@PN under dark conditions displayed normal microtubule growth while irradiation of encapsulated prodrug resulted in a marked, concentration-dependent disruption of microtubule structures, consistent with the results obtained from free proPST-1 (in DMSO) (Fig. 3c and S27). Together, these complementary assays confirm that the PN-encapsulated prodrug functions as a true light activated system, combining complete inactivity in the dark with photo-triggered release and restoration of microtubule inhibition.

To visualise the ability of proPST-1@PN to undergo light-triggered activation and release of *cis*-PST-1 in a cellular context, we evaluated its cytotoxicity in live HeLa and SH-SY5Y cells. As an initial control, we evaluated whether UV light alone, and empty PNs show any cytotoxicity. Cell control showed no toxicity due to irradiation alone (365 nm, 5.5 mW cm^−2^) and empty PNs were well tolerated by the cells at 1 mg mL^−1^ whereas at 2 mg mL^−1^ the PNs showed reduced viability in HeLa cells (Fig. S28a).

Next, the biological response to free proPST-1 (in DMSO) was examined. Under dark conditions, proPST-1 showed no detectable cytotoxicity across the tested concentration range, confirming that ONB caging effectively suppresses biological activity in the dark. Irradiation at 365 nm of proPST-1 induced a clear, concentration-dependent decrease in cell viability, consistent with photolytic uncaging and formation of the active microtubule inhibitor (Fig. 4a,b and S28b,c). We next assessed PNs loaded with proPST-1 to determine the effect of proPST-1@PN on cells. Importantly, cells treated with proPST-1@PN showed no cytotoxic effects in the dark. The results were in agreement with previous reports demonstrating the excellent biocompatibility and low intrinsic toxicity of these amphiphilic polymers.^13^ In contrast, cells treated with proPST-1@PN displayed a systematic, concentration-dependent cytotoxic response only upon irradiation. This behaviour confirms that light exposure triggers simultaneous uncaging and release of *cis*-PST-1 from proPST-1@PN within the cellular environment. The observed cytotoxicity profiles closely mirror those obtained for free proPST-1, indicating that encapsulation does not compromise drug efficacy after activation (Fig. 4c,d and S28b,c). Overall, these results demonstrate that proPST-1@PN functions as a biocompatible, light-activated nanocarrier system capable of precise spatiotemporal control over microtubule inhibition in living cells.

**Fig. 4:**
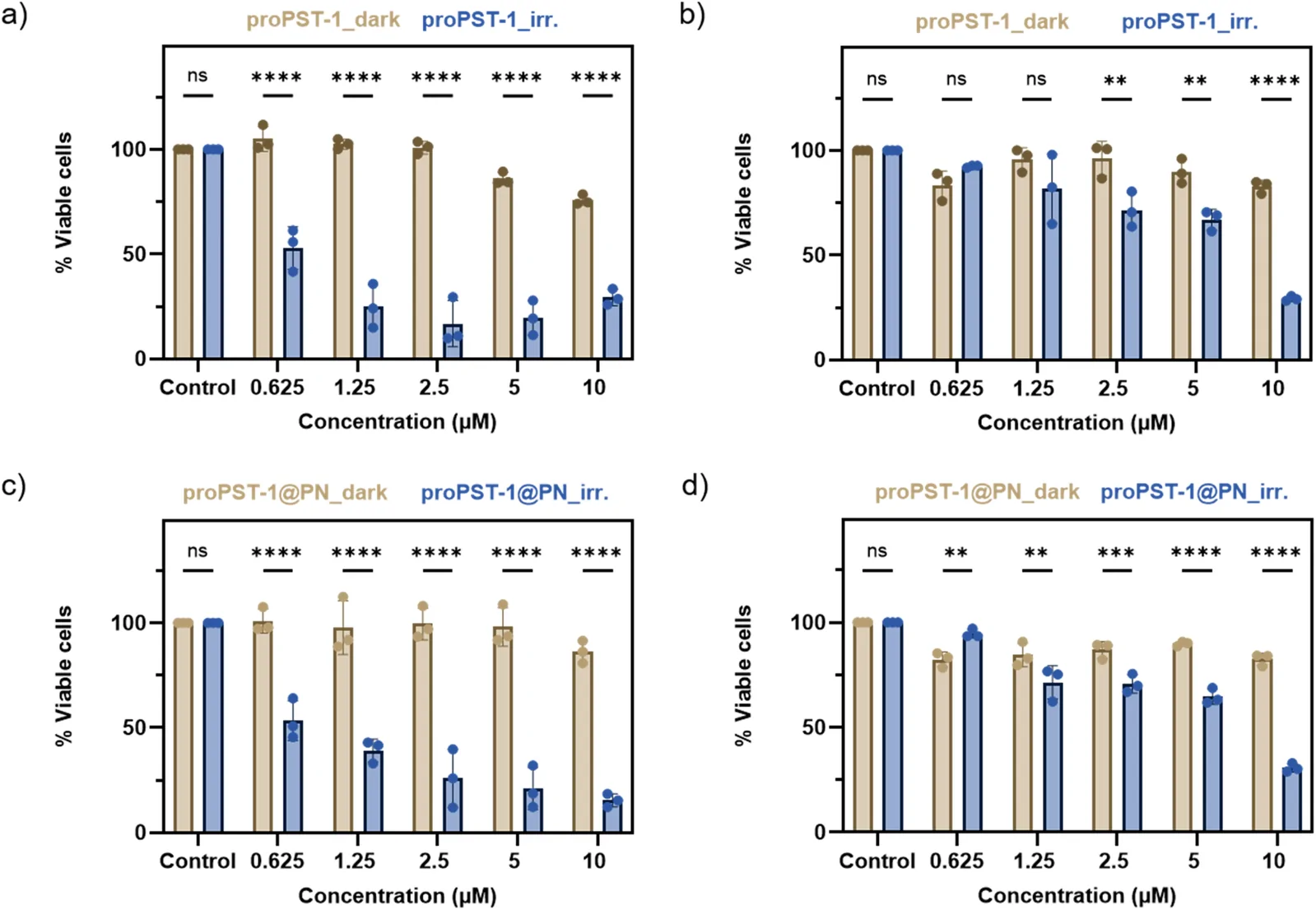
Cell viability following treatment with free proPST-1 under dark and irradiated conditions at different concentrations. In the absence of irradiation, proPST-1 shows minimal cytotoxicity, whereas 365 nm irradiation induces a pronounced concentration-dependent decrease in cell viability, consistent with photolytic uncaging and activation of PST-1 in (a) HeLa cells, (b) SH-SY5Y cells. Cell viability following treatment with proPST-1-loaded polymer nanoparticles (proPST-1@PN) under dark and irradiated conditions. Encapsulated proPST-1@PN remains non-toxic in the dark, while irradiation results in strong concentration-dependent cytotoxicity, confirming light-triggered activation and release of *cis*-PST-1 from the nanoparticle core in (c) HeLa cells, (d) SH-SY5Y cells. Cell viability was determined using the CCK-8 assay after treatment under the indicated conditions. Data are presented as mean ± SD. **P < 0.01, ***P < 0.001, ****P < 0.0001, and ns is not significant, assessed by two-way ANOVA followed by Šídák's multiple comparison test. In all cases, the concentration on the *X* axis refers to the (pro)PST-1 concentration.

## Conclusions

In summary, we report the rational design and synthesis of a photocaged prodrug of PST-1 that integrates theoretical insight with experimental validation to achieve simultaneous light-triggered activation and release of the active microtubule inhibitor. Guided by molecular modelling and microtubule polymerisation assays, installation of an *o*-nitrobenzyl (ONB) group on the phenolic hydroxyl of PST-1 suggested reduced tubulin binding and biological activity in the dark, while photocleavage upon irradiation regenerated active *cis*-PST-1. This strategy converts the intrinsically reversible photostatin system into an irreversible ON-switch, enabling strict spatiotemporal control over microtubule inhibition. To address the limited aqueous solubility of the prodrug, single-chain polymeric nanoparticles (SCPNs) were employed as delivery vehicles. These amphiphilic carriers provided excellent encapsulation efficiencies and maintained small, stable nanoparticle dimensions, albeit that the nanoparticles may comprise more than one polymer chain after encapsulation, likely a result of the hydrophobicity of proPST-1. Upon light exposure, uncaging and release of active, cis PST-1 from the PN core was observed, resulting in robust tubulin inhibition in cell-free assays and pronounced, light-dependent cytotoxicity in living cells. Together, our results establish amphiphilic heterograft polymers as highly effective and biocompatible carriers for light-gated drug delivery. Despite these advances, the limited tissue penetration of UV light remains a key challenge for *in vivo* and clinical translation. Future efforts will therefore focus on the development of photocaging and photoswitching groups that respond to longer wavelengths, ideally in the visible or near-infrared region. In addition, covalent attachment of the prodrug to the polymer will be evaluated although care has to be taken with the prodrug loading to avoid insolubility issues.^10^ The proof-of-concept study presented here provides a clear blueprint for the design of light-responsive macromolecular delivery systems. In addition to demonstrating precise optical control over drug activity, these findings define practical loading windows for SCPN carriers and offer general design principles for the development of next-generation, light-activated nanomedicines with improved translational potential.

## Author contributions

The manuscript was written with the contributions from all the authors. All the authors have given approval to the final version of the manuscript.

## Conflicts of interest

There are no conflicts to declare.

## Supplementary Material

NA-008-D6NA00763E-s001

## Data Availability

The data supporting this article have been included as part of the supplementary information (SI). Supplementary information is available. See DOI: https://doi.org/10.1039/d6na00763e.
