# Supplementary material for: Simultaneous activation and release of a microtubule inhibitor from amphiphilic polymeric nanoparticles
Source: Nanoscale Adv. 2026 Oct 7;8(22):6820–9. doi: 10.1039/d6na00763e (PMC13673699; doi:10.1039/d6na00763e)
Supplement: Supplementary file 1 [file NA-008-D6NA00763E-s001.pdf]

Supplementary Information (SI) for Nanoscale Advances.
This journal is © The Royal Society of Chemistry

Supplementary Information

Simultaneous activation and release of a microtubule inhibitor from amphiphilic polymeric nanoparticles

Harshit Singh,[a] Anand Kumar Thotakura,[b] A.P. Prakasham,[a] Esmee de Korver,[a] and Anja R.A. Palmans*[a]

[a] Department of Chemical Engineering & Chemistry and Institute for Complex Molecular Systems, Eindhoven University of Technology, P.O. Box 513, 5600 MB Eindhoven, The Netherlands. * Corresponding author, E-mail: a.palmans@tue.nl.

[b] The Russell H. Morgan Department of Radiology and Radiological Sciences, Johns Hopkins University School of Medicine, Baltimore, MD 21287, USA

Table of Contents

1 Experimental section S3
1.1 *In silico* molecular docking studies S3
1.2 Absorbance spectra of PST-1 in dark S9
1.3 Photostatin-1 (PST-1) photoswitching experiments S9
1.4 Photostatin-1 (PST-1) photoswitching integrity experiments S11
1.5 Synthesis of proPST-1 S11
1.6 Absorbance spectra of proPST-1 in dark S12
1.7 Photocleavage studies of proPST-1 by Liquid chromatography mass spectrometry (LC-MS) S13
1.8 Photocleavage studies of proPST-1 by ^{1}H-NMR S14
1.9 Effect of short exposure of light on proPST-1 S14
1.10 Long term stability of proPST-1 in dark S15
1.11 Proposed photocleavage mechanism of proPST-1 S17

1.12 *In vitro* tubulin polymerisation assay by UV-Vis spectroscopy (proPST-1) S17
1.13 *In vitro* tubulin polymerisation assay by fluorescence microscopy (proPST-1) S18
1.14 Encapsulation of proPST-1 in amphiphilic polymer S18
1.15 Dynamic light scattering (DLS) measurements S19
1.16 *In vitro* tubulin polymerisation assay by UV-Vis spectroscopy (proPST-1@PN) S24
1.17 *In vitro* tubulin polymerisation assay by fluorescence microscopy (proPST-1@PN) S25
1.18 Cell viability assay S26
2 Characterisation Data S27
3 References S32

1. Experimental Section

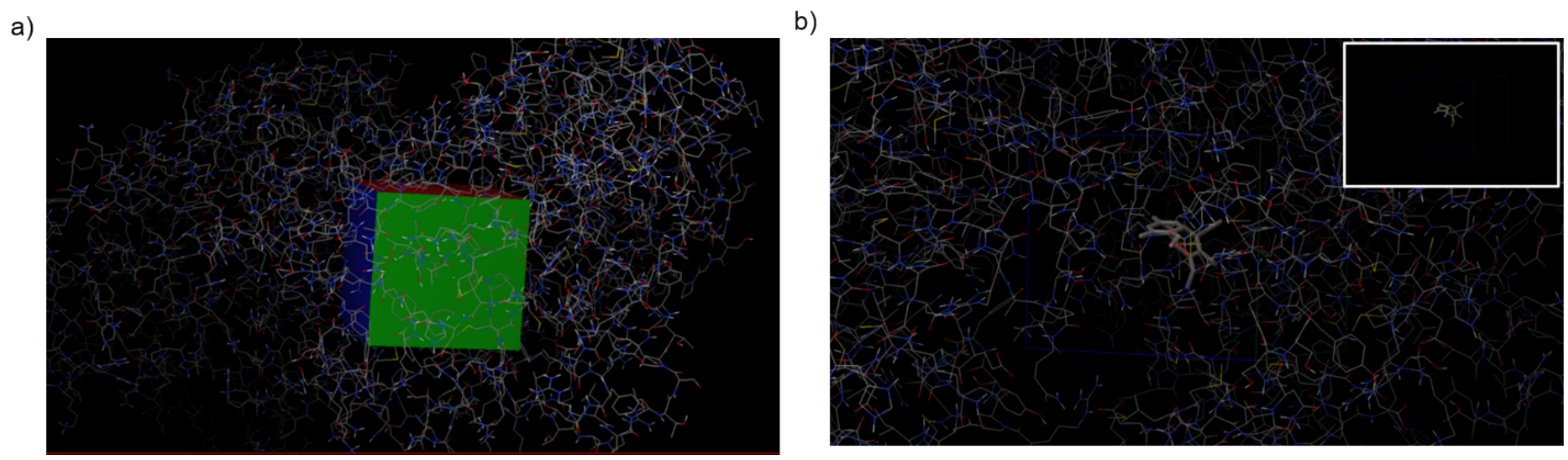

Figure S1. Screenshot illustrating the grid box used for docking the ligands at the binding site between the tubulin subunits (chains C and D and bound cis CA-4).

1.1. *In silico* molecular docking studies

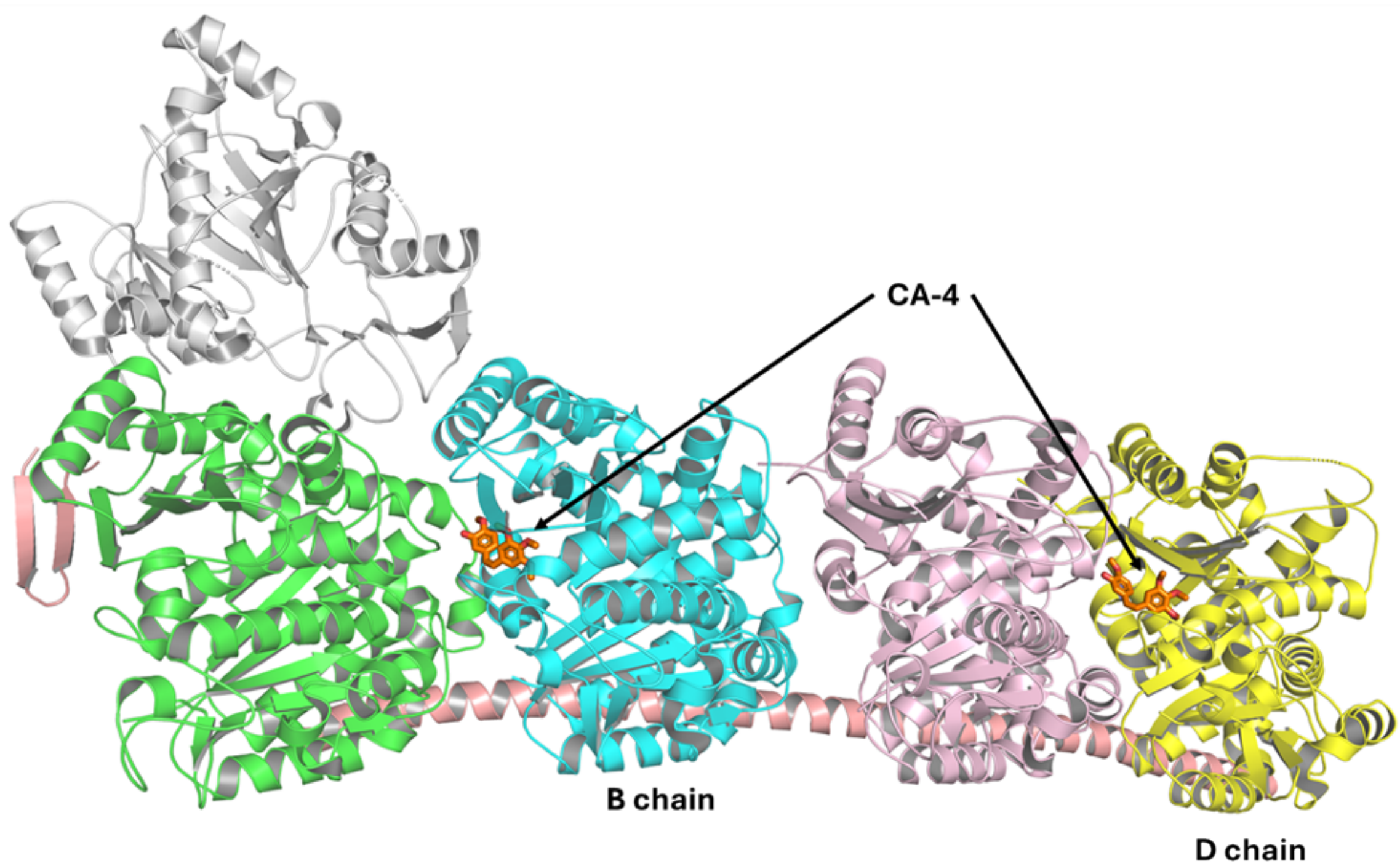

Figure S2. Molecular docking model of CA-4 bound at the colchicine-binding site of the tubulin (PDB: 5LYJ). The docking pose of CA-4 (orange sticks) is shown within the β-tubulin subunit (B chain, cyan/yellow), highlighting its interaction at the colchicine-binding pocket. The model illustrates the canonical binding orientation of CA-4 used as a reference for comparison with PST-1 and proPST-1 docking studies.

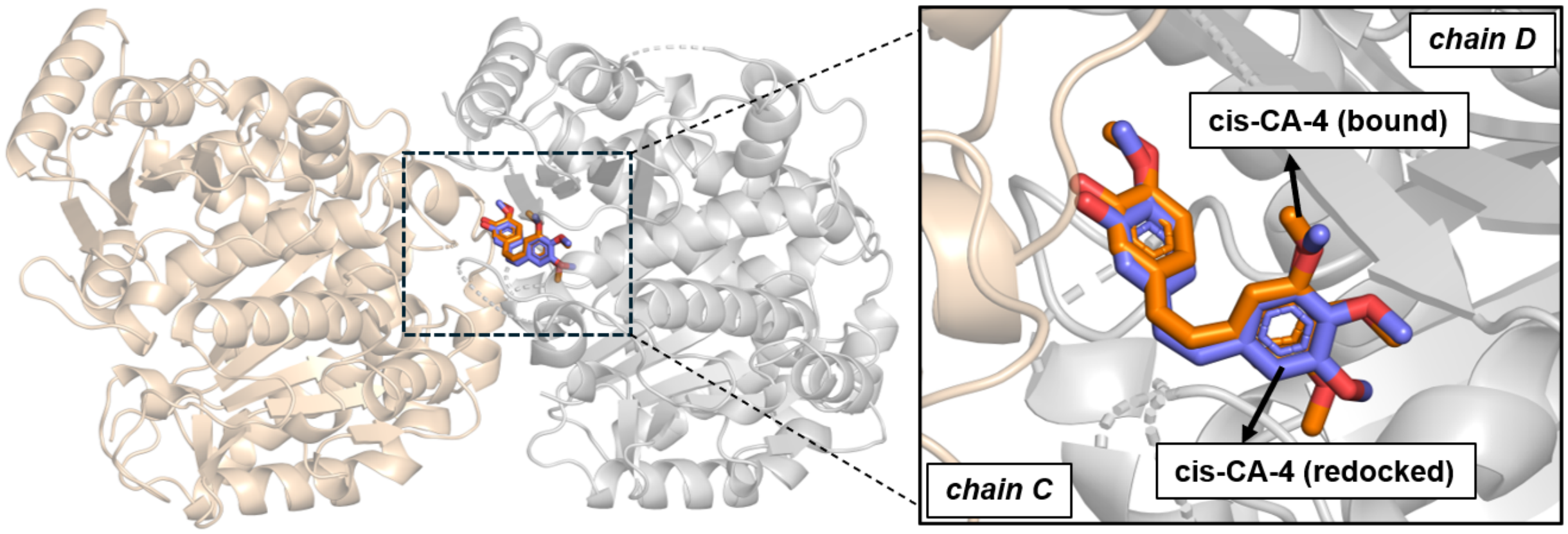

Figure S3. Zoomed-out (left) and zoomed-in (right) views of the colchicine-binding site of tubulin (chain D, gray ribbon; PDB ID: 5LYJ), showing the native bound cis-CA-4 (orange sticks) and the corresponding redocked pose (blue sticks). Superposition of the bound (orange) and redocked (violet) conformations of cis-CA-4 was performed using the ligand alignment tool in PyMOL. The resulting RMSD of 0.209 Å demonstrates close agreement between the experimentally determined and predicted binding poses.

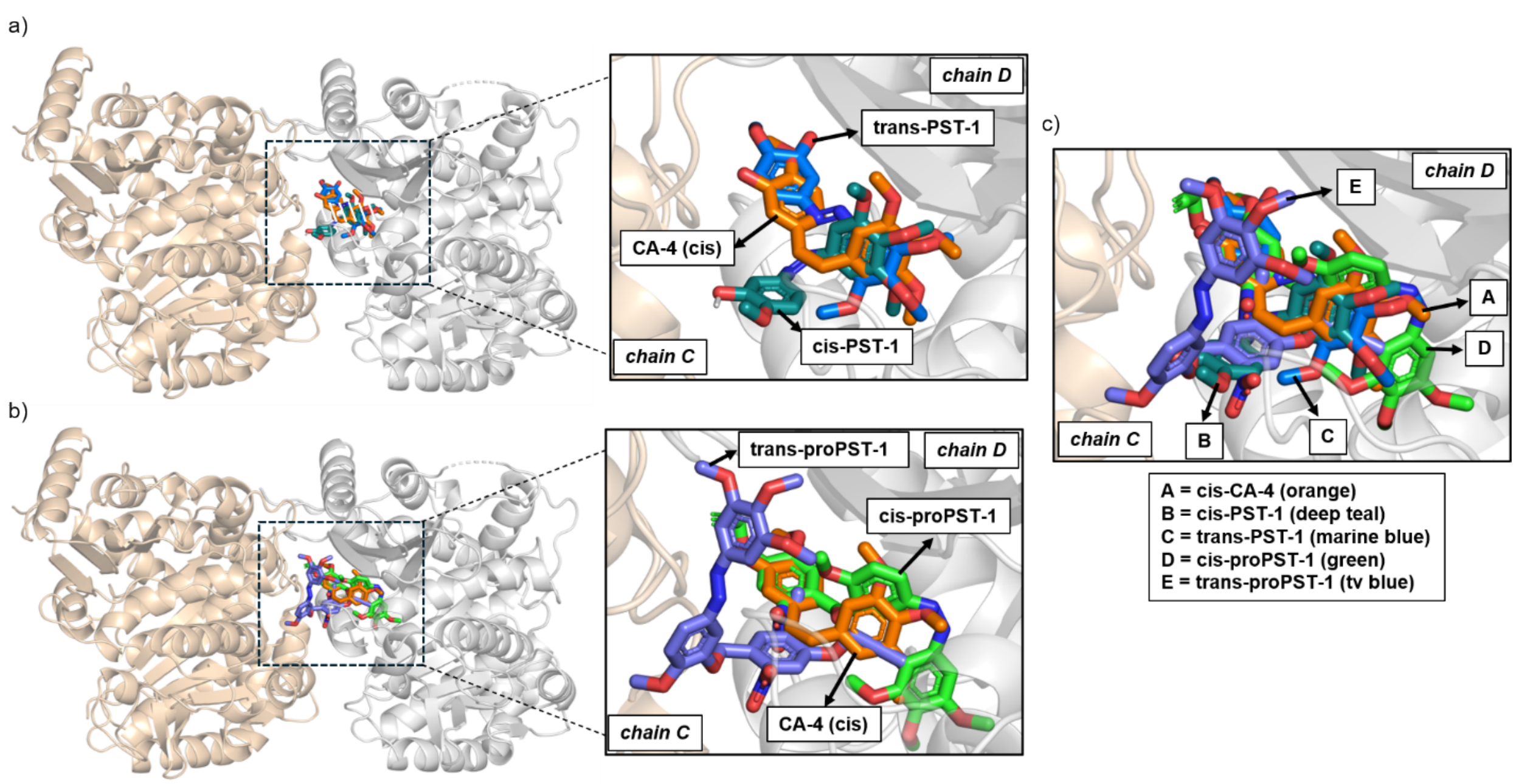

Figure S4. Comparative visualization of bound cis-CA-4 and the docked PST-1 and proPST-1 isomers within the colchicine-binding site of tubulin (PDB ID: 5LYJ). Zoomed-out (left) and zoomed-in (right) aligned views showing the native bound cis-CA-4 (orange) superimposed with (A) docked cis-PST-1 (deep teal) and trans-PST-1 (marine blue) and (B) docked cis-proPST-1 (green) and trans-proPST-1 (tv blue), and within the same binding pocket. (C) Composite aligned view of crystallographically bound cis-CA-4 together with all docked cis/trans PST-1 and cis/trans proPST-1 ligands superimposed within the common colchicine-binding site, enabling direct comparison of their predicted binding orientations. All ligands are shown as stick representations.

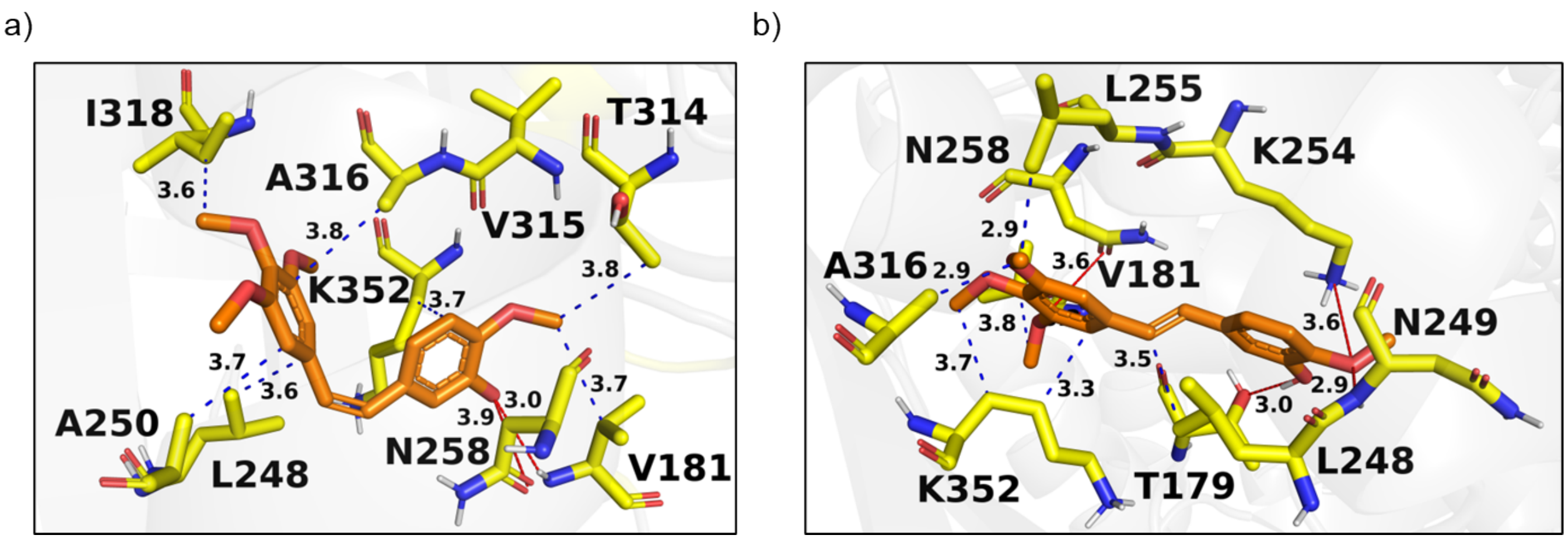

Figure S5. Cartoon representation of (a) bound ligand (CA4; cis form) and (b) trans-ligand CA4; trans form) into the active site of tubulin (PDB: 5LYJ). The docked ligands were shown as orange-colored sticks and the active site residues are shown as yellow-colored sticks and indicated by 1-letter code. The hydrogen and hydrophobic interactions are drawn as red and blue dotted lines, and the lengths are indicated. The figures were generated using PyMOL v 3.0.

Table S1a. Molecular docking scores (kcal/mol) of PST-1, and proPST-1 in their cis and trans configurations at the colchicine-binding site of tubulin obtained by Autodock Vina and AutoDock 4.2.

Compound code	Cis isomer<br>(docking score, kcal/mol)	Trans isomer<br>(docking score, kcal/mol)
Ligand (CA-4)	-8.0	-5.9
PST-1	-7.9 (Vina)	-6.0 (Vina)
	-7.6 (AutoDock 4.2)	-6.3 (AutoDock 4.2)
proPST-1	-5.2 (Vina)	-5.5 (Vina)
	-6.1 (AutoDock 4.2)	-4.3 (AutoDock 4.2)

Table **S1b.** Comparison of the reported docking parameters

	Docking parameters	Study
1.	Spacing = 0.400<br>Box = 43 × 50 × 42 (17.2 × 20 × 16.8 Å^3)<br>center: x = 13.375, y = 7.388, z = -22.645)<br>Autodock Vina	This study
2.	Spacing = 0.375	Panda and co-workers[1]

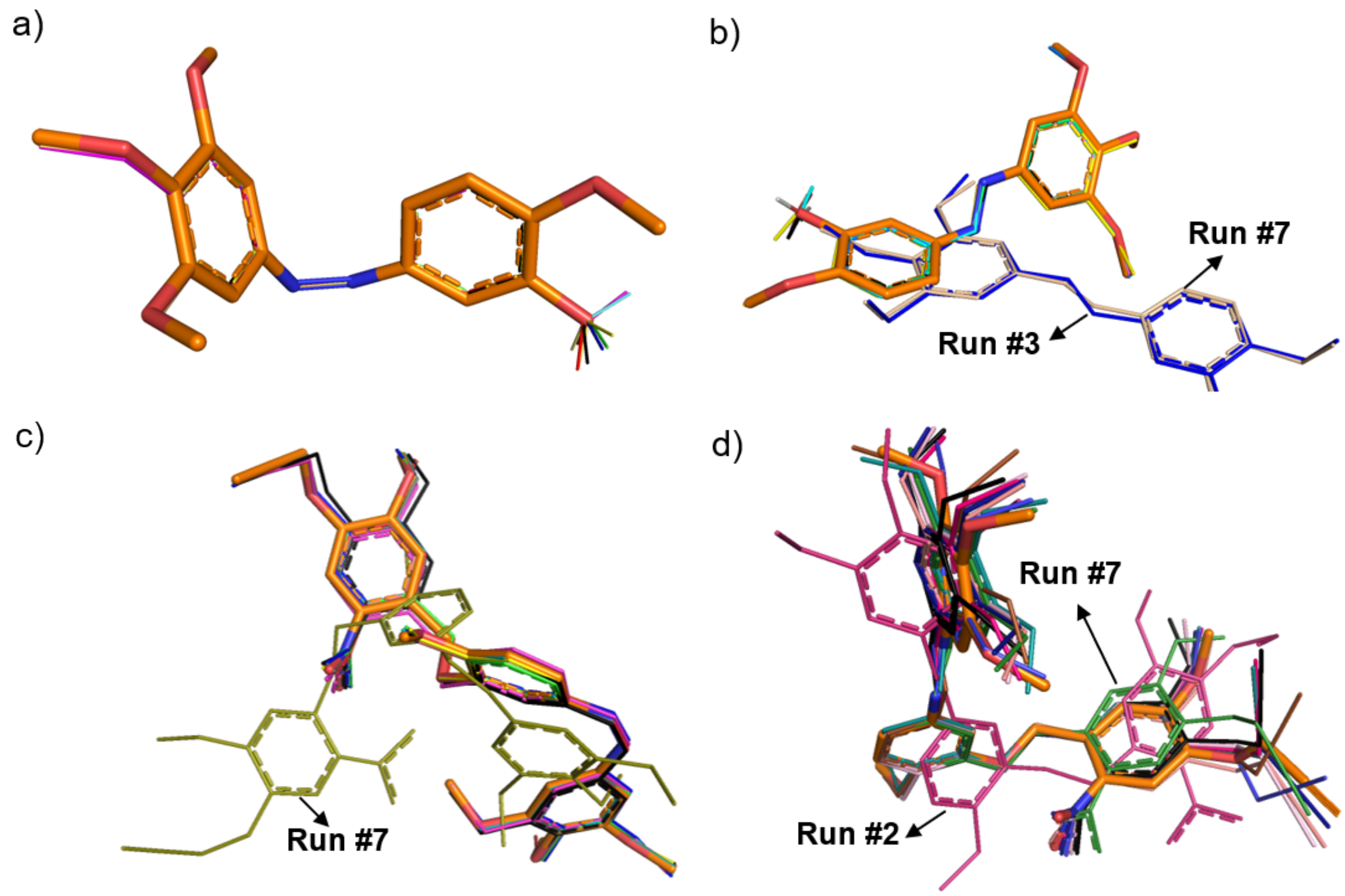

Figure S6. Convergence of the lowest-energy conformer from 10 independent docking runs, demonstrating reproducibility of the binding modes for (a) cis-PST-1, (b) trans-PST-1, (c) cis-proPST-1 and (d) trans-proPST-1. Superimposed poses are shown as coloured lines, with the representative conformer depicted in ball-and-stick representation. Docking convergence analysis demonstrating consistent lowest-energy conformers for (a) cis-PST-1 across 10 independent runs (100% convergence). In contrast, (c) cis- proPST-1 showed 90% convergence with variability in 1 out of 10 runs, whereas (b) trans-PST-1 and (d) trans- proPST-1 exhibited 80% convergence with deviations in 2 out of 10 runs yielding alternative conformations that are highlighted by arrows.

	Box = 56 × 60 × 58 (21 × 22.5 × 21.75 Å^3)<br>Autodock 4.2	
3.	Box = 40 × 40 × 40<br>Center: x = 118.026; y = 89.818; z = 6.327<br>AutoDockTools 1.5.6	Menezes and co-workers[2]

Table S2. Root mean square deviation (RMSD) analysis of the lowest energy docking conformers obtained from 10 independent docking runs for cis-PST-1, trans-PST-1, cis-proPST-1, and trans-proPST-1 at the colchine binding site of tubulin. The lowest energy conformer from run1 was used as the reference structure for comparison. Corresponding docking energies (-ΔG, kcal/mol) and RMSD values (Å) are reported for the reproducibility and conformational consistency of the predicted binding poses across independent docking stimulations.

Compound	Run1	ΔG, kcal/mol	Lowest energy conformers from 10 independent runs	ΔG, kcal/mol	RMSD, Å
**cis-PST-1**	conformer 1	-7.9	conformer 1 (run2)	-7.8	0.019
			conformer 1 (run3)	-7.9	0.014
			conformer 1 (run4)	-7.9	0.019
			conformer 1 (run5)	-7.9	0.015

			conformer 1 (run6)	-7.8	0.016
			conformer 1 (run7)	-7.9	0.025
			conformer 1 (run8)	-7.9	0.003
			conformer 1 (run9)	-7.9	0.008
			conformer 1 (run10)	-7.9	0.024
			conformer 1 (run11)	-7.8	0.01
**trans-PST-1**	conformer 1	-6	conformer 1 (run2)	-6.0	0.049
			conformer 1 (run3)	-5.7	0.803
			conformer 1 (run4)	-6.1	0.051
			conformer 1 (run5)	-5.9	0.042
			conformer 1 (run6)	-6.0	0.014
			conformer 1 (run7)	-5.9	0.84
			conformer 1 (run8)	-5.7	0.028
			conformer 1 (run9)	-6.0	0.08
			conformer 1 (run10)	-6.0	0.054
			conformer 1 (run11)	-6.1	0.044
**cis-proPST-1**	conformer 1	-5.2	conformer 1 (run2)	-5.4	0.093
			conformer 1 (run3)	-4.9	0.055
			conformer 1 (run4)	-4.9	0.159
			conformer 1 (run5)	-5.2	0.065
			conformer 1 (run6)	-5.1	0.093
			conformer 1 (run7)	-5.0	2.826
			conformer 1 (run8)	-5.2	0.084
			conformer 1 (run9)	-5.2	0.127
			conformer 1 (run10)	-4.9	0.084
			conformer 1 (run11)	-5.0	0.128
**trans-proPST-1**	conformer 1	-5.5	conformer 1 (run2)	-5.3	1.236
			conformer 1 (run3)	-5.6	0.093
			conformer 1 (run4)	-5.6	0.117
			conformer 1 (run5)	-5.6	0.156
			conformer 1 (run6)	-5.3	0.062
			conformer 1 (run7)	-5.6	0.59
			conformer 1 (run8)	-5.3	0.164
			conformer 1 (run9)	-5.6	0.147
			conformer 1 (run10)	-5.5	0.139
			conformer 1 (run11)	-5.4	0.174

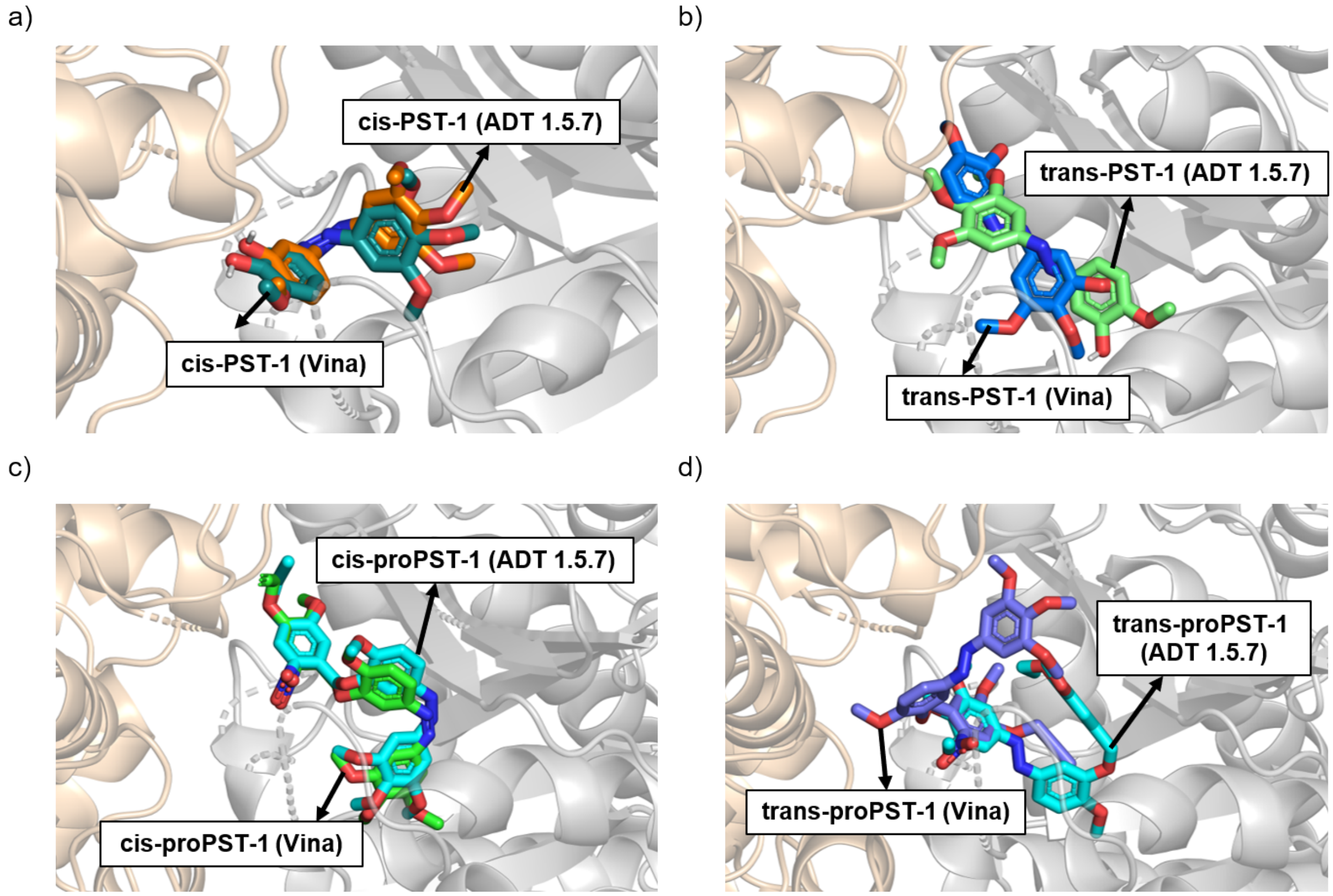

Figure S7. Comparative visualization of docked ligands (PST-1 and proPST-1 isomers) obtained by Autodock Vina and AutoDock 4 within the colchicine-binding site of tubulin (PDB ID: 5LYJ). Zoomed-in views showing the (A) docked cis-PST-1 (deep teal; Autodock Vina) superimposed with docked cis-PST-1 (orange; AutoDock 4), (B) docked trans-PST-1 (marine blue; Autodock Vina) superimposed with docked trans-PST-1 (green; AutoDock 4), (C) docked cis-proPST-1 (green; Autodock Vina) superimposed with docked cis-proPST-1 (cyan; AutoDock 4) and (D) docked trans-proPST-1 (tv blue; Autodock Vina) superimposed with docked trans-proPST-1 (cyan; AutoDock 4). All ligands are shown as stick representations.

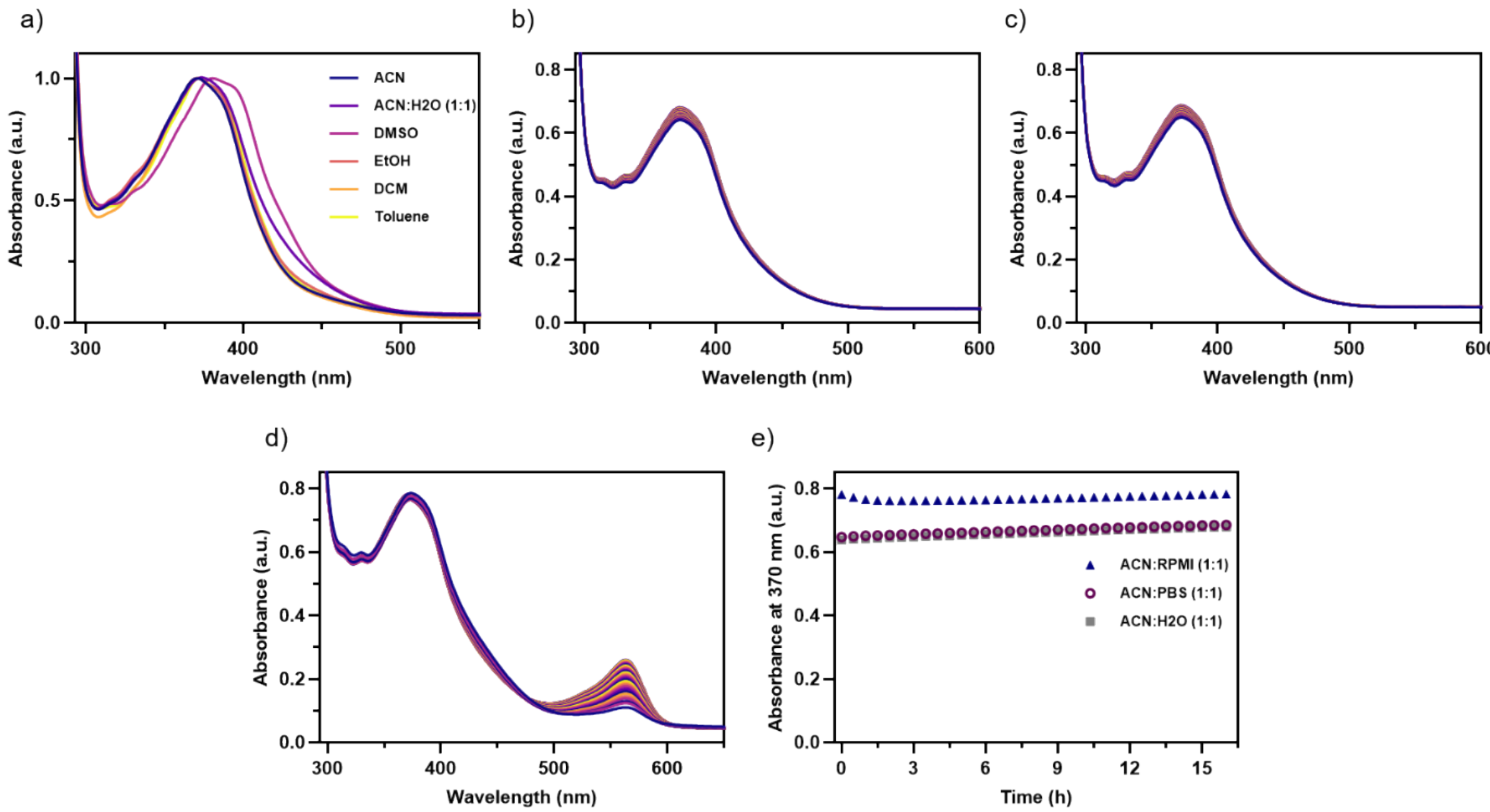

Figure S8. a) Normalised absorbance spectra of PST-1 (50 μM) in different solvents in dark. Time dependent absorbance spectra of PST-1 (50 μM) recorded over 16 h in the dark at room temperature in, **b)** acetonitrile/water (ACN/H_2O, 1/1, v/v), **c)** acetonitrile/PBS (pH=7.4) (ACN/PBS, 1/1, v/v), and **d)** acetonitrile/RPMI medium (ACN/RPMI, 1/1, v/v). **e)** Changes in absorbance at 370 nm over time in ACN/H_2O, ACN/PBS, and ACN/RPMI mixtures.

1.2 Absorbance spectra of PST-1 in dark

1.3 Photostatin-1 (PST-1) photoswitching experiments

For trans to cis isomerisation, a solution of PST-1 in DMSO (125 μL, 40 μM) in a black cuvette was irradiated with 365 nm light for 5 min and for the reverse photoswitching, samples were either left in dark or irradiated with 565 nm light.

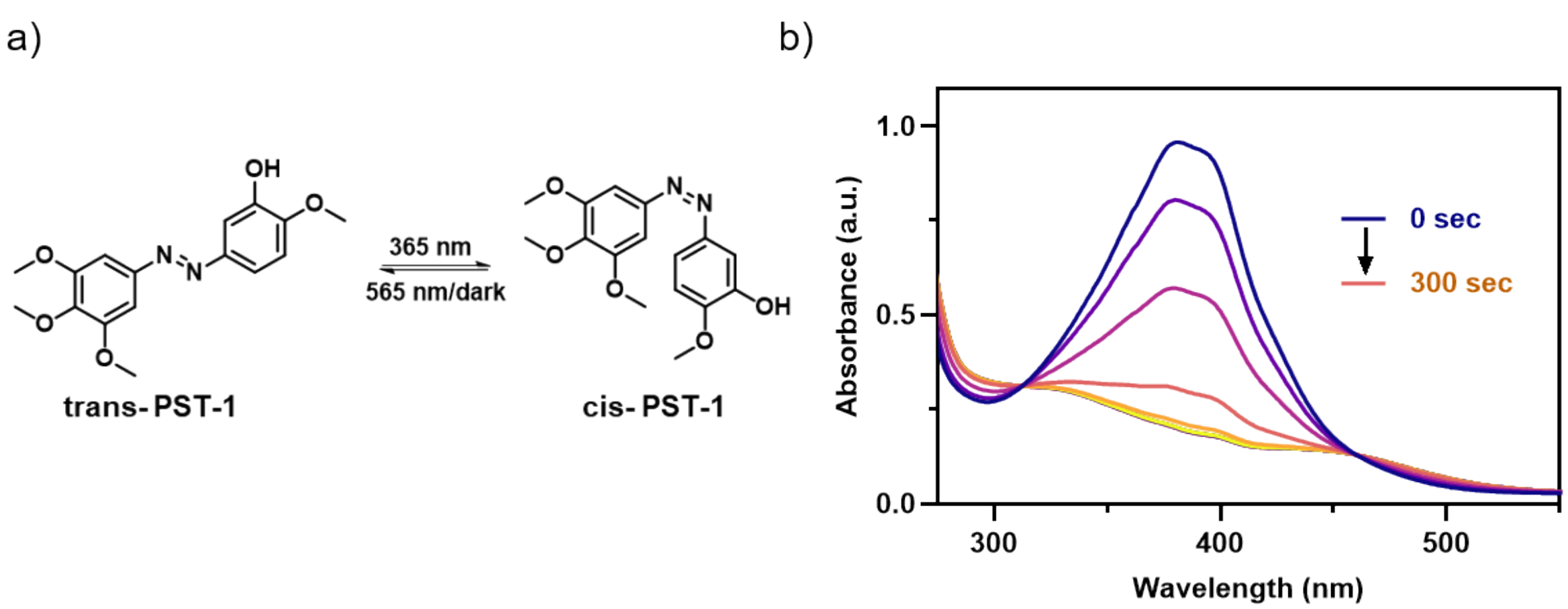
a)
b)
OH
N
N
365 nm
565 nm/dark
OH
trans- PST-1
cis- PST-1
0 sec
300 sec
Absorbance (a.u.)
Wavelength (nm)
1.0
0.5
0.0
300
400
500

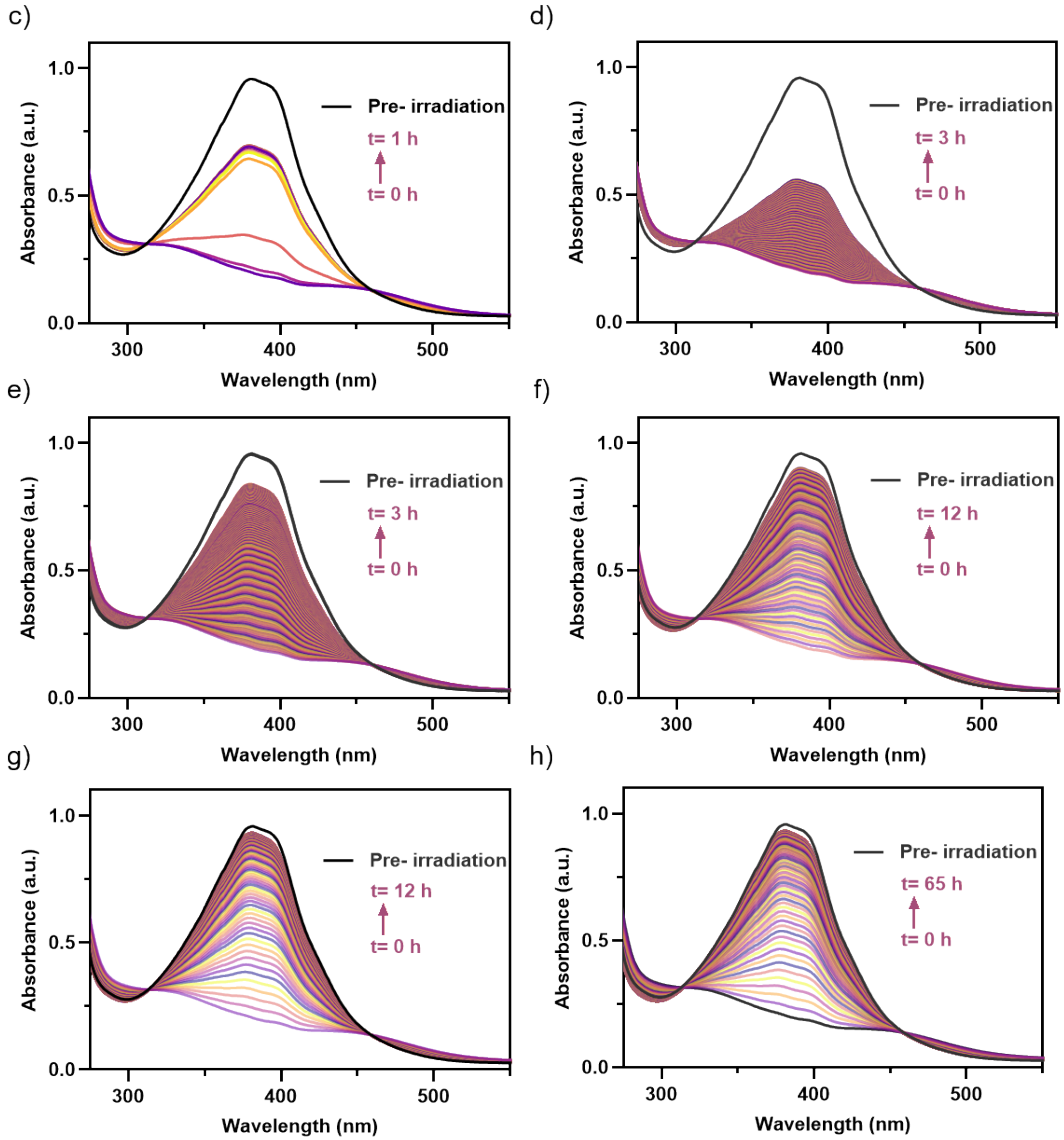

Figure S9. Reversible photoswitching and relaxation behaviour of PST-1 monitored by UV–Vis spectroscopy under different conditions. **a)** Photoisomerisation of PST-1, **b)** trans to cis isomerisation of PST-1 upon 365 nm irradiation at 37 °C for 5 min (40 μM in DMSO), **c)** Reverse photoswitching of PST-1 (from cis to trans) upon 565 nm irradiation at 37 °C (40 μM in DMSO), Thermal relaxation of PST-1 (40 μM in DMSO) in dark at, **d)** room temperature over a period of 3 h, **e)** 37 °C over a period of 3 h, **f)** room temperature over a period of 12 h, **g)** 37 °C over a period of 12 h, **h)** 37 °C over a period of 65 h; For panels **(c-h)**, samples were pre irradiated with 365 nm light for 5 min to generate cis isomer of PST-1 and then left in dark for thermal relaxation or irradiated with 565 nm light for reverse photoswitching.

1.4 Photostatin-1 (PST-1) photoswitching integrity experiments

A solution of PST-1 in DMSO (125 μL, 40 μM) at 37 °C in a black cuvette was irradiated with 365 nm LED lamp (5.5 mW/cm^2) for 1 min every 3 h and absorbance was recorded at 380 nm every 5 min for 27 h. Later, the solution was left in dark for another 38 h and final absorbance value was measured.

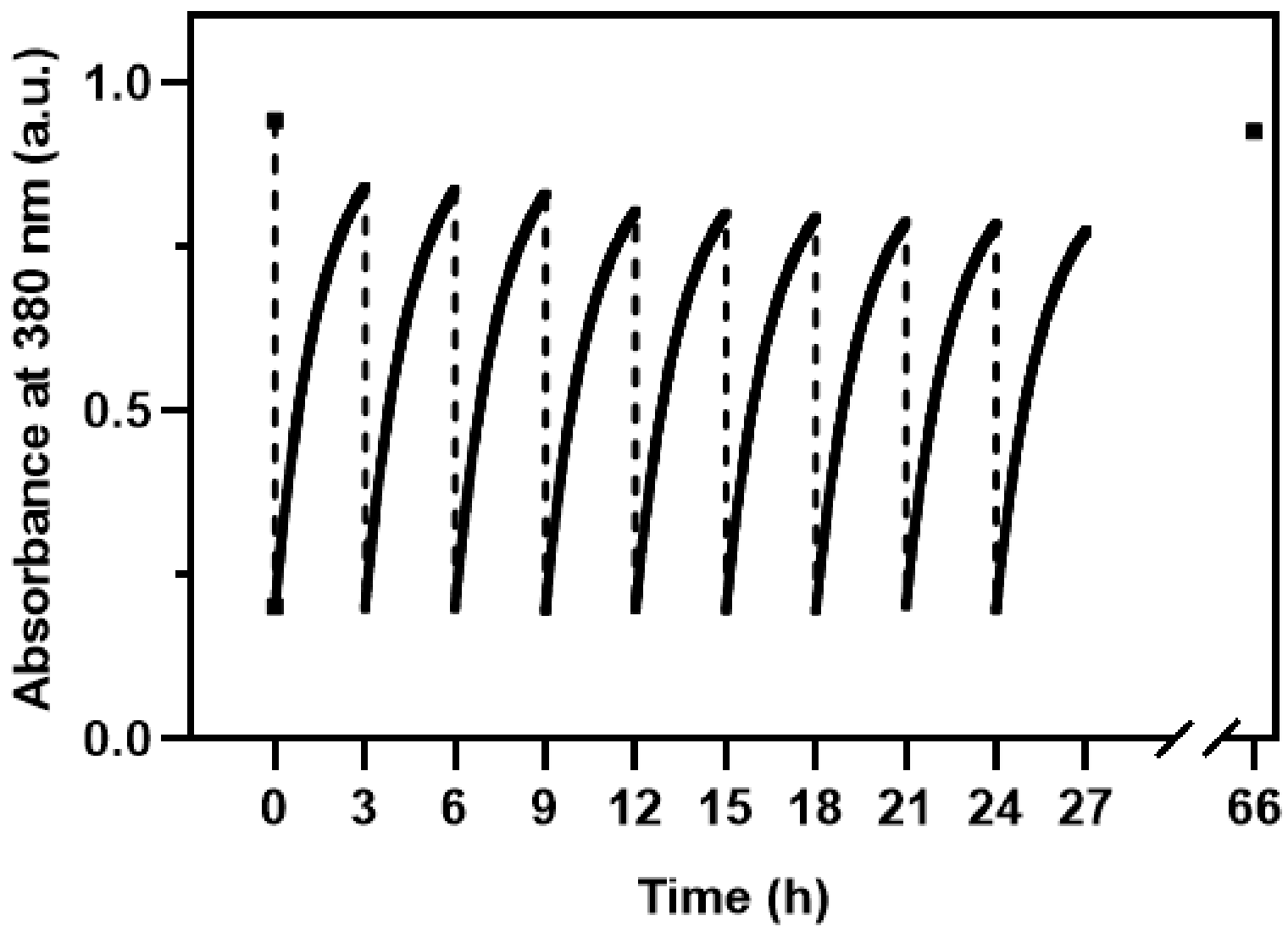

Figure S10. Photoswitching integrity and fatigue resistance of PST-1 under repeated irradiation cycles. Each irradiation cycle induced a reproducible decrease in absorbance followed by thermal relaxation in the dark, demonstrating reversible trans-cis photoisomerisation with minimal photofatigue over multiple cycles. After the final irradiation cycle, the sample was kept in the dark for an additional 38 h, resulting in recovery of the initial absorbance, indicating preservation of photoswitching integrity.

1.5 Synthesis of proPST-1

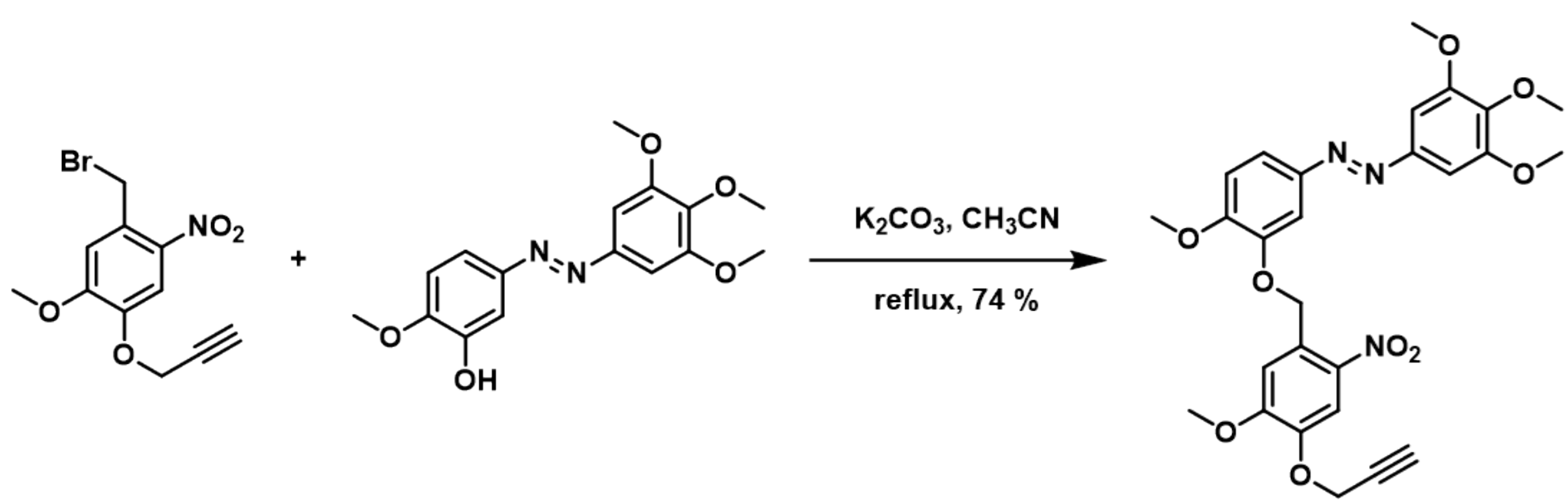

ONB-Br PST-1 proPST-1

Scheme S1. Scheme for proPST-1 synthesis.

1.6 Absorbance spectra of proPST-1 in dark

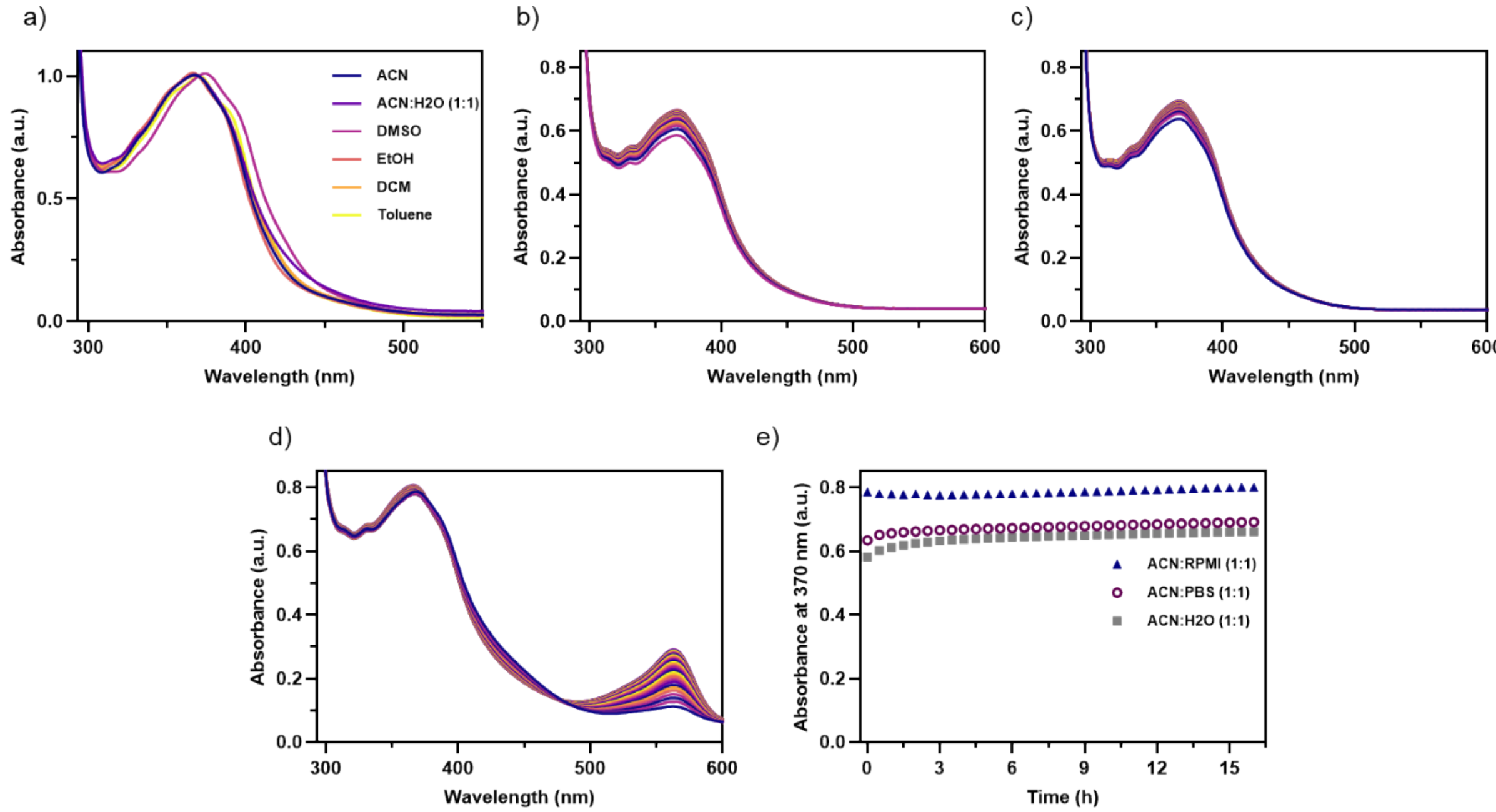

Figure S11. a) Normalised absorbance spectra of proPST-1 (50 μM) in different solvents in dark. Time dependent absorbance spectra of proPST-1 (50 μM) recorded over 16 h in the dark at room temperature in, **b)** acetonitrile/water (ACN/H_2O, 1/1, v/v), **c)** acetonitrile/PBS (pH=7.4) (ACN/PBS, 1/1, v/v), and **d)** acetonitrile/RPMI medium (ACN/RPMI, 1/1, v/v). **e)** Changes in absorbance at 370 nm over time in ACN/H_2O, ACN/PBS, and ACN/RPMI mixtures.

1.7 Photocleavage studies of proPST-1 by Liquid chromatography mass spectrometry (LC-MS)

Samples (trans-PST-1 and proPST-1) for the LC-MS were prepared in acetonitrile/water (ACN/H_2O, 1:1 v/v) at a concentration of 50 μM and protected from light by wrapping in aluminium foil. Later, 0.4 μL of each solution was injected in LC-MS to obtain retention time (R_t). The samples were then irradiated with 365 nm light (5.5 mW/cm^2) for 5 min in case of trans-PST-1 and 30 min for proPST-1 followed by a second LC-MS injection (0.4

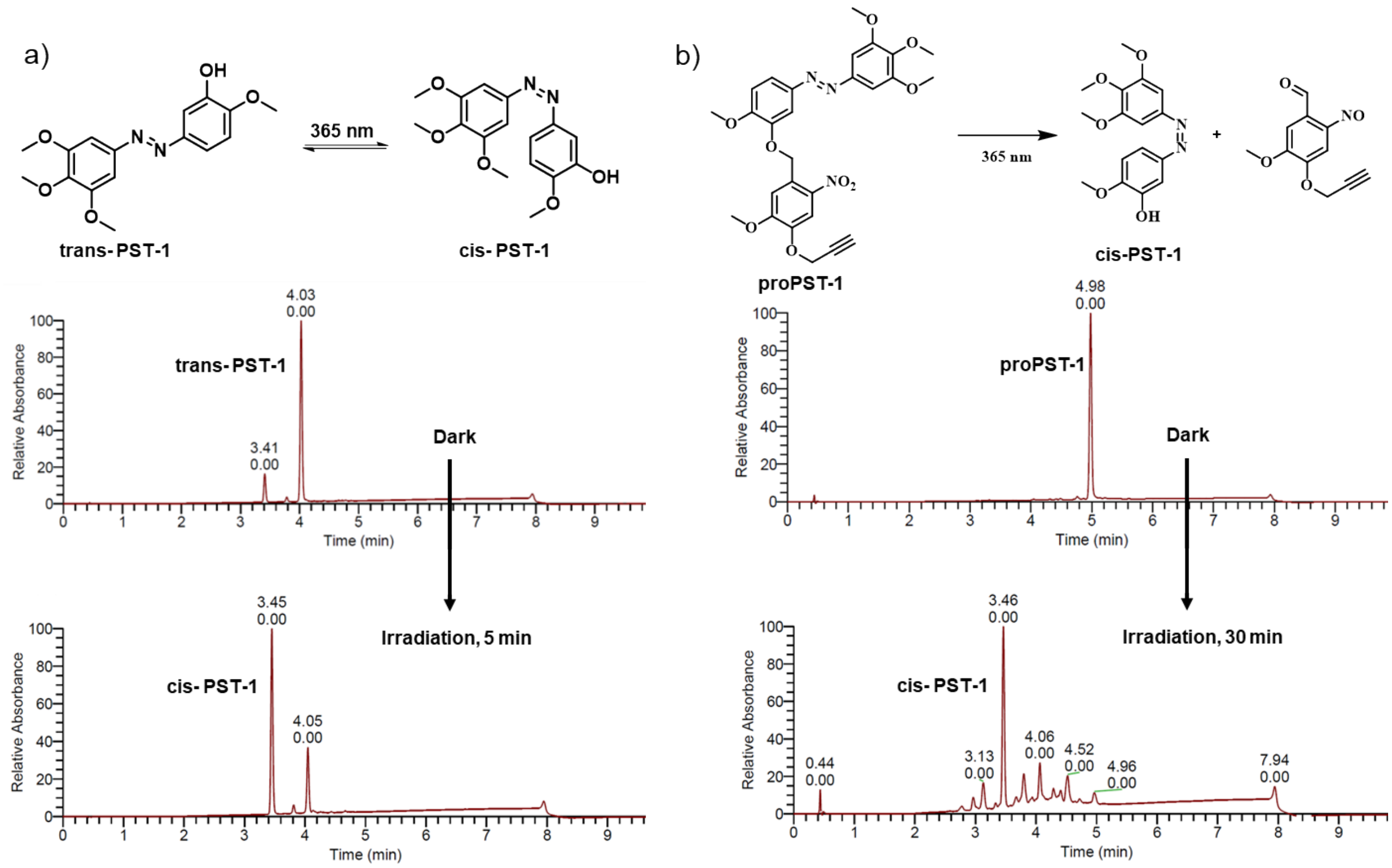

Figure S12. LC-MS chromatograms demonstrating the photoswitching and photocleavage behaviour of PST-1 (50 μM in ACN/H_2O) and proPST-1 (50 μM in ACN/H_2O). **a)** LC-MS chromatograms of PST-1 in the dark and after irradiation with 365 nm light for 5 min, showing isomerisation of trans-PST-1 ($R_t \sim 4.03$ min) to cis-PST-1 ($R_t \sim 3.45$ min), **b)** LC-MS chromatograms of proPST-1 in the dark and after irradiation with 365 nm light for 30 min. Irradiation resulted in disappearance of the proPST-1 peak ($R_t \sim 4.98$ min) and appearance of cis-PST-1 ($R_t \sim 3.46$ min), consistent with ONB photocleavage and release of PST-1 upon irradiation.

□L) for analysis.

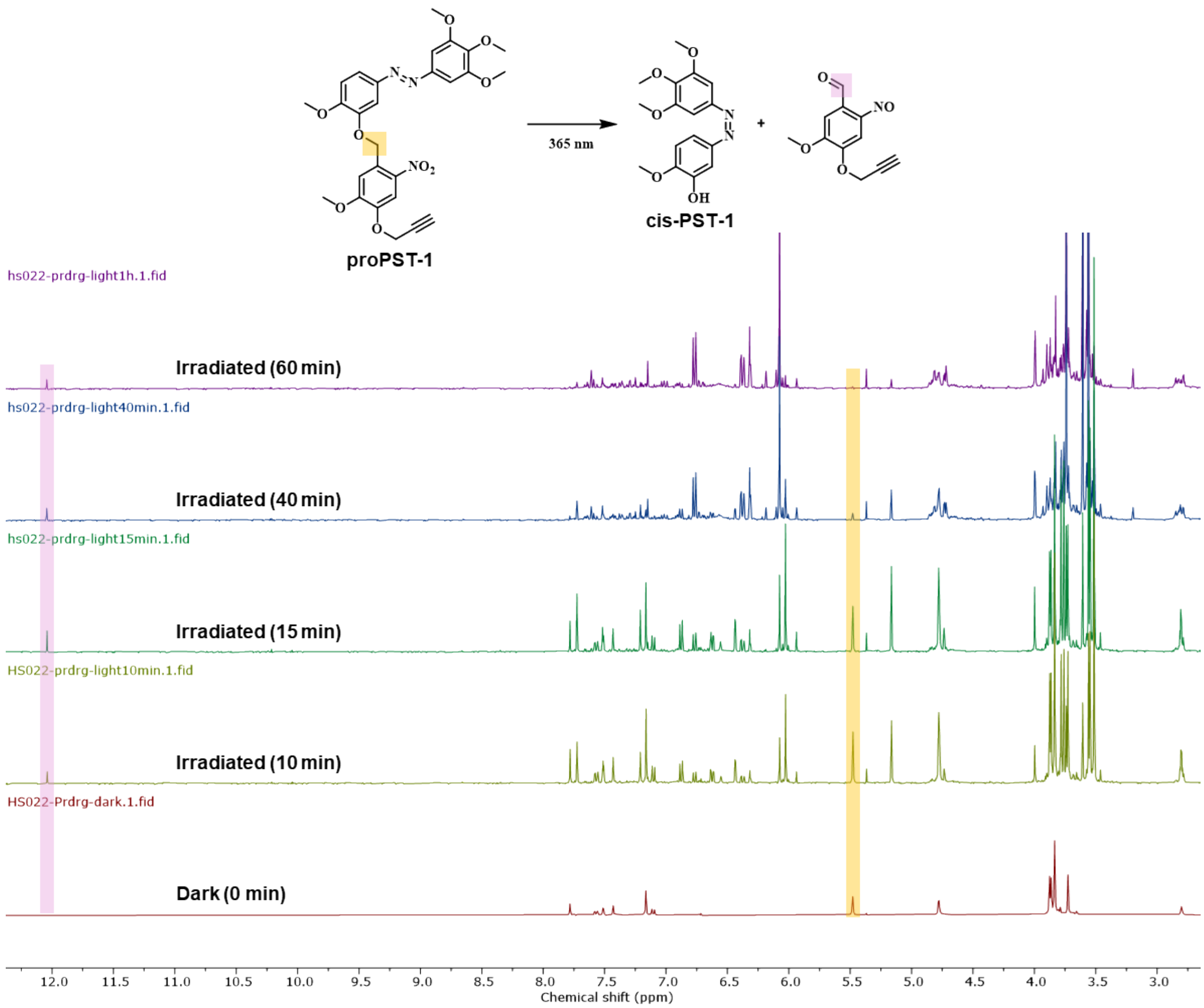

Figure S13. Photocleavage of proPST-1 monitored by ^{1}H-NMR spectroscopy upon irradiation of 365 nm light. ^{1}H-NMR spectra were recorded at different irradiation time points (0, 10, 15, 40, and 60 min). Progressive disappearance of the benzylic o-nitrobenzyl (ONB) proton signal at ~5.3 ppm (highlighted in yellow), together with the appearance of new aldehyde peak at ~12.0 ppm (highlighted in purple) corresponding to photocleavage products, confirms light-triggered photocleavage of proPST-1.

1.8 Photocleavage studies of proPST-1 by ^{1}H-NMR

A solution of proPST-1 in acetonitrile-d_3 (12 mM) in an NMR tube at room temperature was irradiated with 365 nm (5.5 mW/cm^2) and ^{1}H-NMR was recorded at different time points.

1.9 Effect of short exposure of light on proPST-1

A solution of proPST-1(50 μM) in acetonitrile/water (ACN/H_2O, 1:1 v/v) was injected (1.0 μL) and analysed by LC-MS prior to irradiation. The solution was then irradiated with 365 nm light (5.5 mW/cm^2) for 10 to 60 s followed by injection (1.0 μL) in LC-MS for analysis.

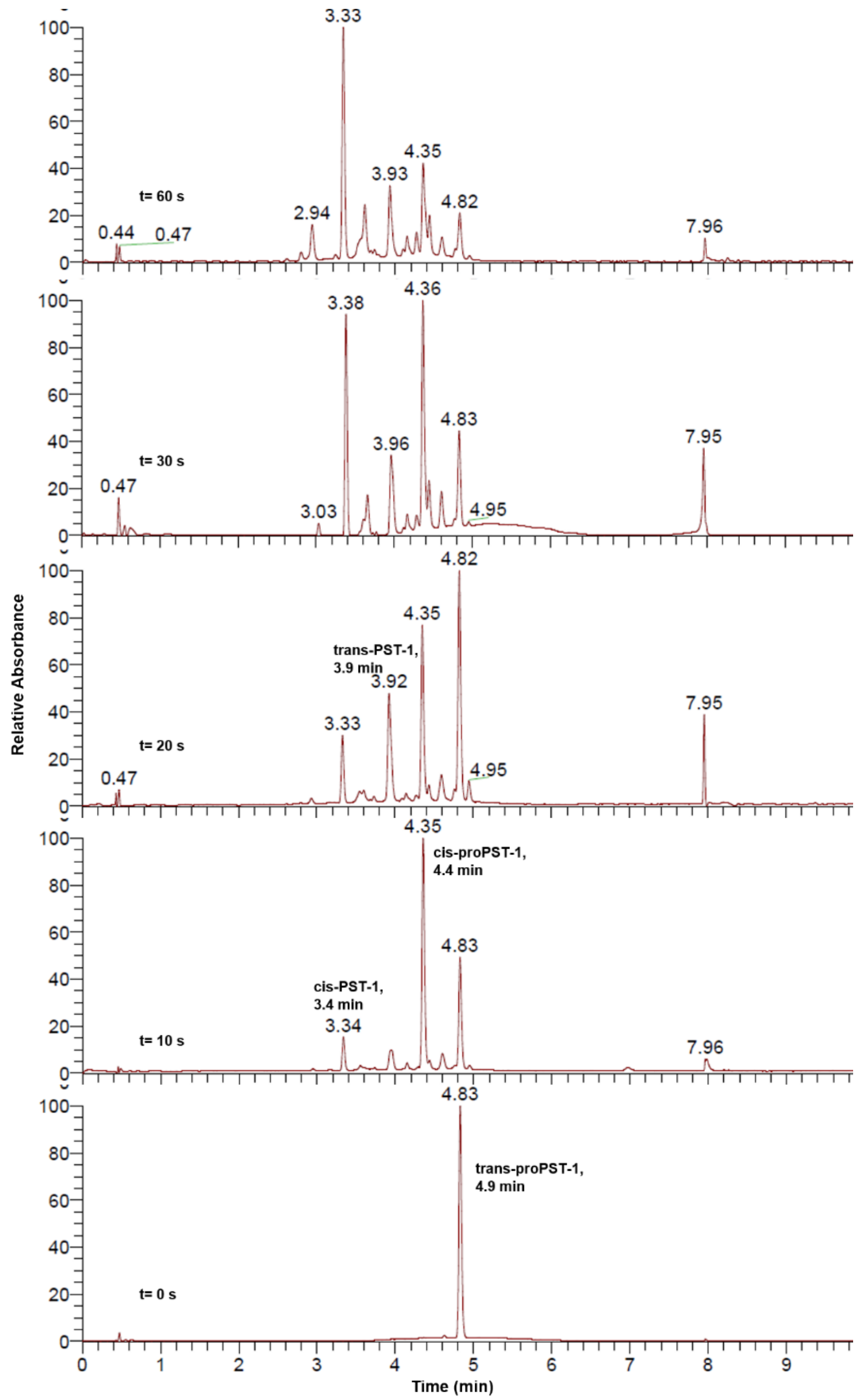

Figure S14. Short irradiation of proPST-1 indicates that photoisomerisation and photocleavage occur simultaneously. After 10-60 s irradiation, multiple LC-MS peaks are observed, consistent with the coexistence of trans/cis proPST-1 and trans/cis PST-1 species. The peaks at R_t ~4.9 (trans-) and ~4.4 (cis-) min can be assigned to proPST-1 isomers due to photoisomerisation, while the peaks at R_t ~4.0 (trans-) and ~3.4 (cis-) min correspond to PST-1 isomers generated after photocleavage. The simultaneous decrease of the parent proPST-1 peak and emergence of PST-1 peaks supports a coupled process in which azobenzene isomerisation and ONB uncaging take place under the same irradiation conditions.

1.10 Long term stability of proPST-1 in dark

Long term stability of proPST-1 (50 μM) in acetonitrile/water (ACN/H_2O, 1:1 v/v) was evaluated over a period of one month by LC-MS analysis using a 1.0 μL injection volume at different time points. The solution was stored at room temperature and wrapped with aluminium foil to prevent light exposure throughout the study.

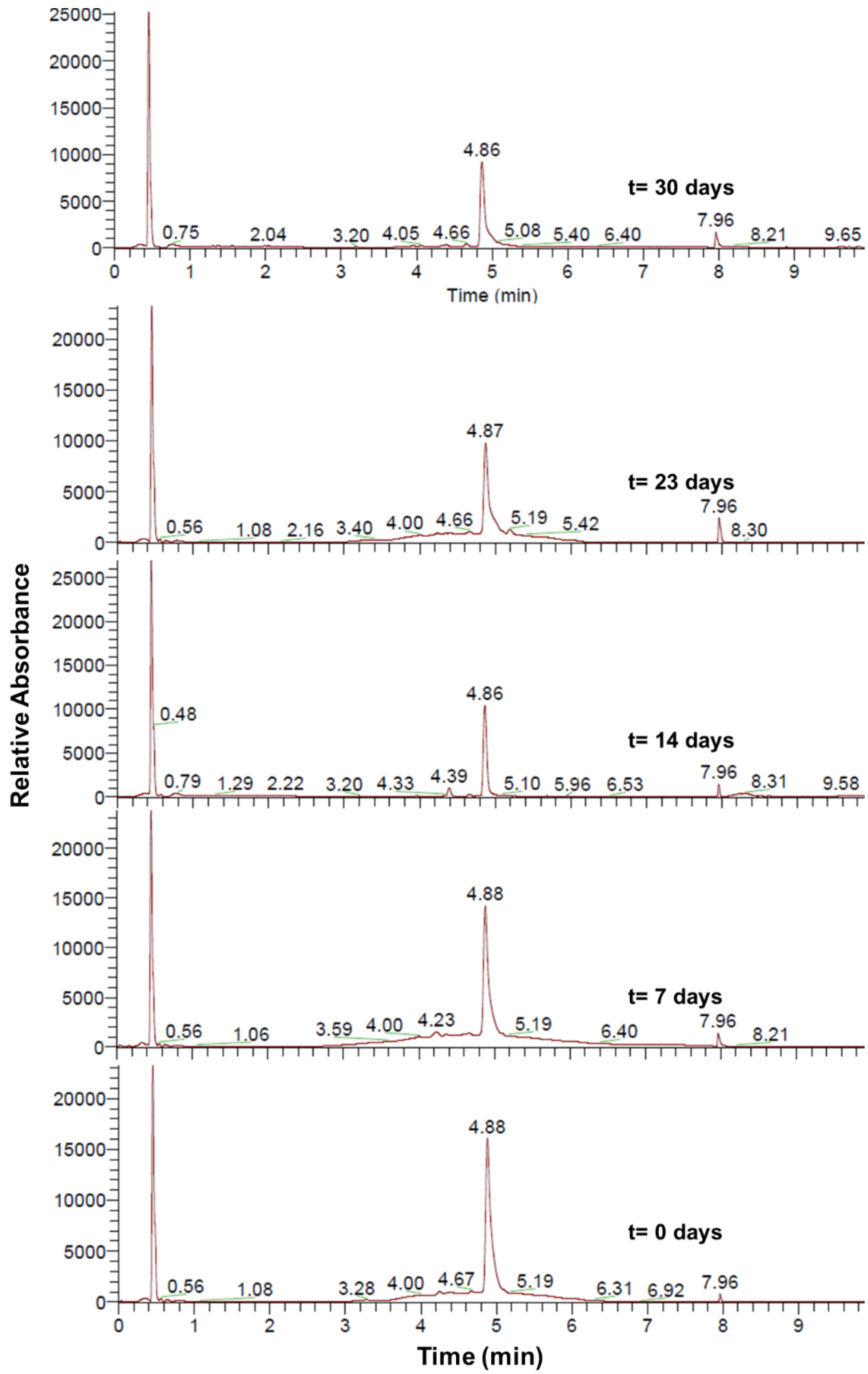

Figure S15. Long term stability of proPST-1 in the dark at room temperature was monitored by LC-MS over a period of 30 days. proPST-1 (50 μM) in acetonitrile/water (ACN/H_2O, 1:1, v/v) was stored in dark and injected (1.0 μL) at different time points. The chromatograms show that the characteristic peak at $R_t \sim 4.9$ min remained largely unchanged, throughout the study indicating good stability of proPST-1 under the storage condition.

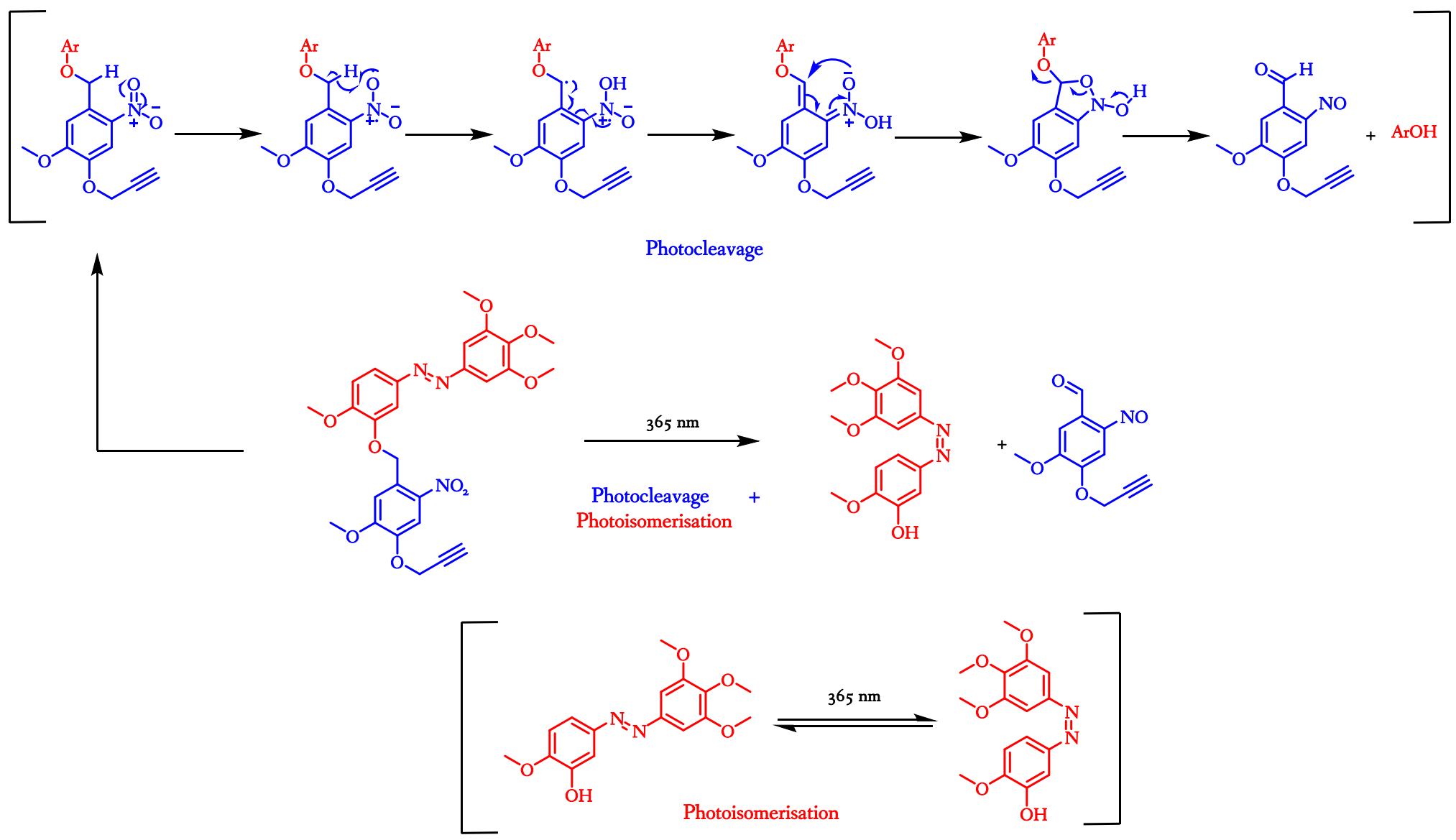

Figure S16. Proposed photocleavage and photoswitching mechanism of proPST-1 upon irradiation.

1.11 Proposed photocleavage mechanism of proPST-1

1.12 *In vitro* tubulin polymerisation assay by UV-Vis spectroscopy (proPST-1)

Tubulin MAP-rich (porcine brain) (Cat. #ML116), General tubulin buffer (Cat. #BST01), and GTP (Cat. #BTS06) were obtained from Cytoskeleton, Inc. Tubulin polymerisation reaction was performed at 37 °C in an Agilent Cary 3500 multicell UV-Vis spectrophotometer using 1 cm pathlength quartz cuvettes following a previously reported protocol.[3,4] Tubulin was first incubated for 10 min at 37 °C with either of PST-1 (in 1% DMSO) or proPST-1 (in 1% DMSO) in general tubulin buffer without GTP. Later, GTP (1 mM final

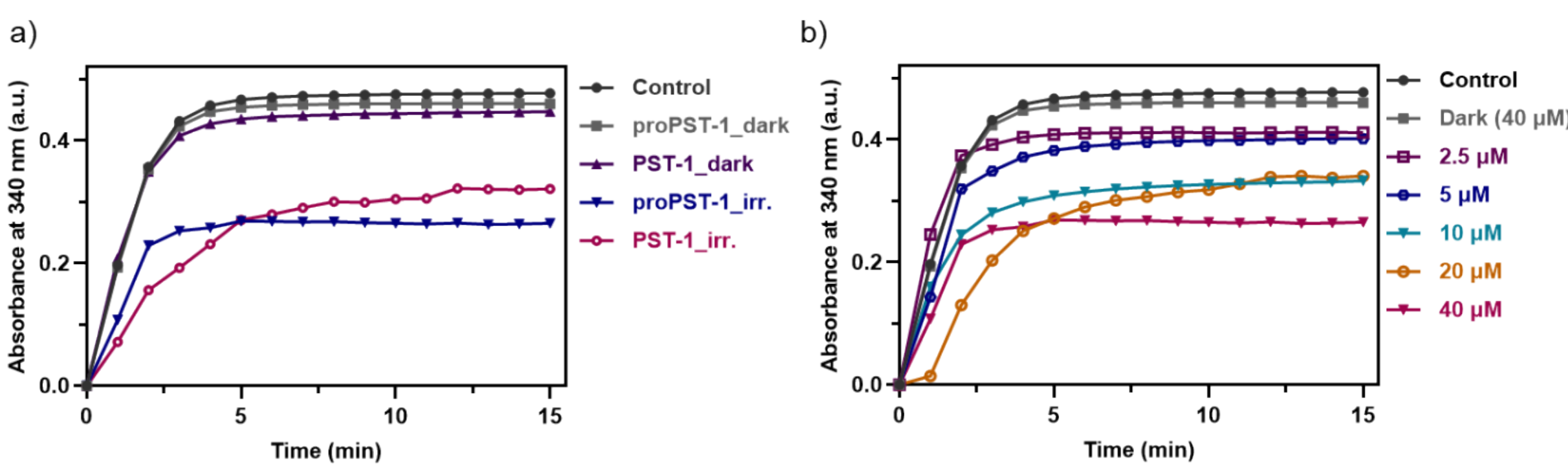

Figure S17. *In* vitro tubulin polymerisation assay monitored by UV-Vis spectroscopy at 340 nm. **a)** Comparison of tubulin polymerisation inhibition in the presence of PST-1 (40 μM) and proPST-1 (40 μM) under dark and irradiated (365 nm, 5.5 mW/cm^2) conditions. Samples were either shielded from light or irradiated with 365 nm light prior to measurement. Upon irradiation, proPST-1 exhibited inhibition of tubulin polymerisation comparable to irradiated PST-1, indicating activation consistent with photocleavage and photoswitching. In contrast, proPST-1 showed minimal effect on tubulin polymerisation in dark. **b)** Concentration dependent (2.5-40 μM) inhibition of tubulin polymerisation in the presence of proPST-1 upon irradiation. proPST-1 solution was irradiated for 30 min prior to addition to ensure maximum photocleavage.

concentration) was added with mixing and the change in absorbance at 340 nm was monitored for 15 min. The samples were either shielded from light or irradiated with 365 nm light (5.5 mW/cm^2) in between the measurements.

1.13 *In vitro* tubulin polymerisation assay by fluorescence microscopy (proPST-1)

Tubulin protein (HiLyte FluorTM 488 labelled, from porcine brain) (Cat. #TL488M), General tubulin buffer (Cat. #BST01), G-PEM buffer with 60% (w/v) glycerol (Cat. #BST05), Taxol (TXD01) and GTP (Cat. #BTS06) were obtained from Cytoskeleton, Inc. Tubulin polymerisation reactions were performed at 37 °C and visualised using a Nikon N-STORM fluorescence microscopy system via excitation of the sample in green channel (561 nm laser, 10% intensity). Stock solutions were prepared using the manufacturer's instruction manual.

For tubulin growth, 1 µL of tubulin stock solution, 20 µL of Taxol stock solution, and the required amount of

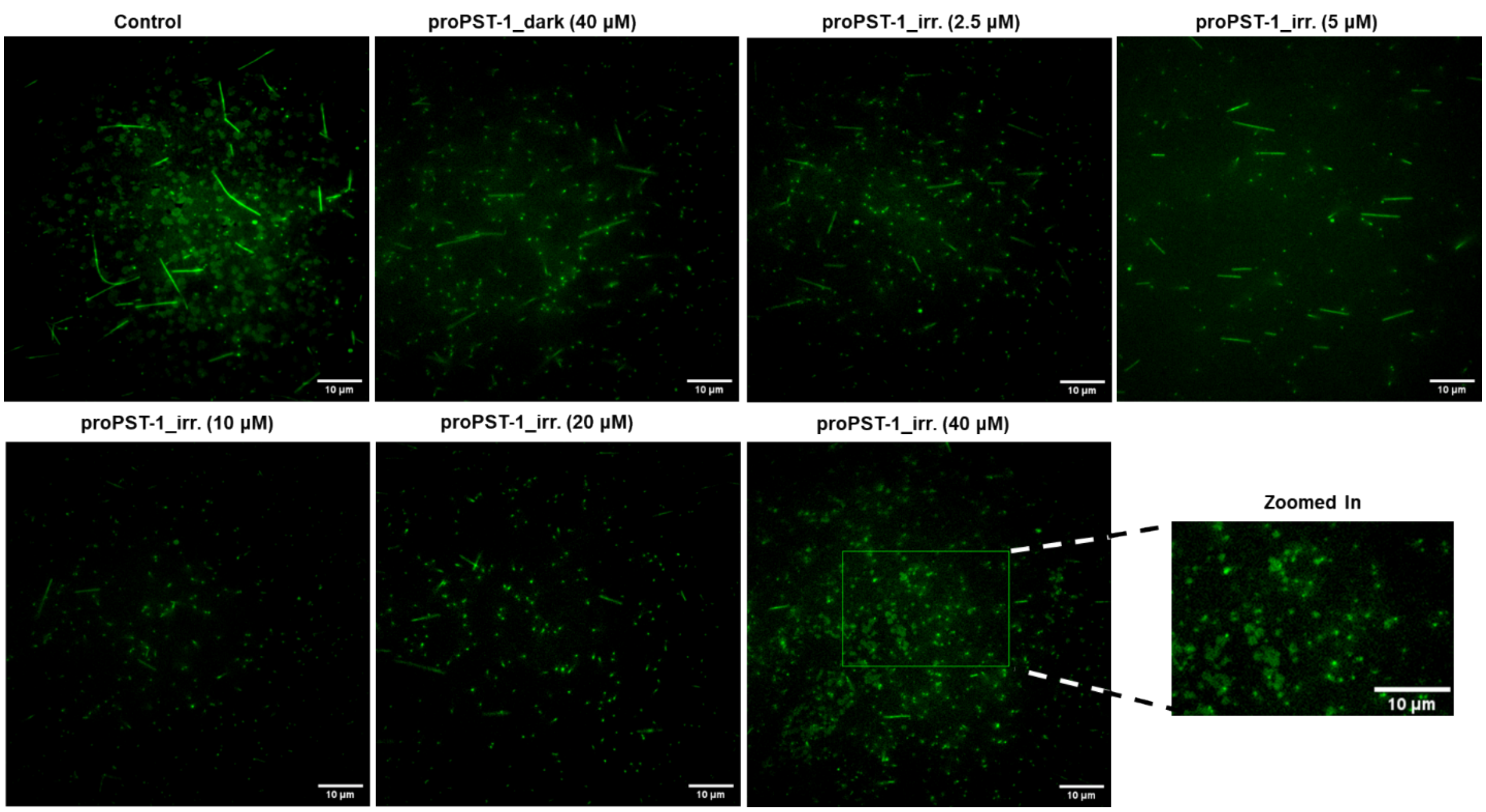

Figure S18. Fluorescence microscopy images of tubulin polymerisation reactions in the presence of proPST-1. Tubulin was incubated with control buffer, proPST-1 in the dark (40 µM), or proPST-1 pre-irradiated with 365 nm light (5.5 mW/cm^2, 30 min) at concentrations ranging from 2.5 to 40 µM. Irradiated samples exhibited a concentration dependent decrease in microtubule formation compared with the control and dark treated samples, indicating photo triggered generation of the active tubulin polymerisation inhibitor. The inset shows a magnified region of the 40 µM irradiated sample. Scale bars = 10 µm.

stock solutions of either G-PEM plus 30%glycerol buffer (control) or proPST-1 (in DMSO) (maintained in dark or pre-irradiated with 365 nm light for 30 minutes, 5.5 mW/cm^2). The remaining volume was adjusted with G-PEM plus 30% glycerol buffer to obtain a final volume of 200 µL. Finally, 50 µL of the solution was immediately pipetted onto a glass slide and imaged by microscope.

1.14 Encapsulation of proPST-1 in amphiphilic polymer

1.14.1 Procedure for encapsulation of proPST-1 in amphiphilic polymer to generate proPST-1@PN

proPST-1 was encapsulated in the amphiphilic polymer using a previously reported protocol.[5] Depending on the encapsulation ratio (proPST-1:PN), the required amounts of polymer and proPST-1 were co-dissolved in dichloromethane (DCM) and transferred to an aluminium foil wrapped glass vial. DCM was gently allowed to evaporate by nitrogen to form a thin film on glass, and this film was dried completely under vacuum at 30 °C overnight. To this vial, Milli-Q water was added, vortexed, and sonicated for 15 min. The solution equilibrated at room temperature in the dark overnight by suspending the vial in air overnight so that the vial was not in contact with the cold surface.

1.14.2 Procedure for determining the encapsulation efficiency (% E.E.) of proPST-1 in amphiphilic polymer (proPST-1@PN)

1 mL of proPST-1-encapsulated polymeric nanoparticles proPST-1@PN were prepared according to the protocol described above (section 1.14.1), with polymer:proPST-1 molar ratio = 1:10. Calibration curves of proPST-1 were created using a series of concentrations from 2.22 – 116.27 μM. Later, proPST-1 encapsulated nanoparticles (proPST-1@PN) were added to centrifuge tubes and centrifuged for 15 min at 8000 RPM to

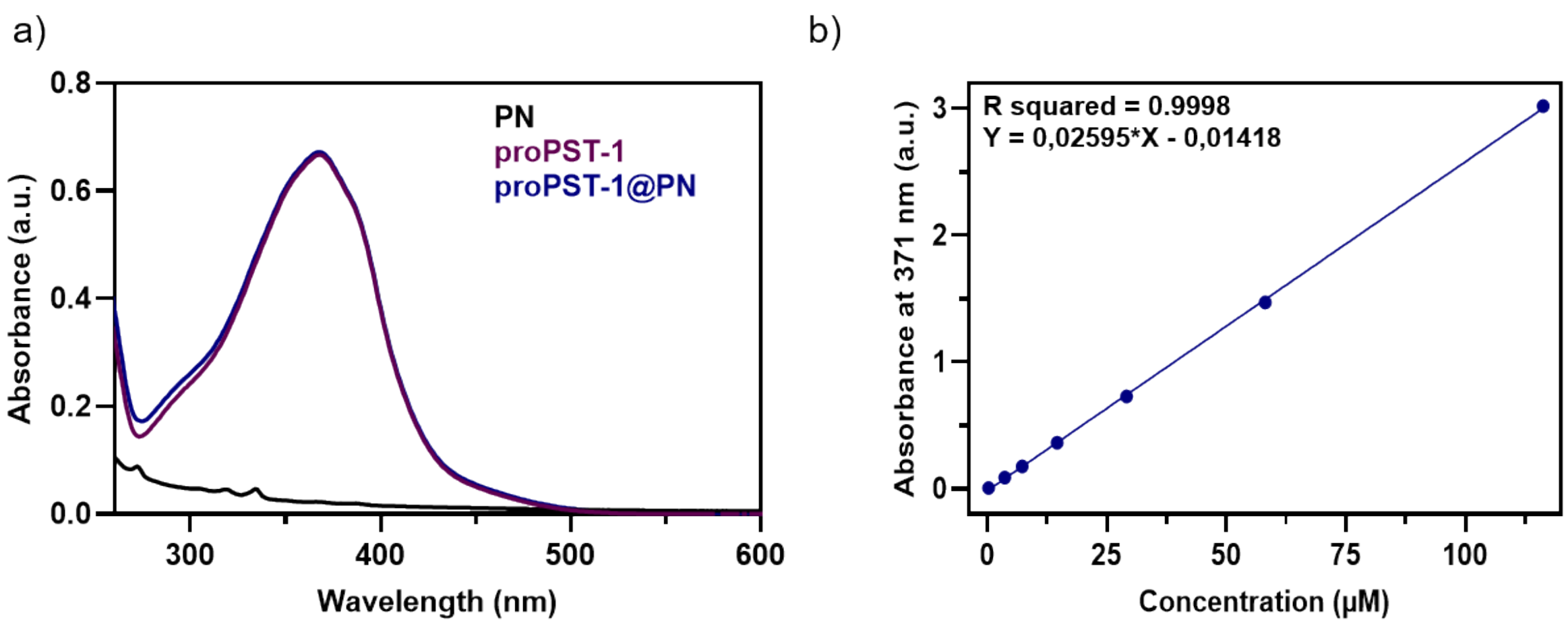

Figure S19. Determination of the encapsulation efficiency of proPST-1 in polymeric nanoparticles (proPST-1@PN) at room temperature. **a)** UV-Vis absorption spectra of PN (10 μM), free proPST-1 ((10 μM)), and proPST-1@PN (PN =2.5 μM and proPST-1 =25 μM) in acetonitrile/water (ACN/H_2O, 1/1, v/v), showing the characteristic absorption maximum of proPST-1 at 371 nm, which was used for quantitative analysis. (b) Calibration curve of proPST-1 at 371 nm constructed from solutions of known concentrations (2.22–116.27 μM) in acetonitrile/water (ACN/H_2O, 1/1, v/v).

remove any non-encapsulated proPST-1 as precipitate.[6] The supernatant containing proPST-1@PN was analysed by UV-Vis spectroscopy to determine the concentration of encapsulated proPST-1. Encapsulation efficiency was determined using equation below:

$$\%E.E.= \left(\frac{\text{moles of proPST-1 in supernatant}}{\text{initial moles of proPST-1}}\right) \times 100$$

Table S3. Encapsulation efficiency of proPST-1 in polymeric nanoparticles prepared at different polymer feed ratios at room temperature. Formulations prepared at low polymer contents (3 and 4) produced turbid dispersions, preventing reliable determination of encapsulation efficiency.

	PN:proPST-1 (mol/mol)	PN:proPST-1 (wt./wt.)	Encapsulation Efficiency (%)
1.	1:5	73.6:1	99.9 ± 2.1
2.	1:10	36.8:1	94.9 ± 5.1
3.	1:20	18.4:1	Not determined (turbid dispersion)
4.	1:50	7.4:1	Not determined (turbid dispersion)

1.15. Dynamic light scattering (DLS) measurements

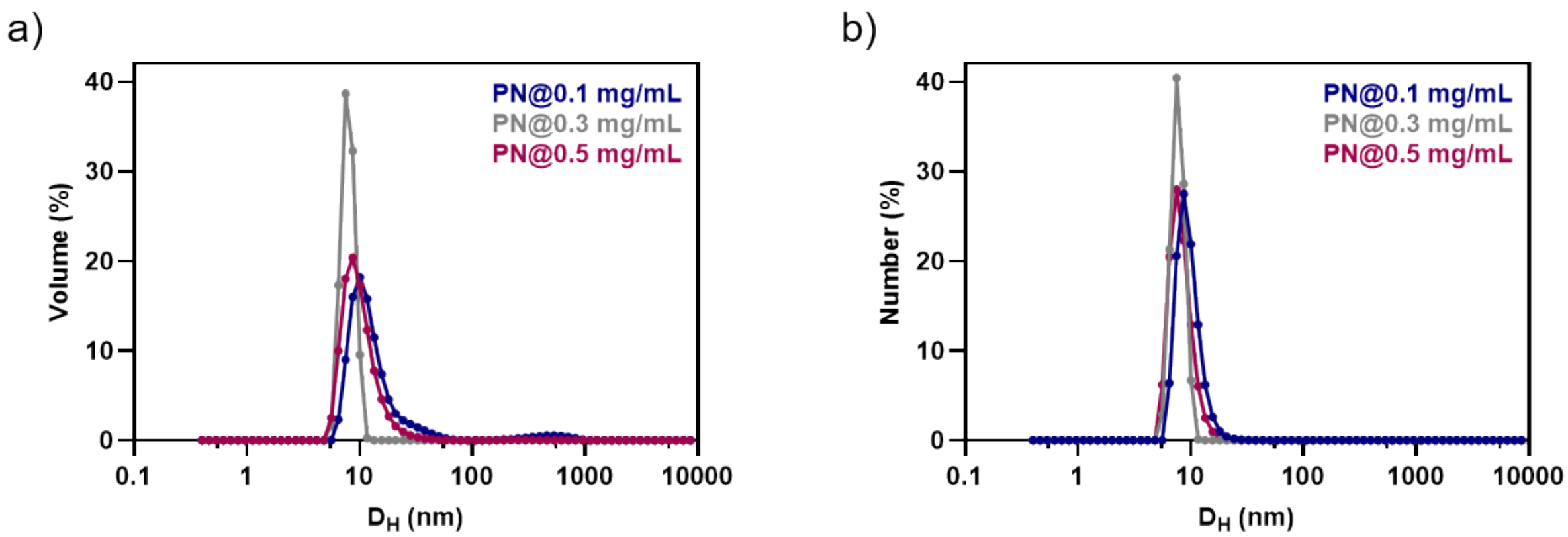

Figure S20. Dynamic light scattering (DLS) analysis (filtered) **a)** Volume plot, and **b)** Number plot of unloaded polymeric nanoparticles (PN) at concentrations of 0.1, 0.3, and 0.5 mg/mL in Milli-Q water at room temperature.

For the sample preparation of DLS measurements, the required amounts of polymer and proPST-1 were co-dissolved in dichloromethane (DCM) and transferred to an aluminium foil wrapped glass vial. DCM was gently allowed to evaporate by nitrogen flow to form a thin film on glass, and this film was dried completely under vacuum at 30 °C overnight. To this vial, Milli-Q water was added, vortexed, and sonicated for 15 min. The solution was then suspended in air overnight to equilibrate to room temperature in dark. Samples were measured before and/or after filtration over an 0.2 μm PVDF filter prior to measurements to remove any dust particles perturbing the measurement. For irradiation, samples were exposed to 365 nm light (5.5 mW/cm^2) for 30 min and then one minute every hour for 24 h prior to the measurements.

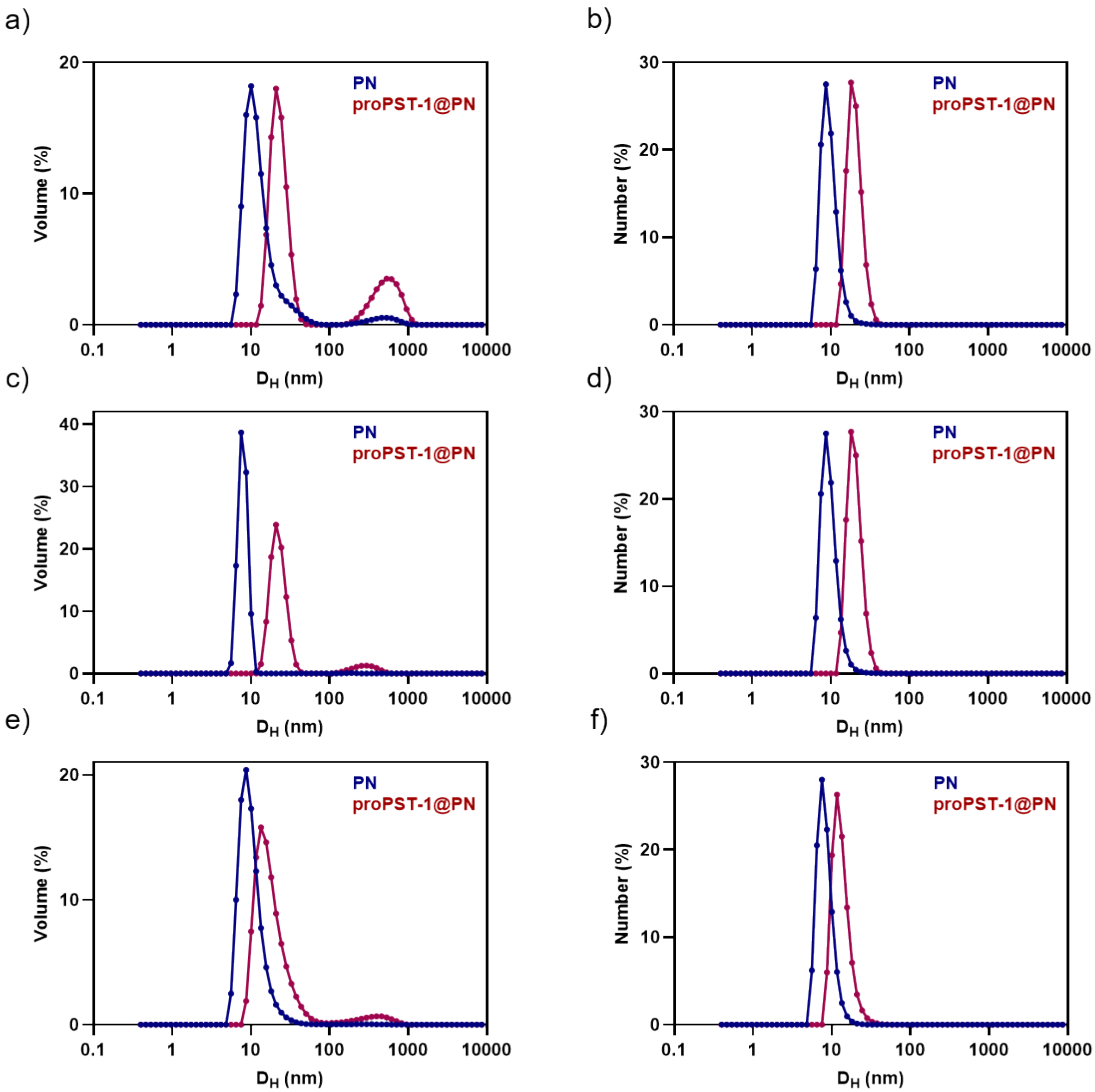

Figure S21. Dynamic light scattering (DLS) size distributions (filtered) of unencapsulated polymeric nanoparticles (PN) and proPST-1 loaded polymeric nanoparticles (proPST-1@PN) in Milli-Q water at room temperature. Volume plot (a, c and e) and number plot (b, d and f) size distributions measured at concentrations of 0.1 mg/mL (a and b), 0.3 mg/mL (c and d), and 0.5 mg/mL (e and f). In all the cases, the molar ratio of PN to proPST-1 was kept at 1:10. proPST-1@PN exhibited larger hydrodynamic diameters than unloaded PN, indicating incorporation of the proPST-1 within the nanoparticle formulation.

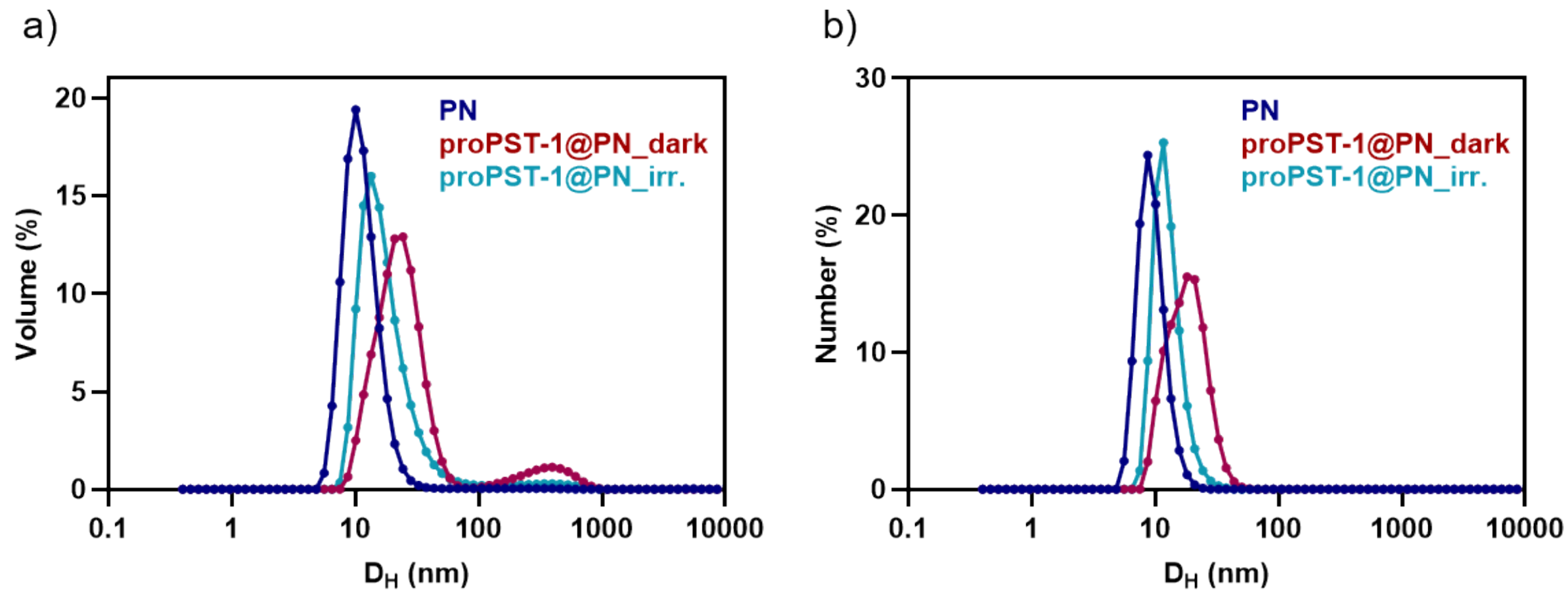

Figure S22. Dynamic light scattering (DLS) size distributions (filtered) of unencapsulated polymeric nanoparticles (PN), proPST-1 encapsulated polymeric nanoparticles in the dark (proPST-1@PN_dark), and proPST-1 encapsulated polymeric nanoparticles after irradiation (proPST-1@PN_irr.) in Milli-Q water at room temperature (0.5 mg/mL). **a)** Volume plot, D_H for PN = 10.1 nm, proPST-1@PN_dark = 24.4 nm, and proPST-1@PN_irr = 13.5 nm, and **b)** Number plot, D_H for PN = 8.7 nm, proPST-1@PN_dark = 21.0 nm, and proPST-1@PN_irr = 11.7 nm Irradiation (365 nm, 5.5 mW/cm²) resulted in a shift of the size distribution towards smaller hydrodynamic diameters relative to the non-irradiated formulation. In all the cases, the molar ratio of PN to proPST-1 was kept at 1:10.

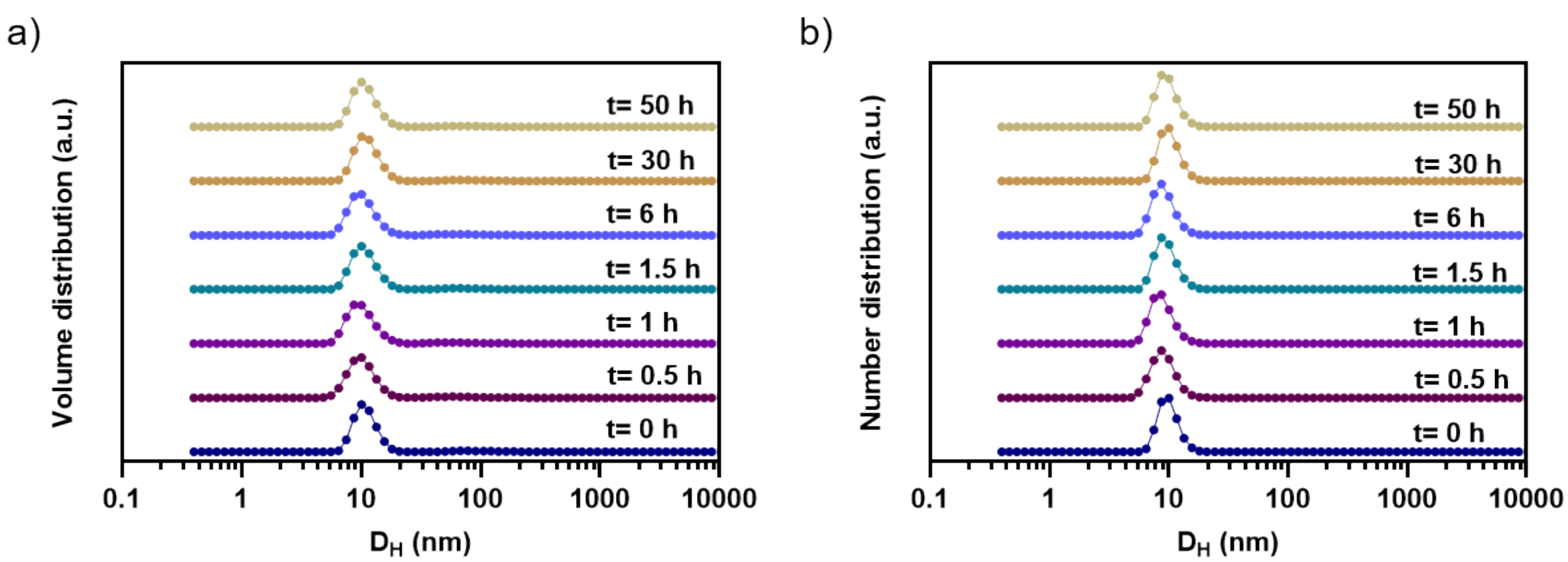

Figure S23. Effect of UV irradiation (365 nm, 5.5 mW/cm²) on the size distribution of empty polymeric nanoparticles (PNs). Dynamic light scattering (DLS) measurements of filtered PNs (0.5 mg/mL in Milli-Q water) under UV irradiation (1.5 h of continuous irradiation followed by 5 min/h for 24 times over a period of 50 h) shown as (a) volume plot, and (b) number plot. Measurements were performed after 0, 0.5, 1, 1.5, 6, 30, and 50 h of irradiation. The hydrodynamic size distributions remained largely unchanged over the 50 h period, indicating good stability of the PNs under UV exposure.

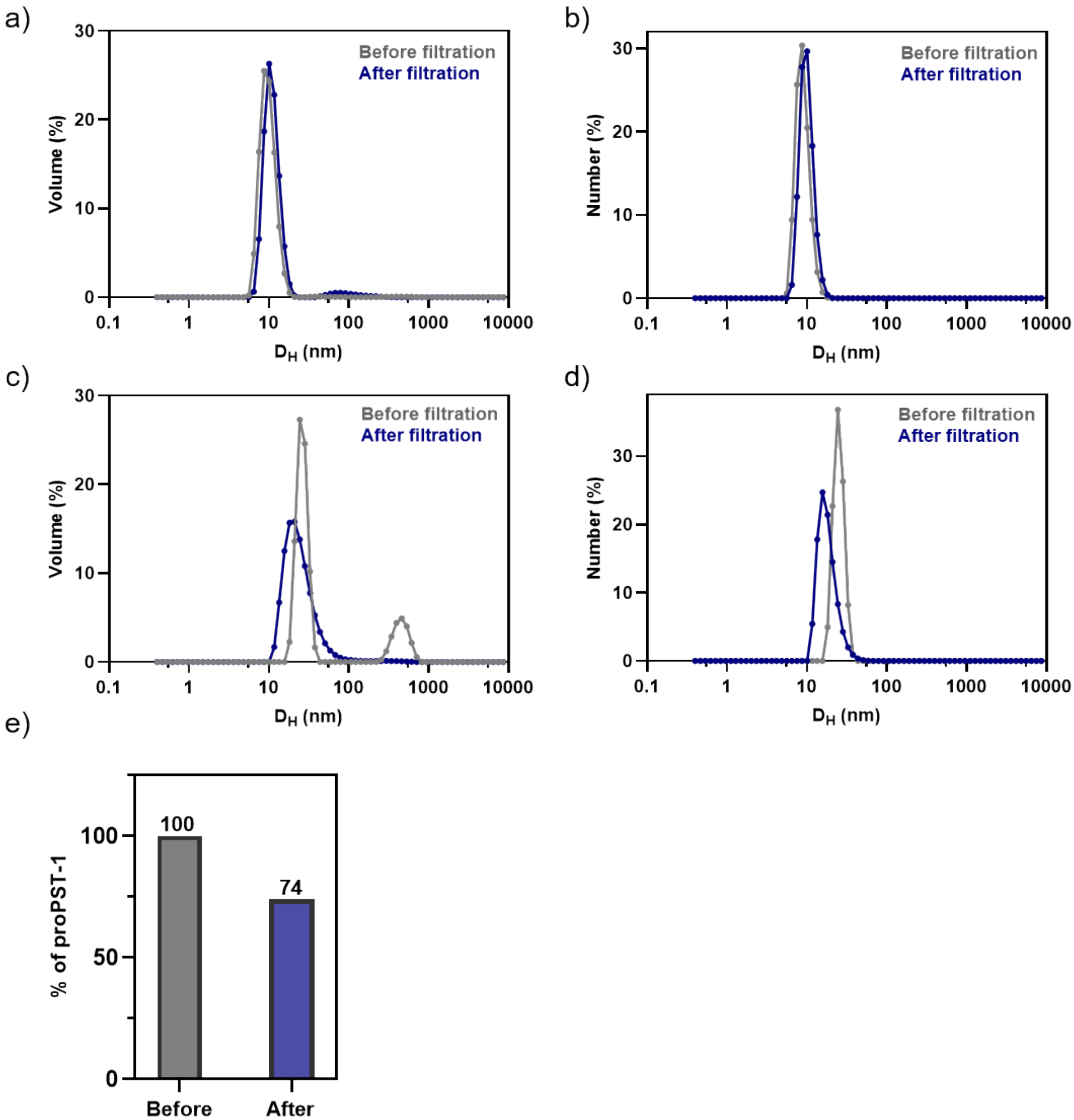

Figure S24. Dynamic light scattering (DLS) size distributions before and after filtration of (a, b) unencapsulated polymeric nanoparticles (PN, 0.5 mg/mL) and (c, d) proPST-1 loaded polymeric nanoparticles (proPST-1@PN, 0.5 mg/mL) in Milli-Q water at room temperature. (e) Percentage of proPST-1 retained after filtration relative to the amount present before filtration, as determined by absorbance measurements. Approximately 74% of proPST-1 was retained after filtration. In all cases, the molar ratio of PN to proPST-1 was kept at 1:10. While filtration caused no appreciable change in the size distribution of PN, proPST-1@PN exhibited decrease in hydrodynamic diameters post filtration consistent with the partial loss of proPST-1 during filtration.

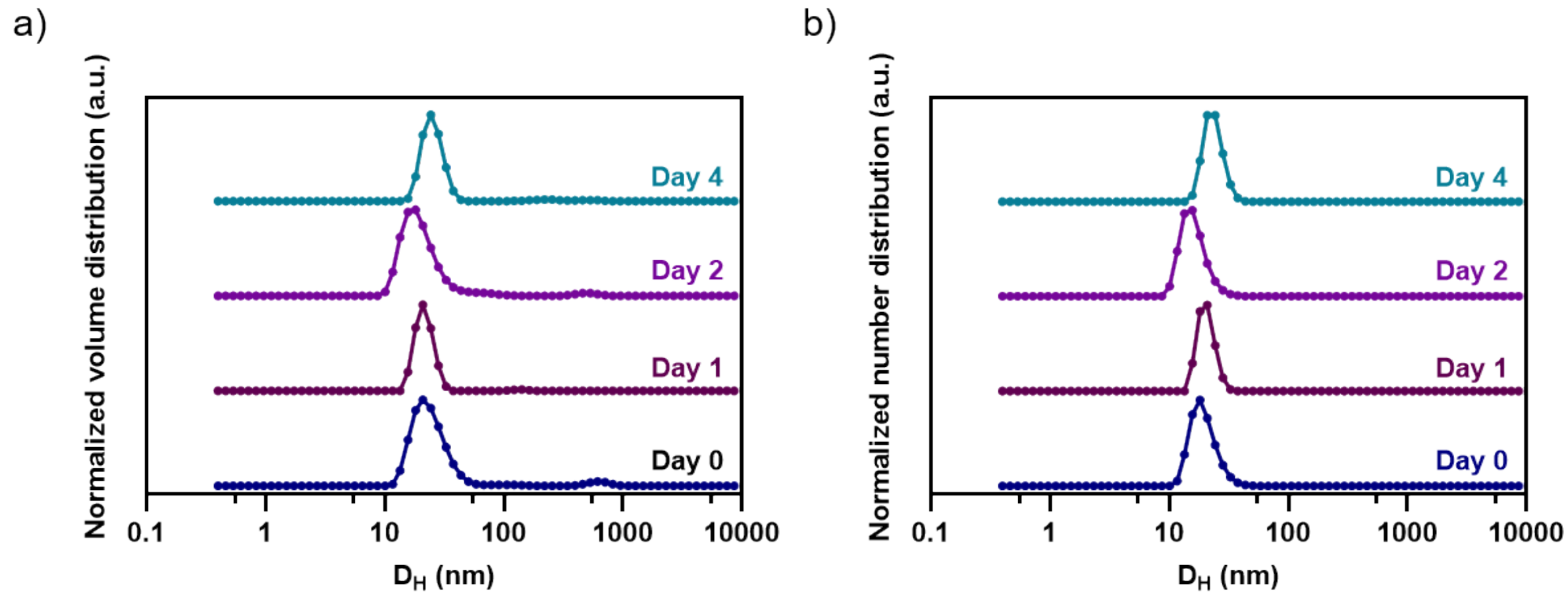

Figure S25. Dynamic light scattering (DLS) size distributions of unfiltered proPST-1@PN over a period of 4 days (0.5 mg/mL in PBS, pH = 7.4) shown as (a) Normalized volume plot, and (b) Normalized number plot. Molar ratio of PN to proPST-1 was kept at 1:10, and samples were kept in dark at room temperature between the measurements. The size distribution remained largely unchanged, suggesting good stability of proPST-1@PN in PBS in dark.

1.16 *In vitro* tubulin polymerisation assay by UV-Vis spectroscopy (proPST-1@PN)

The experiments were performed according to the protocol described in section 1.12. proPST-1@PN was either kept in dark (proPST-1@PN_dark) or irradiated (proPST-1@PN_irr.) overnight at room temperature prior to

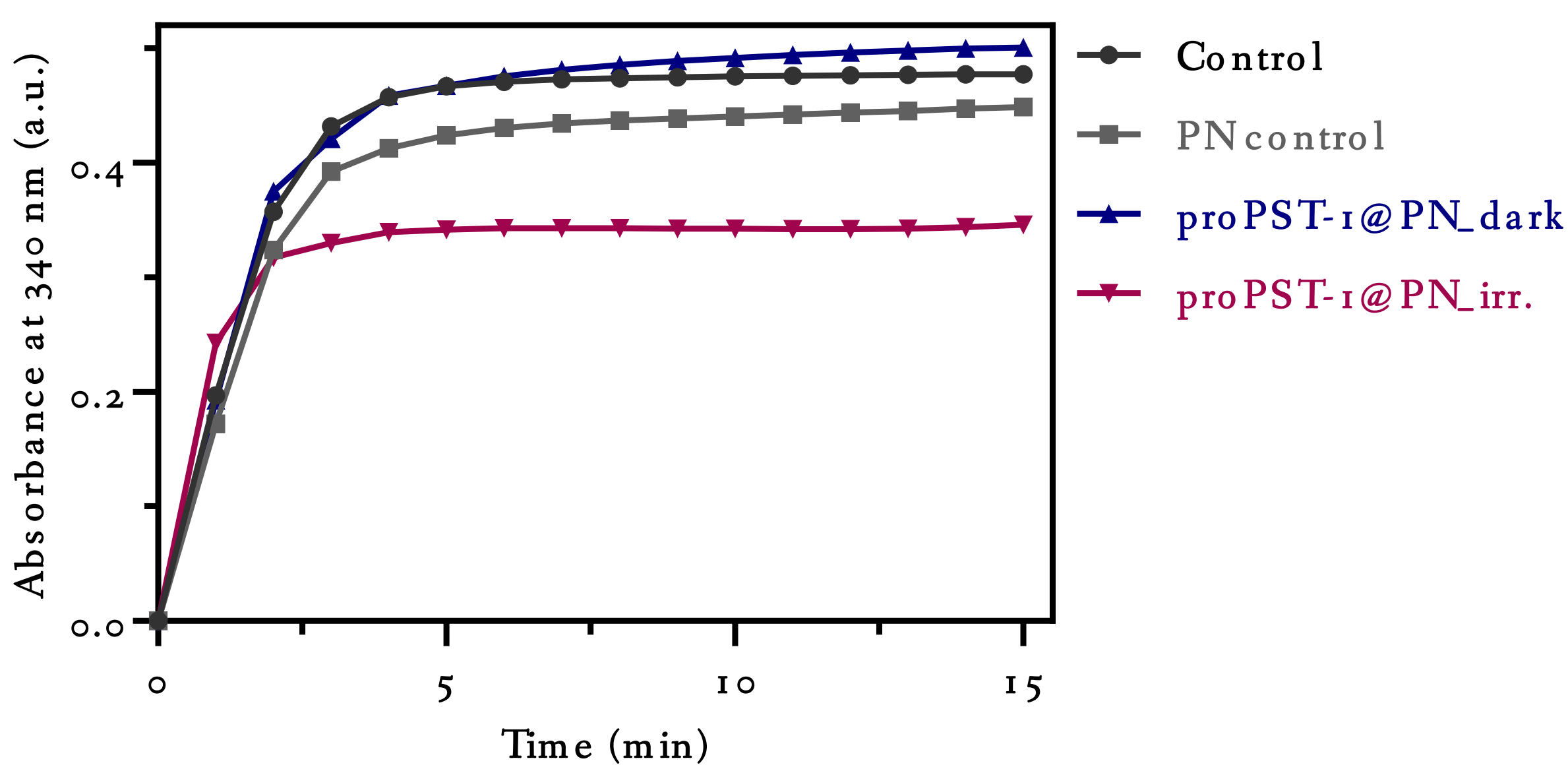

Figure S26. *In* vitro tubulin polymerisation assay monitored by UV-Vis spectroscopy at 340 nm in the presence of buffer control, polymeric nanoparticles (PN control, 4 μM), proPST-1 encapsulated polymeric nanoparticles in the dark (proPST-1@PN_dark, [PN]:[proPST-1] = 1:10 (4 μM:40 μM)), and proPST-1 encapsulated polymeric nanoparticles after irradiation (proPST-1@PN_irr., [PN]:[proPST-1] = 1:10 (4 μM:40 μM)). Irradiated proPST-1@PN exhibited reduced tubulin polymerisation relative to the non-irradiated formulation, whereas PN control showed minimal effect compared to the control.

addition to the reaction mixture.

1.17 *In vitro* tubulin polymerisation assay by fluorescence microscopy (proPST-1@PN)

The experiments were performed according to the protocol described in section 1.13 proPST-1@PN was either kept in dark (proPST-1@PN_dark) or irradiated (proPST-1@PN_irr.) overnight at room temperature prior to addition to the reaction mixture.

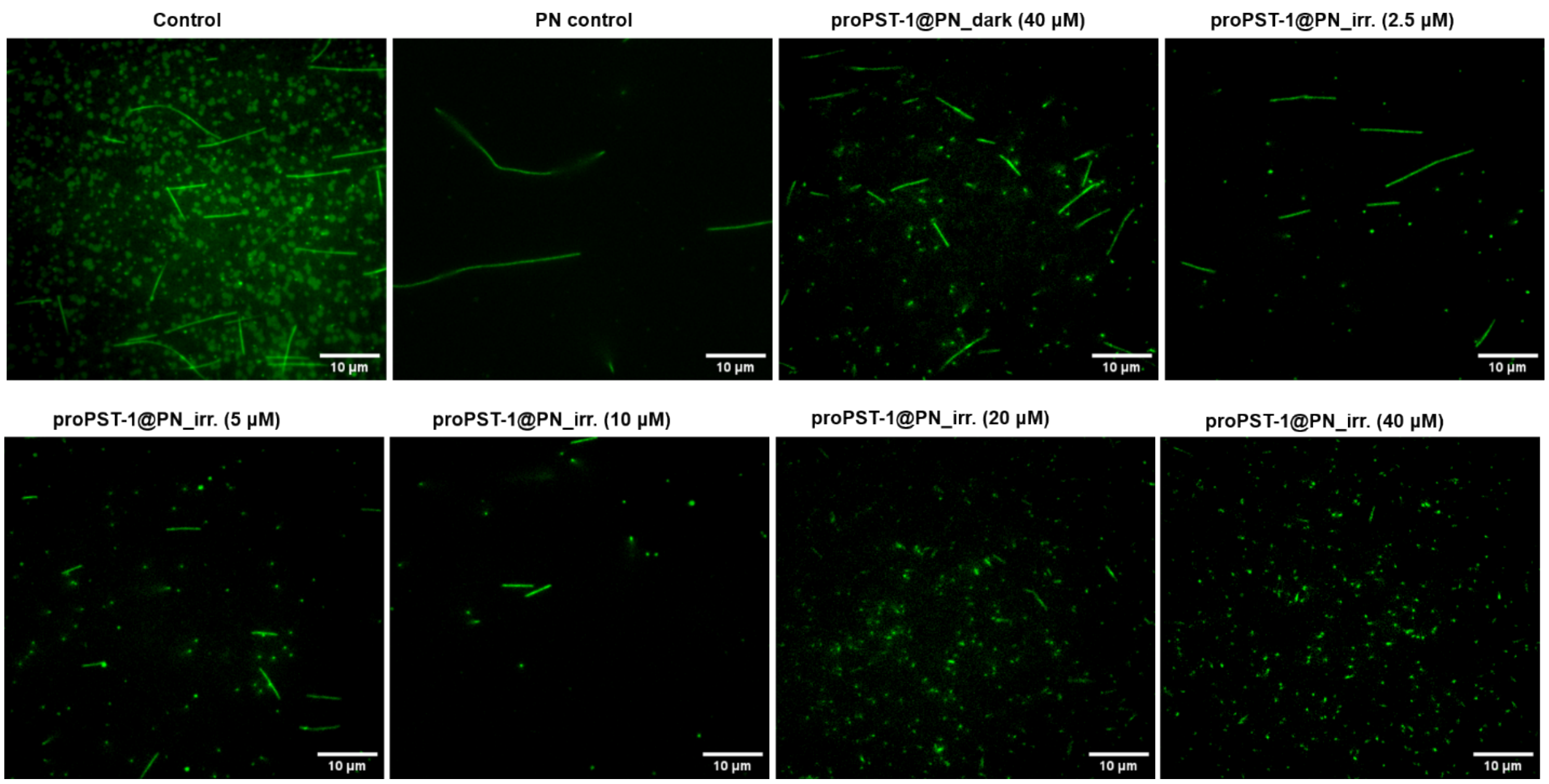

Figure S27. Fluorescence microscopy images of tubulin polymerisation reactions in the presence of proPST-1@PN. Tubulin was incubated with control buffer, PN control (4 μM), proPST-1 encapsulated polymeric nanoparticles in the dark (proPST-1@PN_dark ([PN]:[proPST-1] = 1:10 (4 μM:40 μM)), and proPST-1 encapsulated polymeric nanoparticles after irradiation (proPST-1@PN_irr.) at proPST-1 concentrations of 2.5, 5, 10, 20, 40 μM ([PN]:[proPST-1] = 1:10). Irradiated samples exhibited a decrease in microtubule formation compared with the control and dark treated samples, indicating photo triggered generation of the active tubulin polymerisation inhibitor. Scale bars = 10 μm

1.18 Cell viability assay

HeLa/SH-SY5Y cells (obtained from ATCC) were cultured and seeded in Dulbecco's modified Eagle medium (DMEM) supplemented with 10% (v/v) fetal bovine serum (FBS) and 1% (v/v) penicillin-streptomycin. Cytotoxicity of substrates were studied using the Cell Counting Kit-8 (CCK-8) assay. 5,000 cells containing suspension in cell culture media (100 μL) were seeded in a 96-well plate and incubated at 37 °C in a humidified atmosphere containing 5% CO_2 flow for 24 h. After 24 h, the cells were treated with different concentrations of proPST-1/proPST-1@PN/PN only (from freshly prepared stock solutions in DMSO or Milli-Q water such that the final concentration of DMSO/Milli-Q remained 1%). The plates were either shielded from light or irradiated with 365 nm light (1 min/h, 5.5 mW/cm^2) for 48 h. Following 48 h of treatment, the media was removed and the cells were washed with PBS followed by addition of fresh media (90 μL) and CCK-8 (10 μL) and incubated

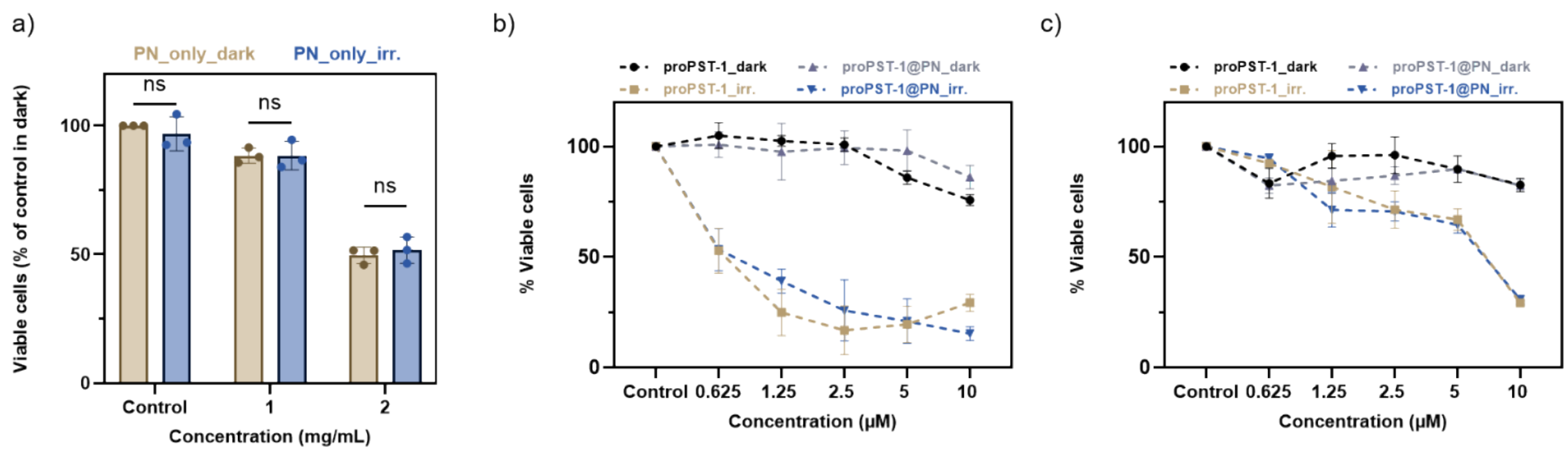

Figure S28. Cell viability of HeLa and SH-SY5Y cells following treatment with polymeric nanoparticles (PNs), free proPST-1, or proPST-1-loaded polymeric nanoparticles (proPST-1@PN) under dark and irradiated conditions. a) Viability of HeLa cells treated with empty PNs at 1 and 2 mg/mL under dark conditions or following 365 nm irradiation (5.5 mW/cm^2). Empty PNs were well tolerated at 1 mg/mL, and irradiation alone did not significantly affect cell viability. Statistical significance was assessed by two-way ANOVA followed by Šídák's multiple comparison test; ns is not significant. Cell viability of b) HeLa cells, and c) SH-SY5Y cells following treatment with free proPST-1 and proPST-1 encapsulated polymeric nanoparticles (proPST-1@PN) under dark and irradiated conditions. Cells were treated with free proPST-1 (from DMSO stock) or proPST-1 encapsulated polymeric nanoparticles (proPST-1@PN) under dark conditions or irradiated with 365 nm light (5.5 mW/cm^2) with final concentration of proPST-1: 0.625, 1.25, 2.5, 5, and 10 μM (for proPST@PN: [PN]:[proPST-1] = 1:10) for 48 h and cell viability was determined using the CCK-8 assay. Cell viability is expressed relative to untreated control cells. Irradiated samples exhibited concentration-dependent cytotoxicity, whereas dark-treated samples showed substantially reduced effects on cell viability. Data are presented as mean ± SD.

further for 3 h at 37 °C. After 3 h, the plate was taken out and absorbance was recorded at 450 nm in a microplate reader. Cell viability was measured relative to untreated control cells. For each concentration, three replicates were performed.

2. Characterisation Data

2.1. NMR Spectra

^{1}H-NMR of ONB-Br ($CDCl_3$, 400 MHz)

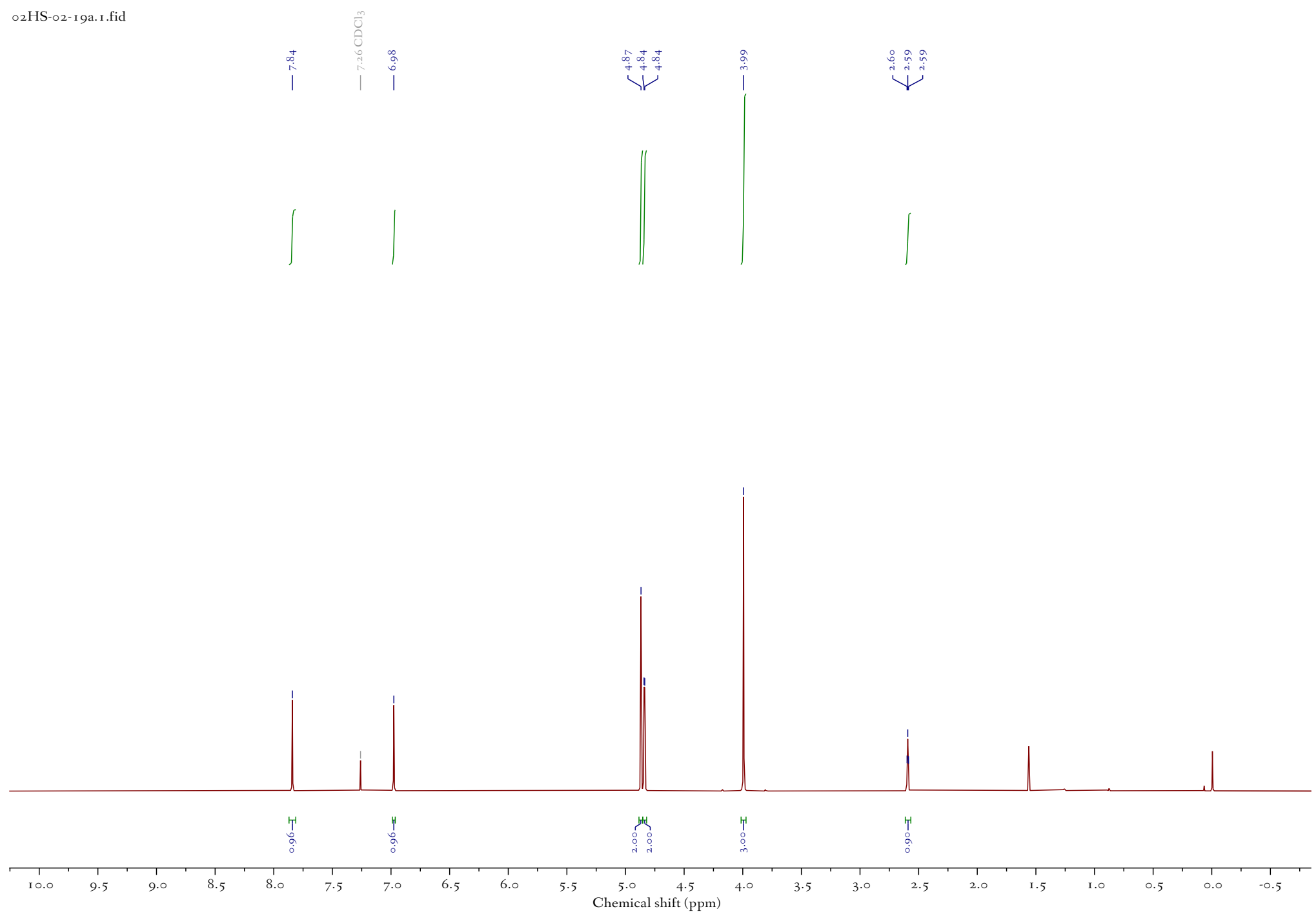

^{13}C-NMR of ONB-Br ($CDCl_3$, 100 MHz)

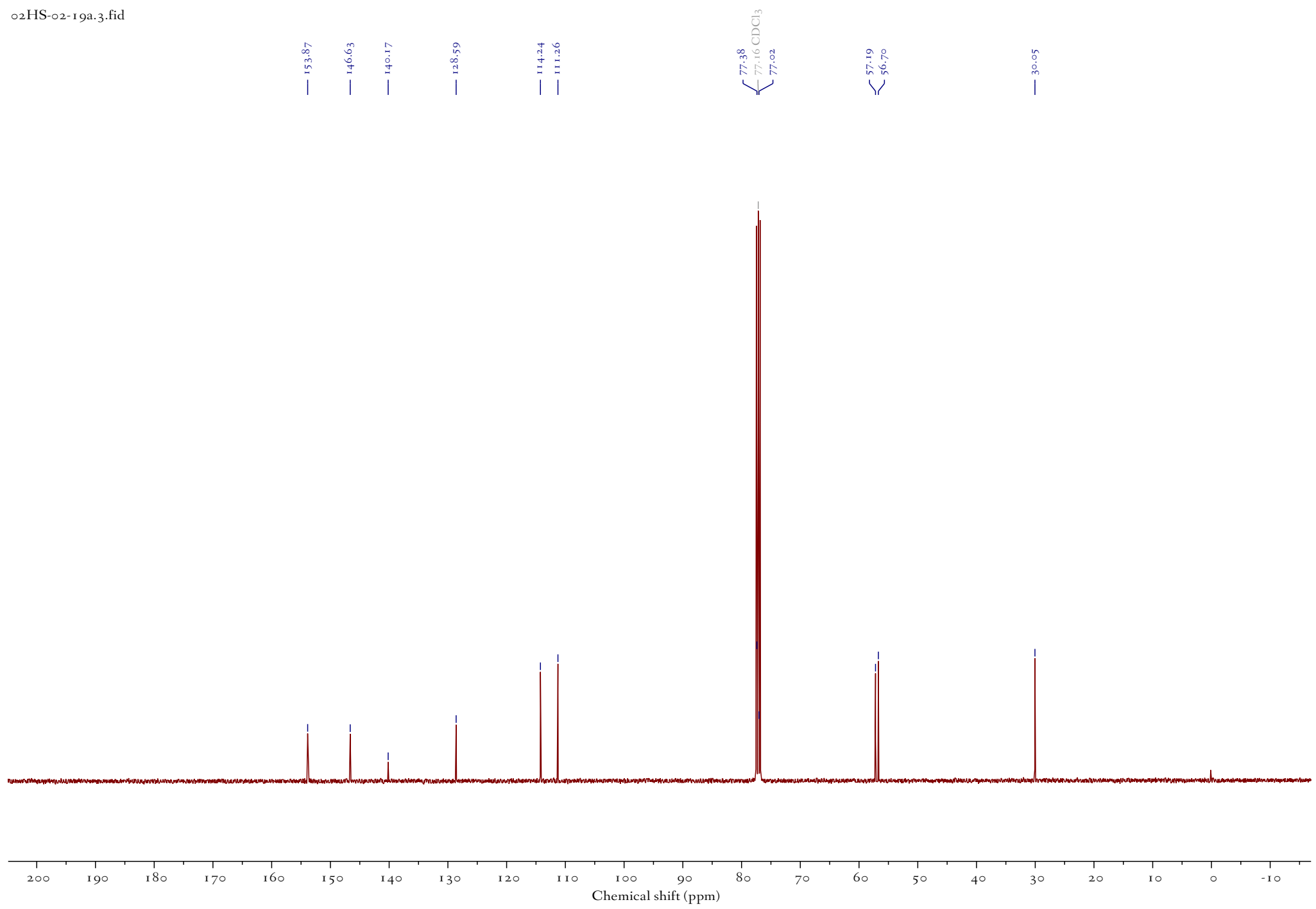

^{1}H-NMR of PST-1 ($CDCl_3$, 400 MHz)

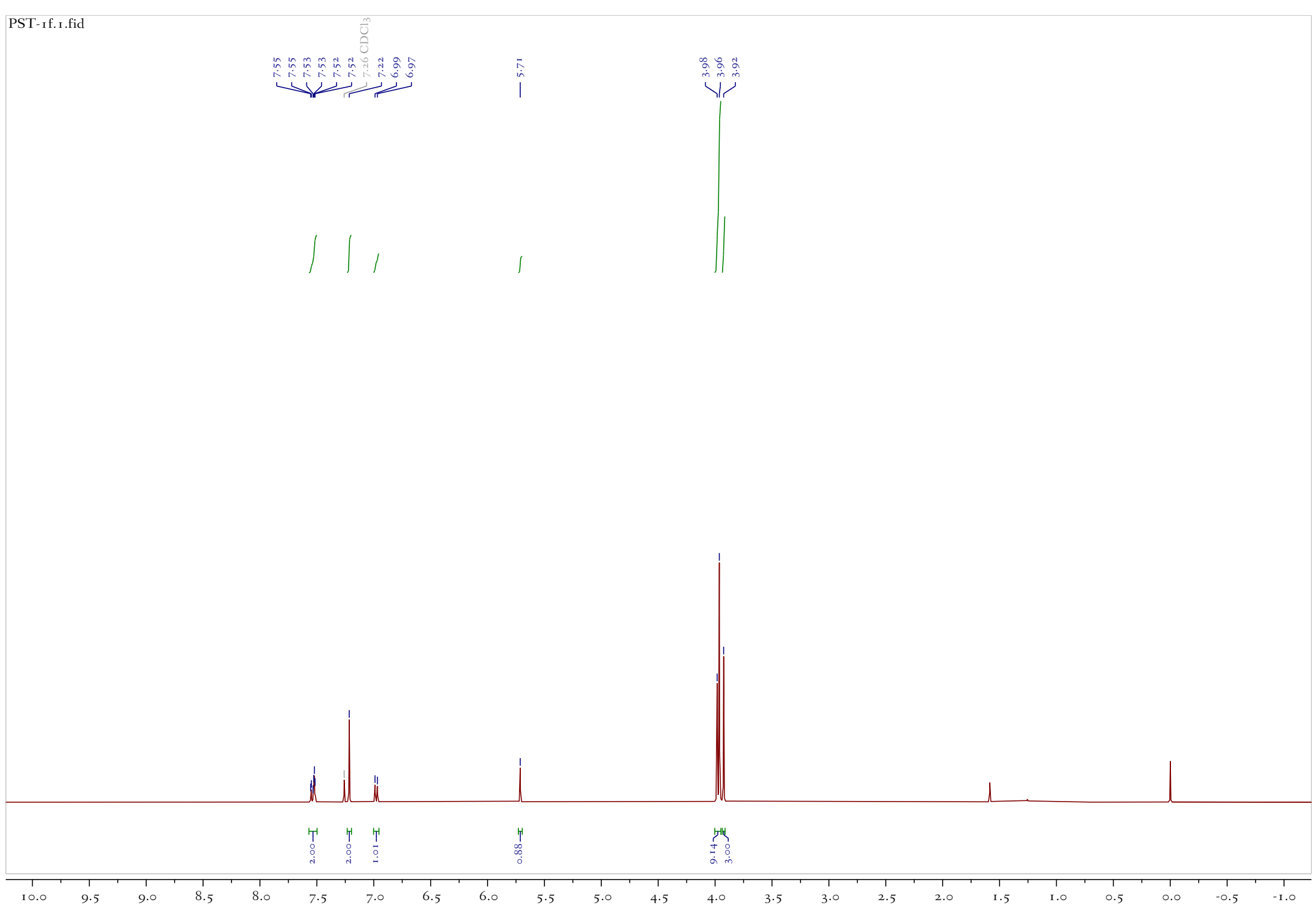

^{13}C-NMR of PST-1 ($CDCl_3$, 100 MHz)

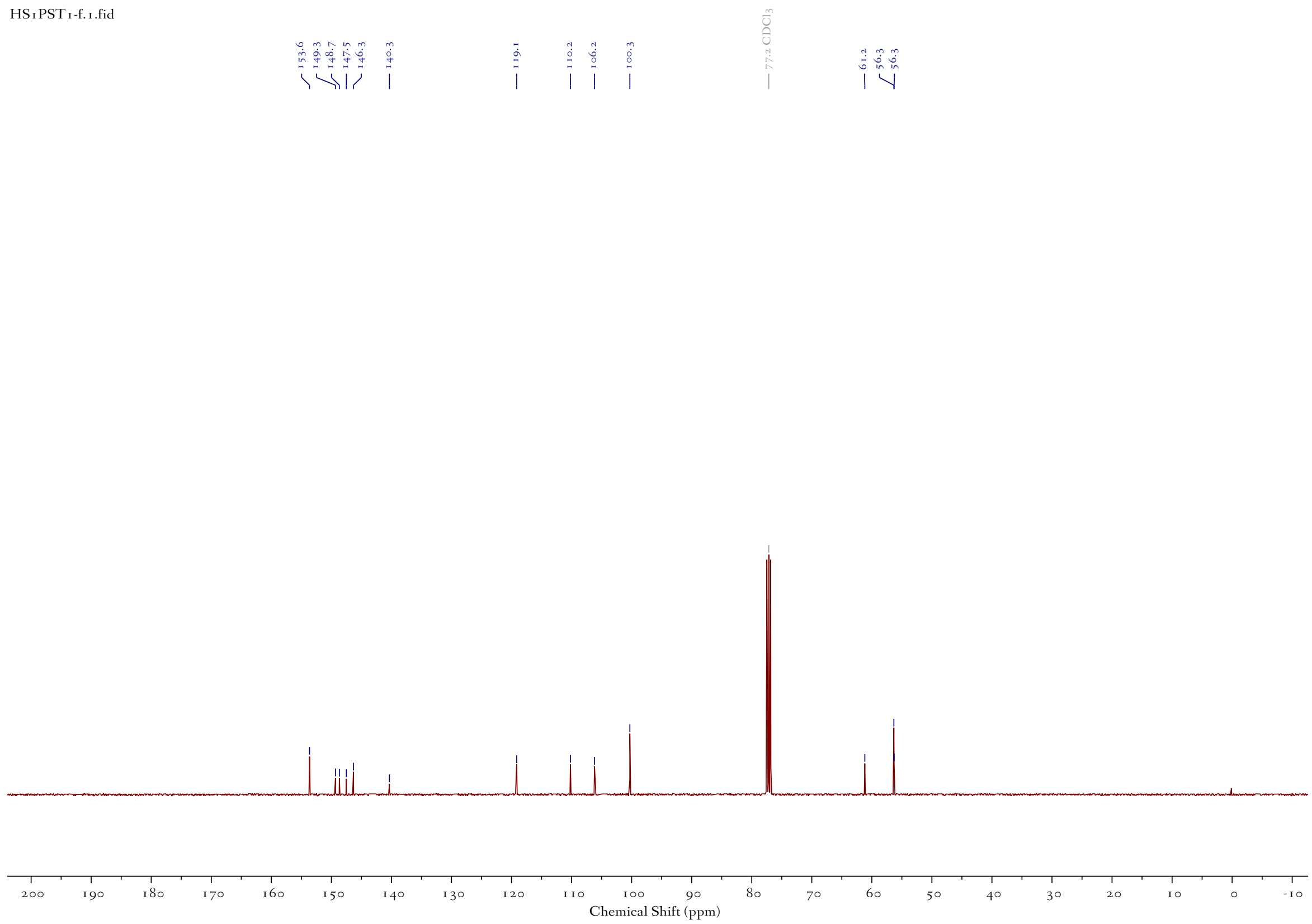

^{1}H-NMR of proPST-1 ($CDCl_3$, 400 MHz)

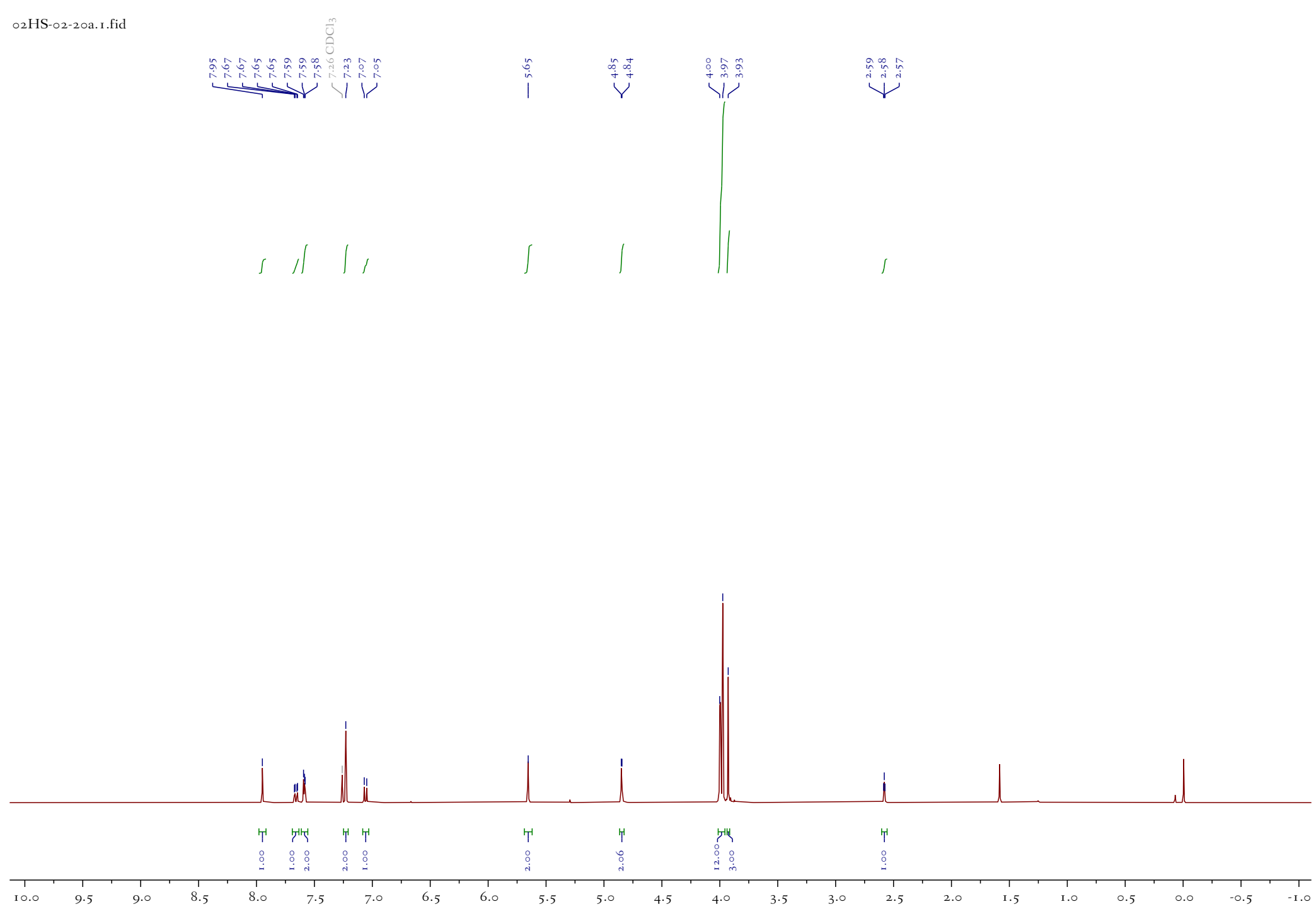

^{13}C-NMR of proPST-1 ($CDCl_3$, 100 MHz)

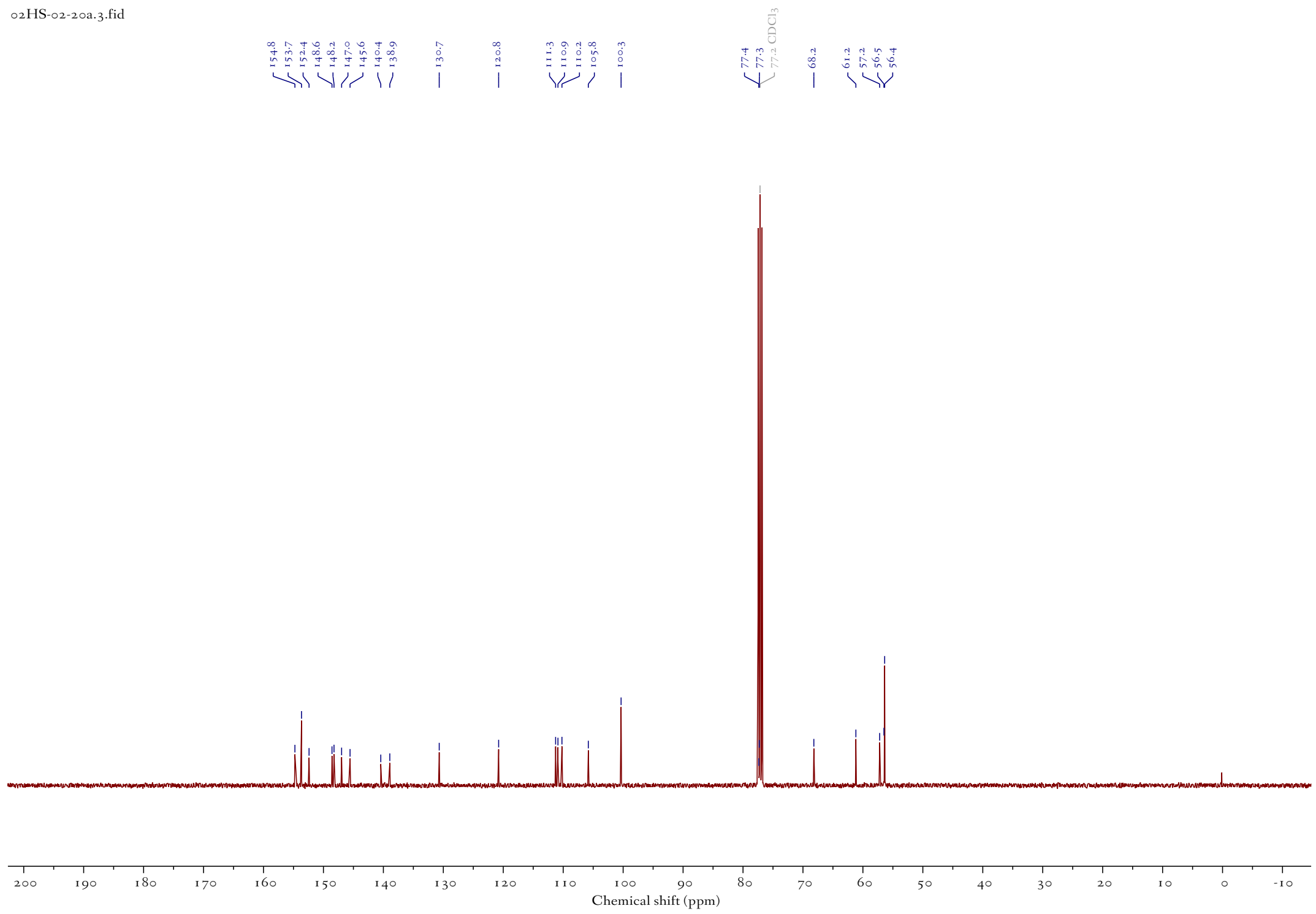

MALDI-ToF-MS Spectra of proPST-1

D:\Data\Users 2024\July\July1924\Harshit\02HS-02-20_DCTB\0_E4\1\1Ref

Comment 1 CHCA/DCTB in THF

Comment 2 Calibration in d:\Huachuan

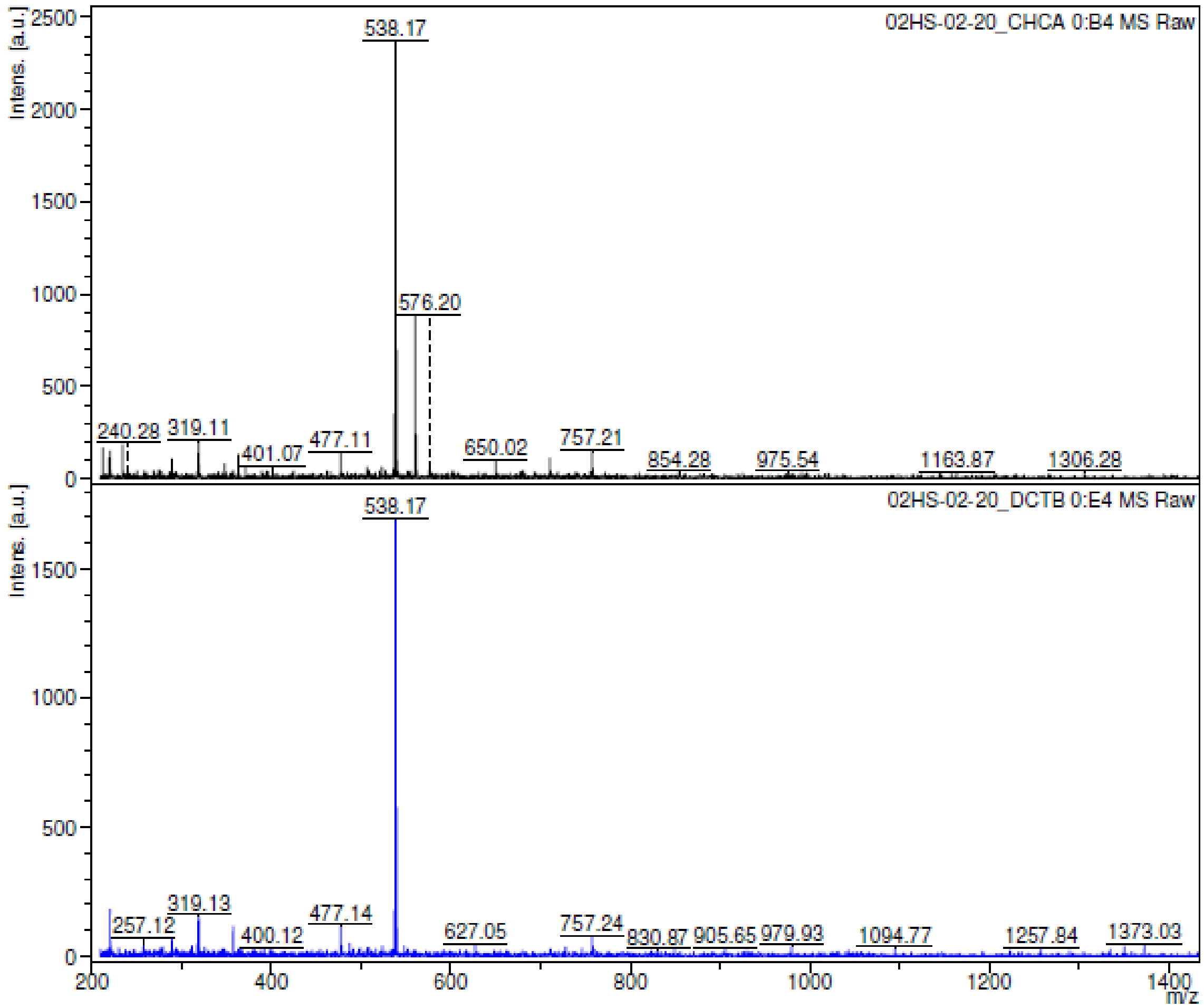

Acquisition Parameter

Date of acquisition	2024-07-19T14:01:40.148+02:00
Acquisition method name	D:\MALDI_Method\RP_200-3500_Da.par
Aquisition operation mode	Reflector
Voltage polarity	POS
Number of shots	500
Name of spectrum used for calibration	
Calibration reference list used	Cesium tri jodide positive mode

Instrument Info

User	smo379
Instrument	ST-A2130
Instrument type	autoflex

Bruker Daltonics flexAnalysis printed: 19-Jul-24 15:08:41

LC-MS trace of proPST-1

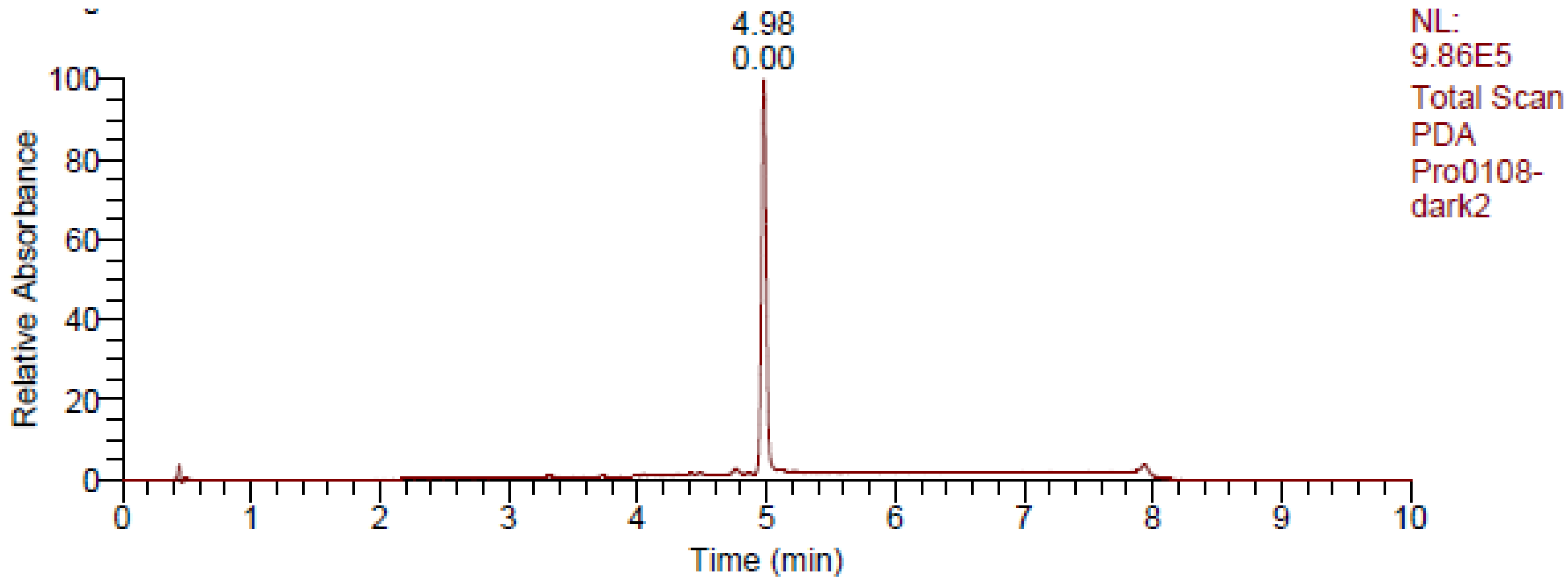

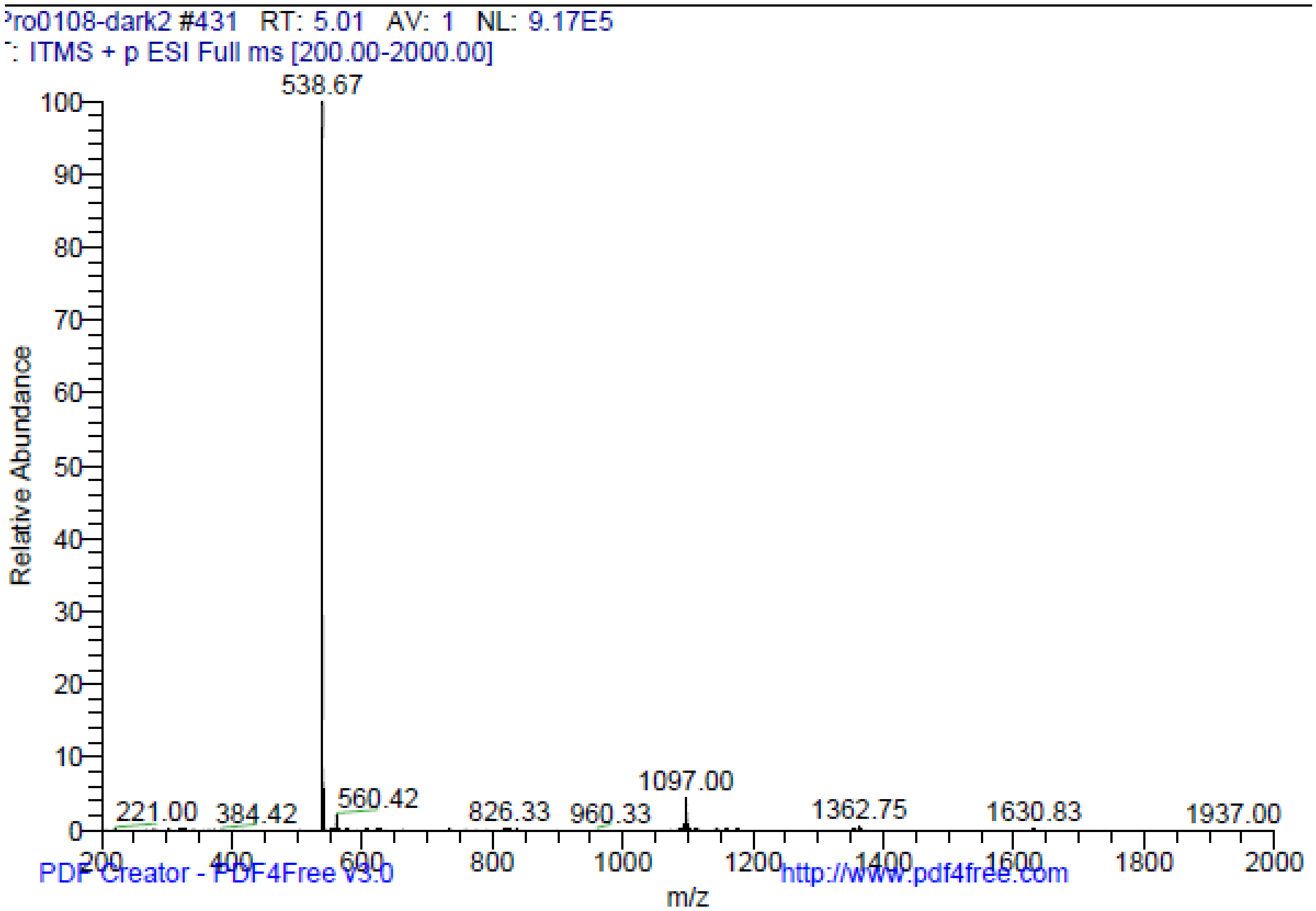

PDF Creator - PDF4Free v3.0 http://www.pdf4free.com

3. References

1 N. Hura, A. V. Sawant, A. Kumari, S. K. Guchhait and D. Panda, *ACS Omega*, 2018, **3**, 9754–9769.

2 W. P. da Silva, R. R. A. Caiana, M. E. S. B. Barros, J. C. R. Freitas, P. B. N. da Silva, G. G. C. Militão, R. A. Oliveira and P. H. Menezes, *Results Chem.*, 2024, **7**, 101539.

3 M. Borowiak, W. Nahaboo, M. Reynders, K. Nekolla, P. Jalinot, J. Hasserodt, M. Rehberg, M. Delattre, S. Zahler, A. Vollmar, D. Trauner and O. Thorn-Seshold, *Cell*, 2015, **162**, 403–411.

4 C. Chen, J. Zhao, M. Gao, X. Meng, A. Fan, Z. Wang and Y. Zhao, *Biomater. Sci.*, 2018, **6**, 511–518.

5 A. Sathyan, T. Loman, L. Deng and A. R. A. Palmans, *Nanoscale*, 2023, **15**, 12710–12717.

6 E. Guazzelli, E. Scaiella, A. Cesari, A. Battisti, M. Santi, S. Braccini, D. Puppi, V. Paganini, D. Monti, F. Bellina and E. Martinelli, *Int. J. Pharm.*, 2026, **690**, 126538.
